# Controlled Dual Activation of Rhenium(I) Photosensitizers and Profluorophores/Prodrugs via a Dissociative Bioorthogonal Tetrazine–Isonitrile Reaction

**DOI:** 10.1002/anie.202516957

**Published:** 2025-10-21

**Authors:** Eunice Chiu‐Lam Mak, Lawrence Cho‐Cheung Lee, Kenneth Kam‐Wing Lo

**Affiliations:** ^1^ Department of Chemistry City University of Hong Kong Tat Chee Avenue Kowloon Hong Kong P.R. China; ^2^ State Key Laboratory of Terahertz and Millimeter Waves City University of Hong Kong Tat Chee Avenue Kowloon Hong Kong P.R. China

**Keywords:** Bioorthogonal dissociation, Photosensitizers, Prodrug activation, Rhenium, Tetrazine–Isonitrile

## Abstract

Strategies for prodrug activation have been developed to enhance treatment efficacy, with bioorthogonal dissociation reactions emerging as a promising approach due to their remarkable specificity. In this work, we designed three rhenium(I) polypyridine complexes featuring a tetrazylmethyl (TzMe) group capable of bioorthogonal activation by 3‐isocyanopropyl (ICPr) or 3‐isocyanopropyl‐1‐carbamoyl (ICPrc) derivatives. This design serves as a dual‐release platform, which liberates rhenium(I) 3‐hydroxypyridine complexes and functional payloads from rhenium(I) TzMe complexes and ICPr/ICPrc‐caged compounds, respectively. Upon incubation with an ICPrc derivative, the TzMe complexes exhibited strong emission in acidic buffers, attributed to the predominant existence of the resulting rhenium(I) 3‐hydroxypyridine complexes in their protonated form. Confocal imaging of live cells incubated with a TzMe complex and ICPr‐caged fluorescein unveiled intense intracellular emission in distinct channels. Importantly, the therapeutic potential of this approach was underscored by the treatment of cells with a TzMe complex and ICPrc‐caged doxorubicin. The anticancer effect was amplified through the synergy between singlet oxygen (^1^O_2_) photosensitization and prodrug activation, effectively combining photodynamic therapy with chemotherapy. The more pronounced ^1^O_2_ generation of the 3‐hydroxypyridine complexes in acidic media and their specific accumulation within the acidic lysosomes of cancer cells highlight the potential of bioorthogonal prodrug activation for effective cancer‐targeted therapy.

## Graphical abstract

Rhenium(I) tetrazylmethyl complexes serve as phosphorogenic bioorthogonal reagents, releasing luminescent and photocytotoxic rhenium(I) 3‐hydroxypyridine complexes and functional payloads upon reaction with 3‐isocyanopropyl derivatives. Their selective lysosomal accumulation in cancer cells and enhanced singlet oxygen photosensitization in acidic environments highlight their potential for bioimaging and combination therapy in tumors.

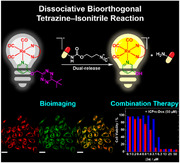

## Introduction

In the past decade, considerable efforts have been devoted to developing methods for the controlled release of a diverse array of molecules within living systems. These molecules include biomacromolecules, bioactive compounds, reporter groups, prodrugs, and antibody–drug conjugates, all of which play pivotal roles in the fields of diagnostics and therapeutics.^[^ ^1^, ^2^ ^]^ As precision medicine continues to evolve, the precise manipulation of spatiotemporal dynamics governing the delivery, release, and activation of these payloads has become a fundamental requirement. Specifically, prodrug activation strategies have been devised to optimize bioavailability, reduce systemic toxicity, and enable the targeted delivery of therapeutic agents.^[^ ^3^, ^4^ ^]^ In this process, an inactive drug derivative is converted into its active form in response to specific endogenous (e.g., enzymatic activity, pH, and glutathione)^[^ ^5^ ^]^ or exogenous stimuli (e.g., light, radiation, and ultrasound).^[^ ^6^, ^7^, ^8^, ^9^ ^]^ Prodrugs can be integrated into self‐assembled structures or encapsulated in nanoparticles to enhance drug accumulation by leveraging the enhanced permeability and retention effect. However, endogenous stimuli can result in poor specificity and selectivity in prodrug activation due to factors such as tumor heterogeneity and minimal differences between normal and cancerous tissues, leading to severe side effects and limited therapeutic efficacy.^[^ ^10^ ^]^ To address these challenges, the integration of bioorthogonal reactions utilizing external stimuli offers compelling alternatives that overcome the limitations associated with endogenous triggers.^[^ ^11^ ^]^

The strategic deployment of bioorthogonal dissociation reactions represents a promising approach for the targeted delivery of functional payloads within living systems.^[^ ^12^, ^13^ ^]^ These reactions are often designed as extensions of the “click‐to‐release” bioorthogonal ligation reactions, typically resulting in conjugation intermediates that can dissociate to release payloads. Notably, the bioorthogonal chemistry between tetrazines and isonitriles has gained attention due to the structural compactness of the isonitrile group, which minimizes disruption to the native biological environment.^[^ ^14^ ^]^ This reaction involves the inverse electron‐demand Diels–Alder (IEDDA) [4 + 1] cycloaddition of 3‐isocyanopropyl (ICPr) or 3‐isocyanopropyl‐1‐carbamoyl (ICPrc)‐caged compounds with tetrazines, followed by rapid expulsion of N_2_ and tautomerization into an imine intermediate. Subsequent hydrolysis to a 3‐oxypropanal derivative induces a spontaneous β‐elimination at the C‐1 position, releasing the desired phenol or amine cargo from the ICPr/ICPrc protecting group.^[^ ^15^ ^]^ Previously, a targeted therapeutic strategy was developed using an ICPr‐caged distyryl boron dipyrromethene‐based photosensitizer, which dissociates to release the active photosensitizer upon reacting with cancer‐targeting tetrazine derivatives.^[^ ^16^ ^]^ Furthermore, the versatility of the bioorthogonal reaction has been expanded to enable the dual release of fluorophores through a single reaction in vivo, which involves modifying the tetrazine moiety into a tetrazylmethyl (TzMe) or tetrazylmethyloxycarbonyl (Tzmoc) protecting group.^[^ ^17^ ^]^ The TzMe or Tzmoc group is removed upon reaction with isonitrile via cycloaddition, N_2_ elimination, and tautomerization, followed by hydrolysis into 4‐aminopyrazole and 1,4‐elimination, ultimately releasing a free phenol or amine in near‐quantitative yields. To date, this dual‐release mechanism has been primarily reported for the simultaneous release of fluorophores,^[^ ^17^, ^18^ ^]^ facilitating bioimaging across distinct channels. However, the design of systems enabling the concurrent release of two cytotoxic payloads from TzMe/Tzmoc‐ and ICPr/ICPrc‐caging groups for combined therapeutic applications is yet to be thoroughly explored.

**Scheme 1:**
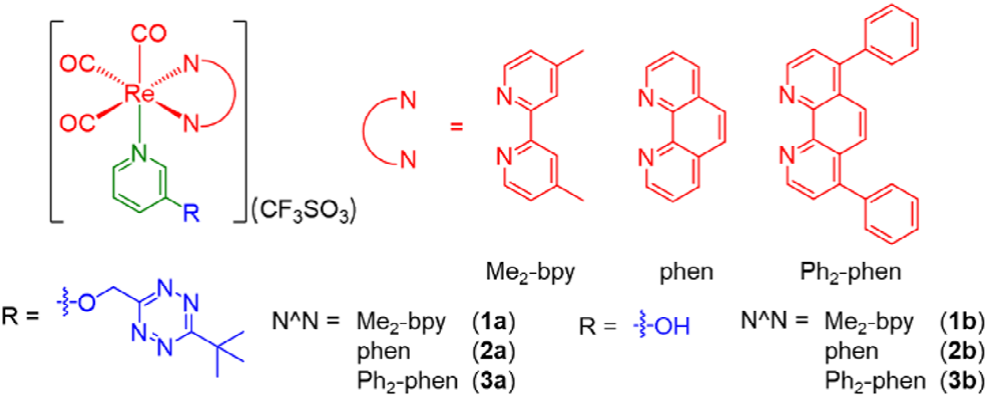
Structures of the rhenium(I) TzMe complexes **1a**–**3a** and 3‐hydroxypyridine complexes **1b**–**3b**.

Our group has applied bioorthogonal ligation chemistry to transition metal complexes by incorporating the tetrazine moiety into diimine (N^N) and cyclometalating (N^C) ligands.^[^ ^19^, ^20^, ^21^, ^22^, ^23^, ^24^ ^]^ These complexes demonstrate a phosphorogenic response and enhanced photocytotoxic effects, driven by increased singlet oxygen (^1^O_2_) photosensitization upon reacting with derivatives of strained alkenes and alkynes, such as *trans*‐cyclooctene (TCO) and (1*R*,8*S*,9*s*)‐bicyclo[6.1.0]nonyne (BCN). Recently, our focus has shifted toward exploring bioorthogonal dissociation chemistry, leading to the development of rhenium(I) complexes with a tetrazine moiety tethered via a bioorthogonally activatable carbamate linker.^[^ ^25^ ^]^ The dissociation of these complexes is initiated by an IEDDA cycloaddition with TCO, which triggers a 1,4‐elimination of the carbamate linker, yielding strongly emissive rhenium(I) aminomethylpyridine complexes with high ^1^O_2_ photosensitization efficiencies. Notably, one of these complexes, featuring a bioorthogonally cleavable poly(ethylene glycol) pendant, exhibits excellent biocompatibility and enhanced photocytotoxic activity following pretreatment with TCO. Despite these advancements, the bioorthogonal release involving multiple functional payloads from transition metal complexes in a single reaction remains unexplored. We envision that combining the cutting‐edge tetrazine–isonitrile chemistry with our expertise in transition metal complexes will lead to innovative phosphorogenic bioorthogonal probes capable of dual payload release through a single two‐component reaction. This strategy holds significant promise for advancing bioimaging and cancer‐targeted therapy. In this work, we designed, synthesized, and characterized three rhenium(I) polypyridine complexes incorporating a TzMe group [Re(N^N)(CO)_3_(py‐OCH_2_‐Tz‐*^t^* Bu)](CF_3_SO_3_) (py‐OCH_2_‐Tz‐*^t^* Bu = 3‐(*tert*‐butyl)‐6‐((pyridin‐3‐yloxy)methyl)‐1,2,4,5‐tetrazine; N^N = 4,4′‐dimethyl‐2,2′‐bipyridine (Me_2_‐bpy) (**1a**), 1,10‐phenanthroline (phen) (**2a**), and 4,7‐diphenyl‐1,10‐phenanthroline (Ph_2_‐phen) (**3a**)) (Scheme 1). Their TzMe‐free counterparts [Re(N^N)(CO)_3_(py‐OH)](CF_3_SO_3_) (py‐OH = 3‐hydroxypyridine; N^N = Me_2_‐bpy (**1b**), phen (**2b**), and Ph_2_‐phen (**3b**)) were prepared for comparative analysis. The photophysical and photochemical properties of these complexes were studied. The bioorthogonal reactivity and phosphorogenic response of the TzMe complexes toward an ICPrc derivative, 3‐isocyanopropyl benzylcarbamate (ICPrc‐Bn), were investigated. Additionally, the intracellular localization, cellular uptake, and (photo)cytotoxicity of the TzMe complexes with and without ICPrc‐Bn treatment were examined. Furthermore, the potential for dual payload release utilizing functional ICPr/ICPrc derivatives was explored for diagnostic and therapeutic applications.

## Results and Discussion

### Synthesis and Characterization of the Rhenium(I) Complexes

The ligand py‐OCH_2_‐Tz‐*^t^* Bu was synthesized through a three‐step procedure (Scheme S1). Initially, py‐OH was reacted with bromoacetonitrile to yield 3‐(cyanomethoxy)pyridine. This intermediate was then subjected to a reaction with trimethylacetonitrile in an EtOH solution in the presence of hydrazine monohydrate and sulfur as a catalyst under mild heat and a N_2_ atmosphere. This was followed by oxidation with sodium nitrite, resulting in the formation of the asymmetric tetrazine moiety. The ligand was subsequently purified via column chromatography, producing a distinctive purple oil. The rhenium(I) complexes were prepared by refluxing the rhenium(I) precursors [Re(N^N)(CO)_3_(CH_3_CN)](CF_3_SO_3_) with either py‐OCH_2_‐Tz‐*^t^* Bu or py‐OH in THF. The resulting mixtures were purified through column chromatography and recrystallization from CH_2_Cl_2_/Et_2_O, yielding dark pink and yellow crystals, respectively. These complexes were thoroughly characterized using ^1^H and ^13^C NMR, IR spectroscopy, and HR‐ESI‐MS.

### Photophysical Properties

The electronic absorption spectra and corresponding data for the TzMe complexes **1a**–**3a**, TzMe‐free complexes **1b**–**3b**, and the ligands py‐OCH_2_‐Tz‐*^t^* Bu and py‐OH are presented in Figure S1 and Table S1, respectively. All complexes exhibited intense spin‐allowed intraligand (^1^IL) (π → π*) (N^N and pyridine) absorption bands at around 250–340 nm and weaker spin‐allowed metal‐to‐ligand charge‐transfer (^1^MLCT) (dπ(Re) → π*(N^N)) absorption bands/shoulders at around 350–430 nm.^[^ ^22^, ^25^, ^26^, ^27^, ^28^ ^]^ The weak absorption band at around 537–550 nm of complexes **1a**–**3a** is assigned to the *n* → π* transition of the tetrazine moiety.^[^ ^21^ ^]^ The emission spectra and photophysical data of the complexes are presented in Figure S2 and Table S2, respectively. Upon photoexcitation, all the complexes exhibited greenish‐yellow to orange emission in solutions under ambient conditions and in low‐temperature alcohol glass. Notably, complexes **1a**–**3a** exhibited significantly lower emission quantum yields (*Φ*_em_ = 0.002–0.020 in CH_2_Cl_2_) than complexes **1b**–**3b** (*Φ*_em_ = 0.15–0.43 in CH_2_Cl_2_) (Table S2) and common rhenium(I) polypyridine complexes,^[^ ^26^, ^28^ ^]^ indicative of efficient emission quenching by the tetrazine moiety.^[^ ^22^, ^25^ ^]^ Complexes **2a**, **3a**, and **1b**–**3b** showed broad and structureless emission bands in solutions at 298 K (Figure S2). The emission spectra of these complexes experienced a bathochromic shift as the solvent polarity increased from CH_2_Cl_2_ to CH_3_CN, and their emission maxima displayed a significant hypsochromic shift upon cooling the samples to 77 K, indicative of the involvement of ^3^MLCT (dπ(Re) → π*(N^N)) character in their excited states.^[^ ^25^, ^26^, ^27^, ^28^, ^29^ ^]^ In contrast, complex **1a** showed an emission band with vibronic structure in solutions at 298 K (Figure S2). Also, the vibrational progressional spacing (energy difference between vibrational energy levels) was approximately 2000 cm^−1^ in CH_2_Cl_2_, which is likely associated with the aromatic vibrational modes of the diimine ligand. These findings suggested the possible involvement of some ^3^IL (π → π*) (Me_2_‐bpy) character in the excited state of the complex.^[^ ^25^, ^26^, ^27^, ^28^, ^29^ ^]^ Alternatively, the sharply reduced emission intensities of complexes **1a** and **2a**, and to a lesser extent for complex **3a** at approximately 550 nm in alcohol glass (Figure S2) could be attributed to self‐absorption arising from the inner‐filter effect of the tetrazine ligand.^[^ ^21^ ^]^ Furthermore, the emission lifetimes of complexes **1a**–**3a** are comparable to or longer than those of complexes **1b**–**3b** (Table S2), likely due to the relatively modest quenching effect of the nonconjugated tetrazine group, as opposed to direct conjugation with the metal center, combined with the hydrophobic nature of the tetrazine moiety.

### Bioorthogonal Reactivity Toward Isonitriles

**Scheme 2:**
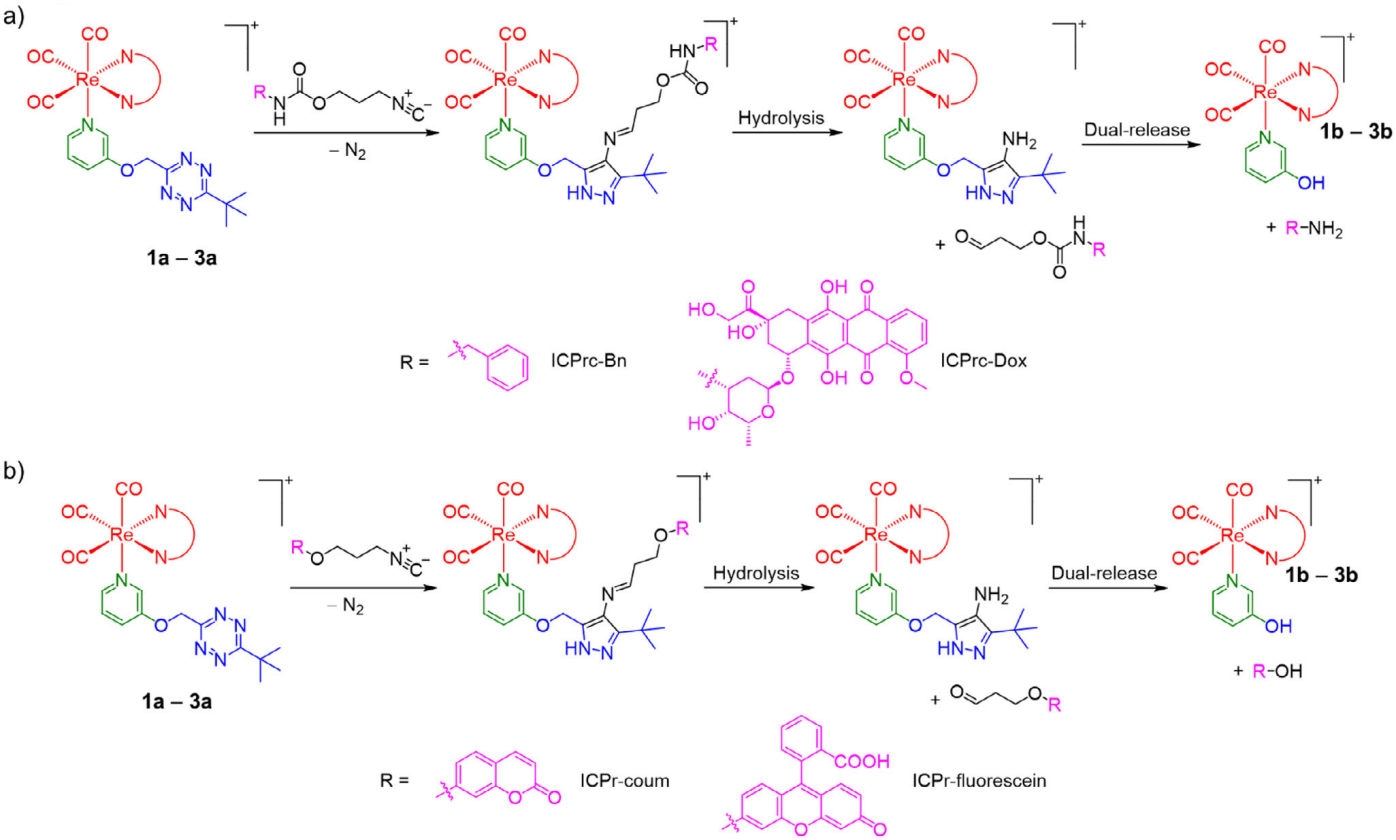
Bioorthogonal reaction of complexes **1a**–**3a** with a) ICPrc and b) ICPr derivatives, leading to dual release of complexes **1b**–**3b** and amine/phenol cargo.

**Figure 1:**
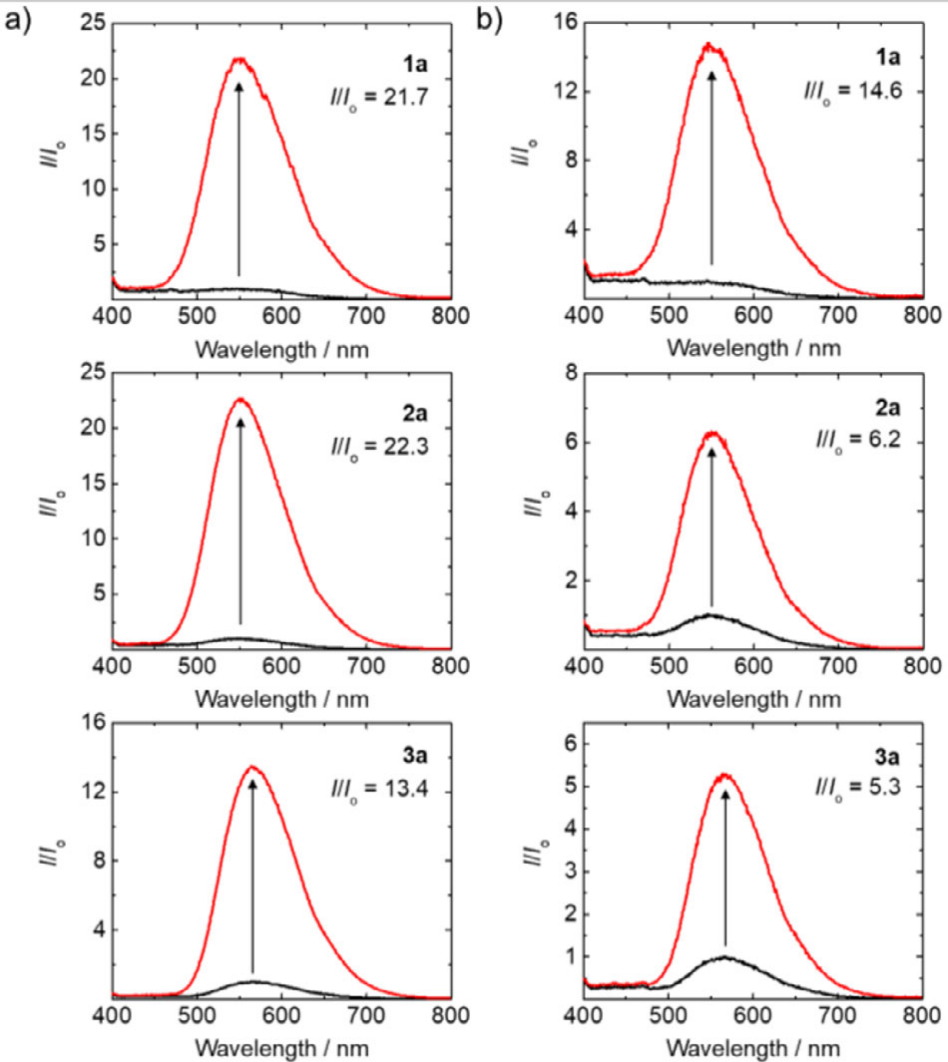
Emission spectra of complexes **1a**–**3a** (10 µM) before (black) and after (red) incubation with ICPrc‐Bn (500 µM) in aerated a) H_2_O/DMSO (4:1, *v*/*v*) and b) McIlvaine buffer (pH 5.0)/DMSO (4:1, *v*/*v*) at 37 °C for 18 h.

The bioorthogonal reactivity and phosphorogenic response of the TzMe complexes **1a**–**3a** toward ICPrc‐Bn were investigated, as illustrated in Scheme 2a. The bioorthogonal dissociation reaction of these complexes, leading to the formation of complexes **1b**–**3b** as final products, was confirmed through ESI‐MS analysis (Figure S3). Upon reaction of complexes **1a**–**3a** (10 µM) with ICPrc‐Bn (500 µM) in aerated H_2_O/DMSO (4:1, *v*/*v*) at 37 °C, substantial emission enhancement (*I*/*I*_o_ = 13.4–22.3) was observed (Figure 1a and Table S3). Interestingly, when the reaction was conducted in aerated McIlvaine buffer (pH 5.0)/DMSO (4:1, *v*/*v*), the solutions exhibited apparent emission enhancement (*I*/*I*_o_ = 5.3–14.6; Figure 1b and Table S4). In contrast, no emission enhancement was detected in aerated McIlvaine buffer (pH 7.4)/DMSO (4:1, *v*/*v*) (Table S5 and Figure S4). Additionally, the reactivity of complex **3a** (10 µM) toward the commonly used dienophile, (1*R*,8*S*,9*s*)‐bicyclo[6.1.0]non‐4‐yn‐9‐ylmethanol (BCN‐OH) (500 µM), in aerated H_2_O/DMSO (4:1, *v*/*v*) was examined using ESI‐MS (Figure S5), which revealed negligible reaction after incubation for 18 h. This highlights the specificity of TzMe complexes toward ICPr/ICPrc derivatives. This also suggests the potential for TzMe complexes and ICPr/ICPrc compounds to be used orthogonally alongside other bioorthogonal reaction pairs without cross‐reactivity, enabling simultaneous and selective chemical transformations in biological systems. The observed phenomena of the TzMe complexes with ICPrc‐Bn can be attributed to the pH‐dependent emission properties of the resulting products, complexes **1b**–**3b**, which have p*K*_a_ values of around 6.5–7.0.^[^ ^30^, ^31^, ^32^ ^]^ In acidic environments, the rhenium(I) 3‐hydroxypyridine complexes **1b**–**3b** exist in a protonated state, while in neutral to alkaline conditions, they adopt a deprotonated state. This behavior would likely lead to rapid nonradiative decay through reductive quenching by pyridinolate. Consequently, the TzMe complexes demonstrate potential as pH‐sensitive phosphorogenic bioorthogonal probes in biological systems, providing a valuable tool for detecting pH variations in acidic tumor microenvironments (TME).^[^ ^33^ ^]^

The ^1^O_2_ generation efficiencies of the complexes were evaluated by monitoring the emission band of ^1^O_2_ centered at around 1270 nm^[^ ^34^, ^35^ ^]^ in aerated CH_3_CN. The TzMe complexes **1a**–**3a** showed comparable ^1^O_2_ generation quantum yields (*Φ*_Δ_ = 0.42–0.66) to the TzMe‐free complexes **1b**–**3b** (*Φ*_Δ_ = 0.36–0.63) (Table S6), which is attributed to the formation of a triplet charge‐separated state of the TzMe complexes upon photoexcitation.^[^ ^21^, ^25^ ^]^ Considering the pH‐dependent emission properties of complexes **1b**–**3b**, we believe that their ^1^O_2_ photosensitization efficiencies would also be influenced by pH. Taking complex **3b** as an example, its ^1^O_2_ generation efficiency was evaluated in aerated McIlvaine buffer solutions at various pH levels by the 9,10‐anthracenediyl‐*bis*(methylene)dimalonic acid (ABDA) assay.^[^ ^36^ ^]^ At pH 7.4, complex **3b** generated minimal ^1^O_2_; however, lowering the pH to 5.0 and 3.0 resulted in increased ^1^O_2_ quantum yields of 0.12 and 0.24, respectively (Table S7 and Figure S6). In contrast, complex **3a** demonstrated consistent ^1^O_2_ generation efficiency (*Φ*_Δ_ = 0.33–0.36) regardless of pH. Thus, the TzMe complexes are anticipated to function as pH‐sensitive phosphorogenic bioorthogonal probes, enabling simultaneous detection and photodynamic therapy in lysosomal compartments (pH ≈ 4.5–5.0)^[^ ^37^, ^38^ ^]^ and acidic TME.

**Figure 2:**
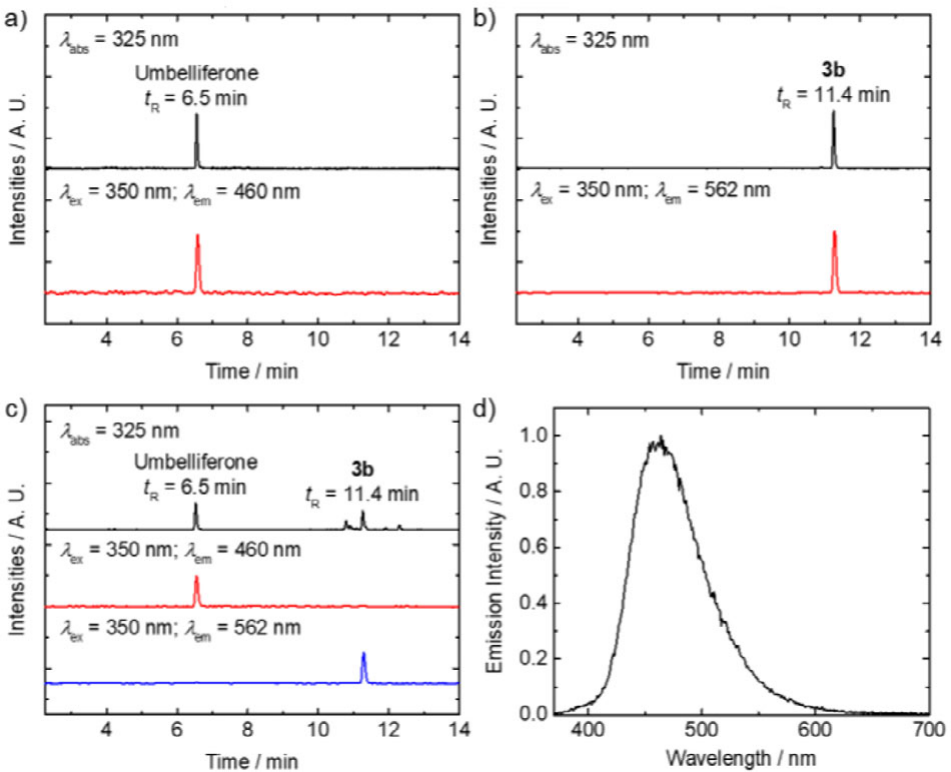
HPLC chromatograms of: a) umbelliferone, b) complex **3b**, and c) the reaction mixture containing complex **3a** (50 µM) and ICPr‐coum (40 µM) in aerated McIlvaine buffer (pH 7.4)/DMSO (4:1, *v*/*v*) after incubation at 37 °C for 18 h. The absorbance was monitored at 325 nm. The emission was monitored at 460 nm and 562 nm (*λ*_ex_ = 350 nm). d) Emission spectrum of the product collected at *t*_R_ = 6.5 min (*λ*_ex_ = 325 nm).

The reactivity of the TzMe complexes **1a**–**3a** and the ligand py‐OCH_2_‐Tz‐*^t^* Bu with ICPr/ICPrc derivatives was explored using ICPr‐coumarin (ICPr‐coum) as a model compound (Scheme 2b). HPLC analyses unveiled a clean and efficient bioorthogonal dissociation of the TzMe complexes into complexes **1b**–**3b**, with near‐quantitative conversions in both neutral (pH 7.4) and slightly acidic (pH 5.0) buffer solutions (Figure S7). For instance, with complex **3a**, the initial peak at *t*_R_ = 12.3 min disappeared, and a new peak at *t*_R_ = 11.3 min emerged in the chromatogram after 18 h of incubation with ICPr‐coum. Beyond cycloaddition kinetics and stability considerations, the release kinetics and the nature of the released moiety are critical parameters. The reaction kinetics of complexes **1a**–**3a** (20 µM) with ICPr‐coum (25 µM) was studied in buffer solutions at 37 °C by monitoring the reaction at different time intervals using HPLC. The absence of [4 + 1] cycloaddition intermediates indicated that the cycloaddition reaction is the rate‐determining step, whereas the release step occurs rapidly. The second‐order rate constants (*k*_2_) for these reactions ranged from 7.6 to 22.5 M^−1^ s^−1^ in a neutral buffer solution, following the order: **2a** < **1a** < **3a** (Figure S8a). Similarly, in a slightly acidic buffer solution, the *k*_2_ values (ranged from 7.0 to 22.1 M^−1^ s^−1^) followed the same trend (Figure S8b), indicating that the acidity of the environment has minimal impact on the bioorthogonal dissociation efficiencies of the TzMe complexes. Additionally, to evaluate the dual‐release properties of the reaction, the reaction between complex **3a** and ICPr‐coum was monitored using HPLC and spectrophotometric analyses. After incubating the reaction mixture of complex **3a** and ICPr‐coum for 18 h, an aliquot was quenched and subjected to HPLC analysis. Emission was monitored at the emission maxima of the released products, umbelliferone (460 nm) (Figure 2a) and complex **3b** (562 nm) (Figure 2b). Peaks at *t*_R_ = 6.5 and 11.4 min, corresponding to umbelliferone and complex **3b**, respectively, were identified (Figure 2c). The emission spectrum of the fraction collected at *t*_R_ = 6.5 min matched that of umbelliferone (Figure 2d). Furthermore, the half‐lives and release yields of ICPr‐coum were determined by adding an excess of TzMe complexes **1a**–**3a** (50 µM) to ICPr‐coum (20 µM). The reaction facilitated the conversion of ICPr‐coum (*t*_R_ = 8.3 min) into umbelliferone (*t*_R_ = 6.5 min) via an intermediate (*t*_R_ = 7.4 min), with release rates (half‐lives) spanning from 0.66 to 1.50 h and release yields exceeding 95% in neutral buffer conditions (Figures S9a and S10a). Remarkably, the release rates and yields from ICPr‐coum were consistent with the order observed for the dissociation of the complexes. However, under slightly acidic buffer conditions, the release rates showed a modest decrease, ranging from 2.13 to 3.94 h, while the release yields remained above 80% (Figures S9b and S10b). The reduction in yields could potentially be attributed to the sensitivity of the isonitrile group to acidic environments.^[^ ^39^, ^40^ ^]^ The reaction kinetics and half‐lives of complexes **1a**–**3a** in both neutral and acidic buffer solutions were significantly higher than those of the uncoordinated ligand py‐OCH_2_‐Tz‐*^t^* Bu (Figures S11–14), owing to the electron‐withdrawing effect of the cationic rhenium(I) center, which facilitates a more rapid and efficient reaction.^[^ ^19^, ^20^, ^21^, ^22^, ^23^, ^24^, ^41^ ^]^

### Bioorthogonal Labeling and Localization in Live Cells

**Figure 3:**
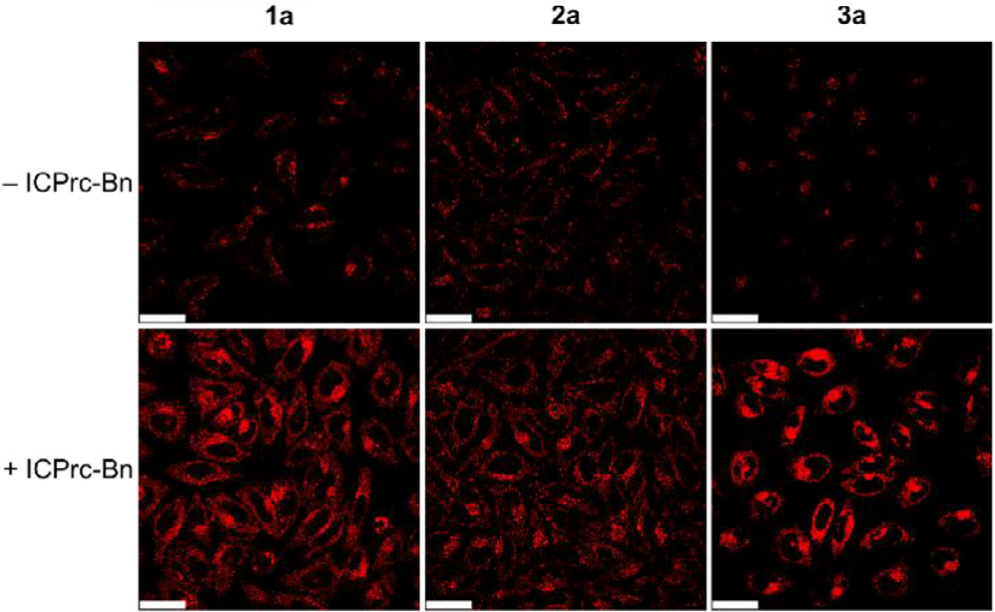
LSCM images of HeLa cells incubated with complexes **1a**–**3a** (10 µM, 3 h, *λ*_ex_ = 405 nm, *λ*_em_ = 500–600 nm for complexes **1a** and **2a** and 550−600 nm for complex **3a**), followed by incubation with ICPrc‐Bn (200 µM, 4 h) or fresh DMEM (4 h) at 37 °C. Scale bar = 25 µm.

**Figure 4:**
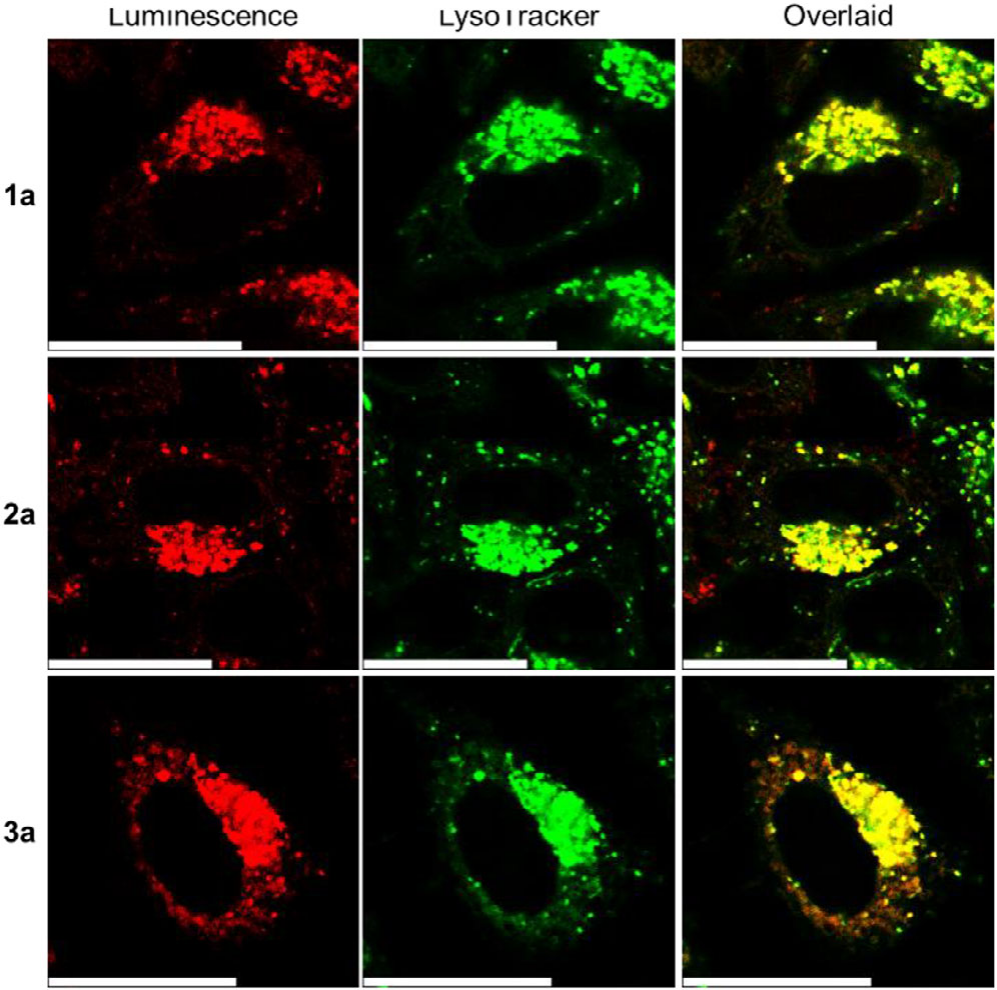
LSCM images of HeLa cells incubated with complexes **1a**–**3a** (10 µM, 3 h, *λ*_ex_ = 405 nm, *λ*_em_ = 500–600 nm for complexes **1a** and **2a** and 550−600 nm for complex **3a**) and ICPrc‐Bn (200 µM, 4 h), and further incubated with LysoTracker Deep Red (75 nM, 1 h, *λ*_ex_ = 635 nm, *λ*_em_ = 650−670 nm) at 37 °C. PCC = 0.89 (complex **1a**), 0.87 (complex **2a**), and 0.89 (complex **3a**). Scale bar = 25 µm.

The bioorthogonal reactivity of complexes **1a**–**3a** with ICPr/ICPrc derivatives in live cells was investigated using HeLa cells as a model system. Initially, the cells were incubated with complex **3a** (10 µM) for 3 h, followed by treatment with ICPrc‐Bn (200 µM) for 4 h, or with fresh medium as a control. ESI mass spectra of organic extracts from the cell lysates revealed complete bioorthogonal dissociation of complex **3a** into complex **3b** upon treatment with ICPrc‐Bn (Figure S15a), while complex **3a** remained stable in the absence of ICPrc‐Bn in fresh medium (Figure S15b). These optimized incubation conditions were subsequently applied in other cellular experiments. The phosphorogenic response of complexes **1a**–**3a** toward ICPrc‐Bn in live HeLa cells was studied using laser‐scanning confocal microscopy (LSCM) and flow cytometry. Cells not treated with ICPrc‐Bn exhibited very weak intracellular emission (Figures 3 and S16 and Table S8) due to the quenching effect of the tetrazine moiety in the complexes. In contrast, cells treated with ICPrc‐Bn showed intense intracellular emission (Figures 3 and S16 and Table S8), indicating efficient removal of the tetrazine moiety and release of the emissive rhenium(I) 3‐hydroxypyridine complexes **1b**–**3b**. Co‐staining experiments with LysoTracker Deep Red (75 nM, 1 h) and MitoTracker Deep Red (100 nM, 20 min) confirmed that the released complexes **1b**–**3b** predominantly accumulated in acidic lysosomes, as evidenced by a high Pearson's correlation coefficient (PCC) of 0.87–0.89 (Figure 4), but not in mitochondria (PCC = 0.17–0.56; Figure S17).

### Cellular Uptake and (Photo)Cytotoxicity Studies

The cellular uptake efficiencies of the complexes in cancerous HeLa and normal HEK‐293 cells were evaluated using inductively coupled plasma‐mass spectrometry (ICP‐MS). The order of cellular uptake was established as **1a** ≈ **2a** < **3a** for both cell lines, with all complexes showing greater internalization in HeLa cells ([Re] = 2.6–9.2 fmol) compared to HEK‐293 cells ([Re] = 0.61–3.8 fmol) (Table S9). Notably, complex **3a** exhibited significantly higher cellular uptake than complexes **1a** and **2a**, attributed to the more hydrophobic nature of the Ph_2_‐phen ligand. The highest cellular uptake of complex **3a** correlated with the most pronounced emission enhancement observed in flow cytometric studies (Table S8 and Figure S16) among the TzMe complexes. Furthermore, the cellular uptake mechanism of complex **3a** was studied. When HeLa cells were pre‐exposed to low‐temperature conditions (4 °C) or various endocytosis inhibitors, including clathrin‐mediated endocytosis inhibitor chlorpromazine (30 µM, 1 h), macropinocytosis inhibitor 5‐(*N*‐ethyl‐*N*‐isopropyl)amiloride (EIPA) (50 µM, 1.5 h), and caveolin‐mediated endocytosis inhibitor methyl‐β‐cyclodextrin (Me‐β‐CD) (5 mM, 1 h), followed by treatment with complex **3a** (10 µM, 1 h), there was no significant reduction in cellular uptake efficiency (< 12%) (Figure S18). These findings suggest that complex **3a** was primarily internalized into the cells through energy‐independent passive or facilitated diffusion,^[^ ^42^, ^43^, ^44^, ^45^ ^]^ rather than energy‐dependent pathways such as endocytosis. This behavior is likely attributed to the high lipophilicity of the complex, which facilitates rapid membrane permeation into the cytosol. The lysosomal accumulation of complex **3a** following post‐treatment with ICPrc‐Bn may result from diffusion across the lysosomal membrane or autophagic engulfment, followed by entrapment within lysosomes upon conversion to complex **3b**. In the acidic lysosomal environment, complex **3b** primarily exists in its protonated form, promoting its retention.

The (photo)cytotoxicity of complexes **1a**–**3a** in live cells, both with and without post‐treatment of ICPrc‐Bn, was investigated using the 3‐(4,5‐dimethylthiazol‐2‐yl)‐2,5‐diphenyltetrazolium bromide (MTT) assay. HeLa and HEK‐293 cells incubated with ICPrc‐Bn (200 µM) alone for 4 h and subsequently subjected to irradiation showed cell viability greater than 98%, confirming the negligible (photo)cytotoxicity of ICPrc‐Bn. Notably, complexes **1a** and **2a** demonstrated minimal (photo)cytotoxicity (IC_50,dark_ and IC_50,light_ > 50 µM; Table 1) in both HeLa and HEK‐293 cells, regardless of the presence of ICPrc‐Bn. This can be attributed to the moderate cellular uptake of the complexes (Table S9) and their relatively lower efficiency in ^1^O_2_ photosensitization (Table S6). These findings underscore the commendable biocompatibility of the complexes, rendering them promising phosphorogenic probes for bioimaging applications. Conversely, complex **3a** exhibited significant photocytotoxicity in HeLa cells, irrespective of the presence of ICPrc‐Bn (IC_50,light_ = 2.0 and 2.4 µM), due to the substantially higher cellular uptake efficiency of complex **3a** in HeLa cells (Table S9) and superior ^1^O_2_ photosensitization capabilities of complexes **3a** and **3b** (Table S6). The photocytotoxicity of complex **3a**, both in the absence and presence of ICPrc‐Bn, was significantly reduced in HEK‐293 cells (IC_50,light_ = 6.6 and 7.5 µM, respectively), attributed to its limited cellular uptake efficiency in these cells (Table S9). This signifies the potential of complex **3a** as a promising phototherapeutic agent for cancer treatment, leveraging its ability to selectively target and eradicate cancerous cells while minimizing effects on normal cells.

**Table 1:** (Photo)cytotoxicity of complexes **1a**–**3a** and cisplatin toward HeLa and HEK‐293 cells without or with ICPrc‐Bn post‐treatment in the dark and upon irradiation at 365 nm (5 mW cm^−2^) for 5 min.^a)^ Photocytotoxicity index (PI) is the ratio IC_50,dark_/IC_50,light_.

	HeLa						HEK‐293					
	– ICPrc‐Bn			+ ICPrc‐Bn			– ICPrc‐Bn			+ ICPrc‐Bn		
Complex	IC_50,dark_ [µM]	IC_50,light_ [µM]	PI	IC_50,dark_ [µM]	IC_50,light_ [µM]	PI	IC_50,dark_ [µM]	IC_50,light_ [µM]	PI	IC_50,dark_ [µM]	IC_50,light_ [µM]	PI
**1a**	> 50	> 50	–^b)^	> 50	> 50	–^b)^	> 50	> 50	–^b)^	> 50	> 50	–^b)^
**2a**	> 50	> 50	–^b)^	> 50	> 50	–^b)^	> 50	> 50	–^b)^	> 50	> 50	–^b)^
**3a**	17 ± 1	2.0 ± 0.2	9	12 ± 1	2.4 ± 0.1	5	44 ± 4	6.6 ± 0.6	7	26 ± 3	7.5 ± 0.6	3
Cisplatin	> 50	> 50	–^b)^				> 50	> 50	–^b)^			

^a)^ The cells were first treated with the complexes for 3 h, and then incubated with either fresh growth medium or ICPrc‐Bn (200 µM) in the dark for 4 h, replaced with fresh growth medium, followed by incubation in the dark or exposure to irradiation in fresh growth medium. Cells incubated with cisplatin for 3 h served as a positive control.

^b)^ Could not be determined with accuracy.

### Dual Release of Functional Payloads in Live Cells

**Figure 5:**
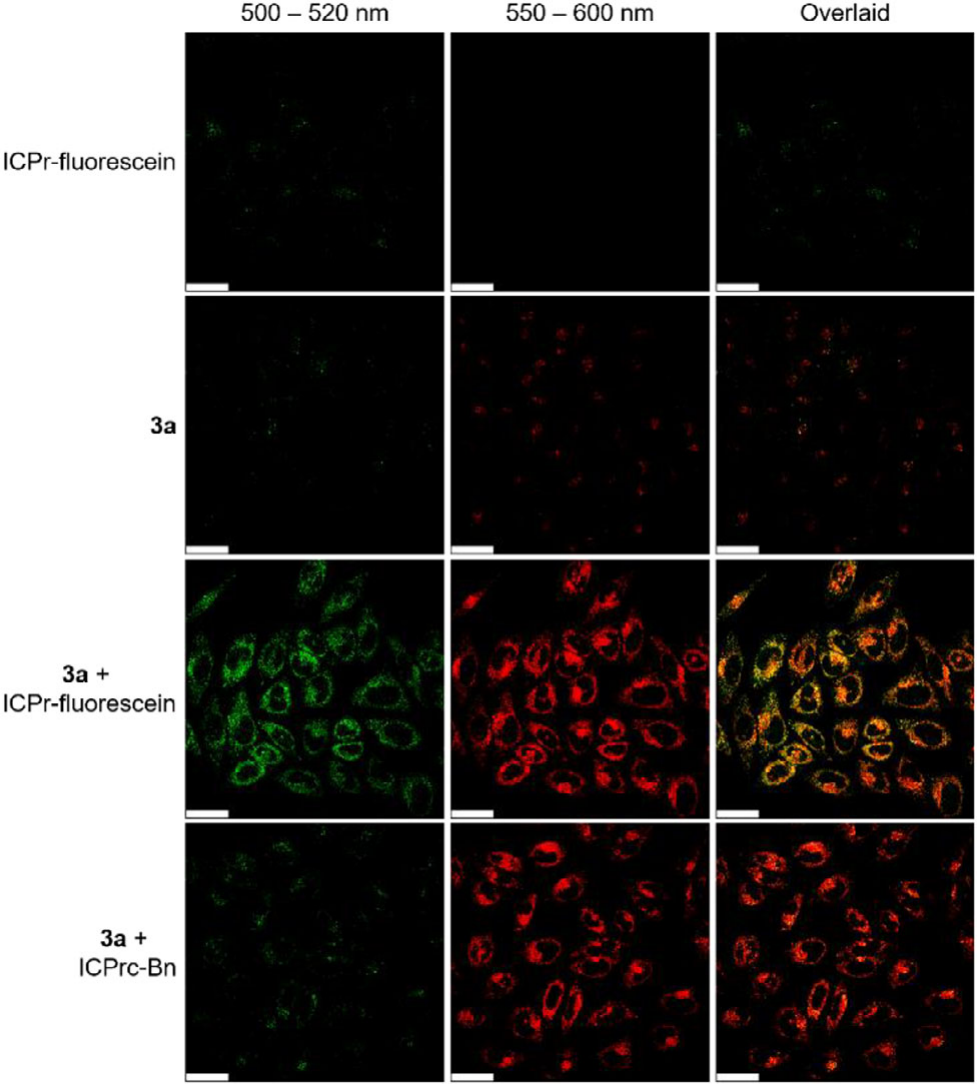
LSCM images of HeLa cells incubated with ICPr‐fluorescein (10 µM, 4 h, *λ*_ex_ = 488 nm, *λ*_em_ = 500–520 nm) alone, or incubated with complex **3a** (10 µM, 3 h, *λ*_ex_ = 405 nm, *λ*_em_ = 550–600 nm), followed by incubation without or with ICPr‐fluorescein (10 µM, 4 h), or incubation with ICPrc‐Bn (200 µM, 4 h) at 37 °C. PCC = 0.71 (complex **3a** + ICPr‐fluorescein). Scale bar = 25 µm.

The initial study of dual functional payload release in live cells was conducted using complex **3a** and ICPr‐coum. LSCM images illustrated weak emission from ICPr‐coum (*λ*_ex_ = 405 nm, *λ*_em_ = 430–500 nm) or complex **3a** (*λ*_ex_ = 405 nm, *λ*_em_ = 550–600 nm) when each was administered individually (Figure S19). However, when HeLa cells were treated with complex **3a** (10 µM) followed by ICPr‐coum (200 µM), a noticeable increase in intracellular emission was observed in both channels. This suggests that a bioorthogonal reaction occurred, leading to the release of complex **3b** and umbelliferone. The emission enhancement in the ICPr‐coum channel was somewhat subdued due to the weak absorption of umbelliferone at the 405 nm excitation wavelength. To optimize the dual‐release phenomenon in live cells, another ICPr‐modified fluorophore, ICPr‐fluorescein (Scheme 2b), was synthesized and characterized. The profluorophore remained stable in serum for at least 48 h (Figure S20a). HPLC analyses confirmed the dual release of complex **3b** and free fluorescein upon incubation with complex **3a** and ICPr‐fluorescein (Figure S21). LSCM images revealed that ICPr‐fluorescein (*λ*_ex_ = 488 nm, *λ*_em_ = 500–520 nm) exhibited weak emission on its own, attributed to quenching by the ICPr cage (Figure 5). However, when HeLa cells were treated with both complex **3a** (10 µM) and ICPr‐fluorescein (10 µM), strong intracellular emission intensities were detected in both channels. The PCC value of 0.71 indicated a close correlation in subcellular distribution of the released payloads. These findings strongly imply the simultaneous bioorthogonal dissociation of complex **3a** and uncaging of ICPr‐fluorescein, resulting in the liberation of complex **3b** and the free fluorophore.

**Figure 6:**
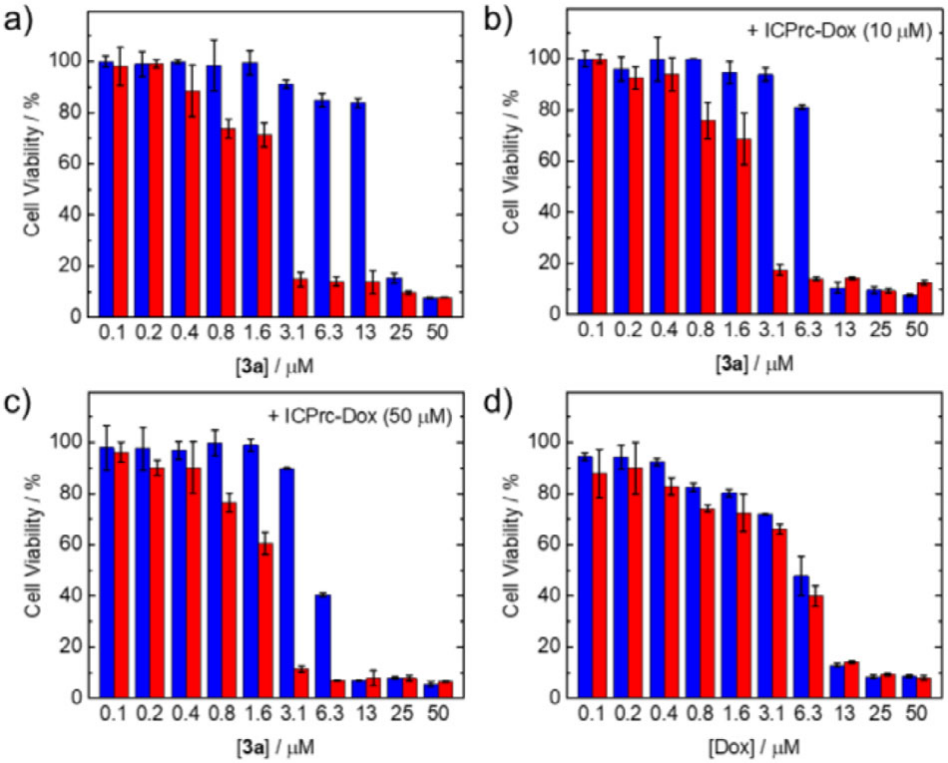
Viability of HeLa cells incubated with complex **3a** for 3 h, and then incubated a) with fresh growth medium, b) ICPrc‐Dox (10 µM), or c) ICPrc‐Dox (50 µM) for 4 h. HeLa cells incubated with d) Dox for 4 h served as a positive control. The cells were further incubated in the dark (blue) or irradiated at 365 nm (5 mW cm^−2^) (red) for 5 min, and then incubated with fresh growth medium for 24 h.

Building on the successful concurrent release of complex **3b** and the fluorophore through bioorthogonal dissociation in live cells, we explored the potential of the TzMe complexes for prodrug activation employing complex **3a** and ICPrc‐modified doxorubicin (Dox), ICPrc‐Dox (Scheme 2a). The ICPrc‐caged prodrug remained completely stable and inert in serum for over 48 h (Figure S20b). The bioorthogonal reaction was assessed via HPLC analyses, which confirmed the dual release of complex **3b** and unbound Dox in the chromatograms (Figure S22). Time‐dependent HPLC traces of equimolar mixtures of complex **3a** and ICPr/ICPrc derivatives are presented in Figure S23a–c. Notably, upon reaction with complex **3a**, the release rates from ICPr‐coumarin, ICPr‐fluorescein, and ICPrc‐Dox were comparable, with half‐lives ranging from 0.27 to 0.68 h (Figure S23d). This indicated that both ICPr and ICPrc derivatives, featuring ether and carbamate linkages, respectively, facilitated rapid payload release. The (photo)cytotoxic effects of the dual release from complex **3a** and the prodrug were examined using the MTT assay. Dox, serving as a positive control, gave similar IC_50_ values in HeLa cells both in the dark (IC_50,dark_ = 5.8 µM) and under irradiation (IC_50,light_ = 5.3 µM). In contrast, in HEK‐293 cells, Dox exhibited minimal cytotoxicity both in the dark (IC_50,dark_ > 50 µM) and under irradiation (IC_50,light_ = 43 µM). The ICPrc‐caged drug (10 or 50 µM) demonstrated negligible (photo)cytotoxicity in both HeLa and HEK‐293 cells, maintaining cell viability at levels exceeding 98%. Following treatment with complex **3a** in light‐exposed conditions, a notable decrease in HeLa cell viability was observed (Figure 6a). Importantly, upon post‐treatment of complex **3a**‐treated HeLa cells with ICPrc‐Dox at 10 and 50 µM, IC_50,dark_ values significantly decreased from 17 to 7.8 and 5.5 µM, respectively (Table 2), approaching the IC_50,dark_ value of the positive control, Dox (5.8 µM), and similar to that of the combination of complex **3b** and Dox in a 1:1 ratio (3.8 µM) (Table S10). For instance, when cells were treated with a lower concentration of complex **3a** (6.3 µM) alone, cell viability was approximately 85% in the dark (Figure 6a). However, post‐treatment with ICPrc‐Dox (50 µM) significantly reduced cell viability to 40% in the dark (Figure 6c). Similarly, treatment of HeLa cells with a higher concentration of complex **3a** (13 µM) followed by ICPrc‐Dox resulted in a dramatic reduction in cell viability from about 84% for the complex alone (Figure 6a) to 11% after treatment with ICPrc‐Dox (10 µM) (Figure 6b) and 7% after treatment with ICPrc‐Dox (50 uM) (Figure 6c) in the dark. The results demonstrated effective prodrug release from ICPrc‐Dox through the bioorthogonal reaction with complex **3a**, with dose‐dependent cytotoxic effects. Upon irradiation, the difference in cell viability with or without ICPrc‐Dox was less pronounced. At a lower concentration of complex **3a** (1.6 µM), cell viability decreased from approximately 71% for the complex alone (Figure 6a) to 60% when treated with ICPrc‐Dox (50 µM) (Figure 6c). At higher concentrations of complex **3a**, cell viability in those treated with ICPrc‐Dox (Figure 6b,c) decreased comparably to what was observed with the complex alone (Figure 6a) and Dox alone (Figure 6d), suggesting that the cytotoxic effects had already reached their peak potential. In HEK‐293 cells, the same treatment with complex **3a** and ICPrc‐Dox resulted in reduced cytotoxic effects both in the dark and under irradiation (Figure S24), attributed to the significantly lower cellular uptake efficiency of complex **3a** in HEK‐293 cells (Table S9). Overall, these findings present promising prospects for combination therapy utilizing TzMe‐containing complexes for prodrug activation in cancer cells and tumors. Future studies will focus on exploring other ICPr/ICPrc‐caged prodrugs to further enhance the efficacy of combination therapy.

**Table 2:** (Photo)cytotoxicity of complex **3a** toward HeLa and HEK‐293 cells without or with ICPrc‐Dox post‐treatment in the dark and upon irradiation at 365 nm (5 mW cm^−2^) for 5 min.^a)^ PI is the ratio IC_50,dark_/IC_50,light_.

	HeLa			HEK‐293		
Entry	IC_50,dark_ [µM]	IC_50,light_ [µM]	PI	IC_50,dark_ [µM]	IC_50,light_ [µM]	PI
**3a**	17 ± 1	2.0 ± 0.2	9	44 ± 4	6.6 ± 0.6	7
**3a** + ICPrc‐Dox (10 µM)	7.8 ± 0.5	1.9 ± 0.2	4	22 ± 1	7.6 ± 0.5	3
**3a** + ICPrc‐Dox (50 µM)	5.5 ± 0.1	1.8 ± 0.1	3	23 ± 2	5.7 ± 0.1	4
Dox	5.8 ± 0.6	5.3 ± 0.6	1	> 50	43 ± 2	> 1

^a)^ The cells were first treated with complex **3a** for 3 h, and then incubated with either fresh growth medium or ICPrc‐Dox (10 or 50 µM) in the dark for 4 h, replaced with fresh growth medium, followed by incubation in the dark or exposure to irradiation in fresh growth medium. Cells incubated with Dox for 4 h served as a positive control.

## Conclusion

Bioorthogonal dissociation reactions have shown exceptional promise for prodrug activation in anticancer therapy. In this study, we developed three rhenium(I) TzMe complexes as phosphorogenic bioorthogonal reagents, creating a dual‐release system upon reaction with ICPr/ICPrc‐caged compounds. This innovative approach concurrently liberates rhenium(I) 3‐hydroxypyridine complexes and functional payloads. The TzMe complexes were weakly emissive in solutions due to emission quenching by the tetrazine moiety. When incubated with ICPr/ICPrc derivatives, the TzMe complexes exhibited strong emission in acidic buffers, attributed to the predominant presence of the resulting 3‐hydroxypyridine complexes in their protonated form, showcasing the pH‐responsive emission properties of the complexes. The dual activation demonstrated high reactivity and excellent release efficiencies of payloads from ICPr/ICPrc caging groups in both neutral and acidic buffer solutions, as determined by HPLC analyses. The dual‐release mechanism was successfully extended to living systems. LSCM studies unveiled intense intracellular emission following incubation with complex **3a** and ICPr‐fluorescein in HeLa cells, indicating the effective release of the emissive complex **3b** and the unbound fluorophore. Additionally, the synergistic interaction between complex‐induced ^1^O_2_ photosensitization and prodrug activation facilitated by this bioorthogonal platform, as demonstrated using complex **3a** with ICPrc‐Dox, seamlessly integrates photodynamic therapy with chemotherapy while maintaining precise spatiotemporal control. A key aspect of this strategy is the targeted localization of the released 3‐hydroxypyridine complexes in the lysosomes, coupled with enhanced ^1^O_2_ photosensitization under the acidic conditions of these organelles. This enhances the therapeutic efficacy within the acidic TME, underscoring the potential of this approach to effectively inhibit the growth and proliferation of solid tumors. To conclude, the integration of bioorthogonal dissociation reactions with targeted prodrug activation presents a promising avenue for advancing cancer treatment. By leveraging the unique properties of rhenium(I) TzMe complexes, this strategy offers a powerful tool for achieving precise therapeutic outcomes, paving the way for more effective and targeted anticancer therapies.

## Conflict of Interests

The authors declare no conflict of interest.

## Supporting information



Supporting Information

## Data Availability

The data that support the findings of this study are available in the Supporting Information of this article.

## References

[1] P. Mi , Theranostics 2020, 10, 4557–4588, 10.7150/thno.38069. 32292515 PMC7150471

[2] F. M. Kashkooli , M. Soltani , M. Souri , J. Controlled Release 2020, 327, 316–349, 10.1016/j.jconrel.2020.08.012. 32800878

[3] F. Kratz , I. A. Müller , C. Ryppa , A. Warnecke , ChemMedChem 2008, 3, 20–53, 10.1002/cmdc.200700159. 17963208

[4] L. Bildstein , C. Dubernet , P. Couvreur , Adv. Drug Delivery Rev. 2011, 63, 3–23, 10.1016/j.addr.2010.12.005. 21237228

[5] C. Ding , C. Chen , X. Zeng , H. Chen , Y. Zhao , ACS Nano 2022, 16, 13513–13553, 10.1021/acsnano.2c05379. 36048467

[6] H.‐H. Han , H.‐M. Wang , P. Jangili , M. Li , L. Wu , Y. Zang , A. C. Sedgwick , J. Li , X.‐P. He , T. D. James , J. S. Kim , Chem. Soc. Rev. 2023, 52, 879–920, 10.1039/D2CS00673A. 36637396

[7] Q. Fu , S. Zhang , S. Shen , Z. Gu , J. Chen , D. Song , P. Sun , C. Wang , Z. Guo , Y. Xiao , Y. Q. Gao , Z. Guo , Z. Liu , Nat. Biomed. Eng. 2024, 8, 1425–1435, 10.1038/s41551-024-01239-x. 39025943

[8] C. Wang , Z. Zhang , Z. Liu , ACS Cent. Sci. 2025, 11, 1306–1320, 10.1021/acscentsci.5c00875. 40893960 PMC12395309

[9] G. Liu , Y. Zhang , H. Yao , Z. Deng , S. Chen , Y. Wang , W. Peng , G. Sun , M.‐K. Tse , X. Chen , J. Yue , Y.‐K. Peng , L. Wang , G. Zhu , Sci. Adv. 2023, 9, eadg5964, 10.1126/sciadv.adg5964. 37343091 PMC10284555

[10] S. N. Ekdawi , D. A. Jaffray , C. Allen , Nano Today 2016, 11, 402–414, 10.1016/j.nantod.2016.06.006.

[11] Q. Fu , S. Shen , P. Sun , Z. Gu , Y. Bai , X. Wang , Z. Liu , Chem. Soc. Rev. 2023, 52, 7737–7772, 10.1039/D2CS00889K. 37905601

[12] J. Wang , X. Wang , X. Fan , P. R. Chen , ACS Cent. Sci. 2021, 7, 929–943, 10.1021/acscentsci.1c00124. 34235254 PMC8227596

[13] Q. Yao , F. Lin , X. Fan , Y. Wang , Y. Liu , Z. Liu , X. Jiang , P. R. Chen , Y. Gao , Nat. Commun. 2018, 9, 5032, 10.1038/s41467-018-07490-6. 30487642 PMC6261997

[14] T. Deb , R. M. Franzini , Synlett 2020, 31, 938–944, 10.1055/s-0039-1690849.

[15] J. Tu , M. Xu , S. Parvez , R. T. Peterson , R. M. Franzini , J. Am. Chem. Soc. 2018, 140, 8410–8414, 10.1021/jacs.8b05093. 29927585

[16] J. Xiong , E. Y. Xue , Q. Wu , P.‐C. Lo , D. K. P. Ng , J. Controlled Release 2023, 353, 663–674, 10.1016/j.jconrel.2022.12.015. 36503072

[17] J. Tu , D. Svatunek , S. Parvez , H. J. Eckvahl , M. Xu , R. T. Peterson , K. N. Houk , R. M. Franzini , Chem. Sci. 2020, 11, 169–179, 10.1039/C9SC04649F. 32110368 PMC7012038

[18] X. Zhang , H. Xu , J. Li , D. Su , W. Mao , G. Shen , L. Li , H. Wu , Chem. Commun. 2022, 58, 573–576, 10.1039/D1CC05774J. 34913446

[19] J. Shum , L. C.‐C. Lee , M. W.‐L. Chiang , Y.‐W. Lam , K. K.‐W. Lo , Angew. Chem. Int. Ed. 2023, 62, e202303931, 10.1002/anie.202303931. 37191224

[20] A. M.‐H. Yip , C. K.‐H. Lai , K. S.‐M. Yiu , K. K.‐W. Lo , Angew. Chem. Int. Ed. 2022, 61, e202116078, 10.1002/anie.202116078. 35119163

[21] P. K.‐K. Leung , L. C.‐C. Lee , H. H.‐Y. Yeung , K.‐W. Io , K. K.‐W. Lo , Chem. Commun. 2021, 57, 4914–4917, 10.1039/D1CC00545F. 33870960

[22] A. W.‐T. Choi , K. K.‐S. Tso , V. M.‐W. Yim , H.‐W. Liu , K. K.‐W. Lo , Chem. Commun. 2015, 51, 3442–3445, 10.1039/C4CC09532D. 25627806

[23] T. S.‐M. Tang , H.‐W. Liu , K. K.‐W. Lo , Chem. Commun. 2017, 53, 3299–3302, 10.1039/C7CC00427C. 28256651

[24] S. P.‐Y. Li , A. M.‐H. Yip , H.‐W. Liu , K. K.‐W. Lo , Biomaterials 2016, 103, 305–313, 10.1016/j.biomaterials.2016.06.065. 27429251

[25] G.‐X. Xu , L. C.‐C. Lee , P. K.‐K. Leung , E. C.‐L. Mak , J. Shum , K. Y. Zhang , Q. Zhao , K. K.‐W. Lo , Chem. Sci. 2023, 14, 13508–13517, 10.1039/D3SC04903E. 38033895 PMC10686031

[26] P. K.‐K. Leung , L. C.‐C. Lee , T. K.‐Y. Ip , H.‐W. Liu , S.‐M. Yiu , N. P. Lee , K. K.‐W. Lo , Chem. Commun. 2021, 57, 11256–11259, 10.1039/D1CC04740J. 34633395

[27] A. W.‐T. Choi , M.‐W. Louie , S. P.‐Y. Li , H.‐W. Liu , B. T.‐N. Chan , T. C.‐Y. Lam , A. C.‐C. Lin , S.‐H. Cheng , K. K.‐W. Lo , Inorg. Chem. 2012, 51, 13289–13302, 10.1021/ic301948d. 23198846

[28] L. Wallace , D. P. Rillema , Inorg. Chem. 1993, 32, 3836–3843, 10.1021/ic00070a012.

[29] G.‐X. Xu , L. C.‐C. Lee , C. W.‐C. Kwok , P. K.‐K. Leung , J.‐H. Zhu , K. K.‐W. Lo , Eur. J. Inorg. Chem. 2021, 2021, 3432–3442, 10.1002/ejic.202100364.

[30] W. D. Bare , N. H. Mack , J. N. Demas , B. A. DeGraff , Proc. SPIE 2001, 4199, 1–7, 10.1117/12.417357.

[31] W. D. Bare , N. H. Mack , J. N. Demas , B. A. DeGraff , Appl. Spectrosc. 2004, 58, 1093–1100, 10.1366/0003702041959316. 15479526

[32] M. Licini , J. A. G. Williams , Chem. Commun. 1999, 1943–1944, 10.1039/a906203c.

[33] E. Boedtkjer , S. F. Pedersen , Annu. Rev. Physiol. 2020, 82, 103–126, 10.1146/annurev-physiol-021119-034627. 31730395

[34] R. Bonnett , Chem. Soc. Rev. 1995, 24, 19–33, 10.1039/cs9952400019.

[35] M. C. DeRosa , R. J. Crutchley , Coord. Chem. Rev. 2002, 233–234, 351–371, 10.1016/S0010-8545(02)00034-6.

[36] L. He , Y. Li , C.‐P. Tan , R.‐R. Ye , M.‐H. Chen , J.‐J. Cao , L.‐N. Ji , Z.‐W. Mao , Chem. Sci. 2015, 6, 5409–5418, 10.1039/C5SC01955A. 29861886 PMC5947539

[37] S. Mukherjee , R. N. Ghosh , F. R. Maxfield , Physiol. Rev. 1997, 77, 759–803, 10.1152/physrev.1997.77.3.759. 9234965

[38] J. A. Mindell , Annu. Rev. Physiol. 2012, 74, 69–86, 10.1146/annurev-physiol-012110-142317. 22335796

[39] A. J. M. van Beijnen , R. J. M. Nolte , A. J. Naaktgeboren , J. W. Zwikker , W. Drenth , A. M. F. Hezemans , Macromolecules 1983, 16, 1679–1689, 10.1021/ma00245a001.

[40] A. M. van Leusen , B. E. Hoogenboom , H. Siderius , Tetrahedron Lett. 1972, 13, 2369–2372, 10.1016/S0040-4039(01)85305-3.

[41] E. C.‐L. Mak , Z. Chen , L. C.‐C. Lee , L.‐L. Yan , V. W.‐W. Yam , K. K.‐W. Lo , JACS Au 2025, 5, 2825–2836, 10.1021/jacsau.5c00413. 40575299 PMC12188478

[42] C. A. Puckett , R. J. Ernst , J. K. Barton , Dalton Trans. 2010, 39, 1159–1170, 10.1039/B922209J. 20104335 PMC2873847

[43] J. Lin , K. Yang , E. J. New , Org. Biomol. Chem. 2021, 19, 9339–9357, 10.1039/D1OB01447A. 34515288

[44] J. B. Lloyd , Adv. Drug Delivery Rev. 2000, 41, 189–200, 10.1016/S0169-409X(99)00065-4. 10699314

[45] W. Xu , Z. Zeng , J.‐H. Jiang , Y.‐T. Chang , L. Yuan , Angew. Chem. Int. Ed. 2016, 55, 13658–13699, 10.1002/anie.201510721. 27571316

