# Supplementary material for: Controlled Dual Activation of Rhenium(I) Photosensitizers and Profluorophores/Prodrugs via a Dissociative Bioorthogonal Tetrazine–Isonitrile Reaction
Source: Angew Chem Int Ed Engl. 2025 Oct 21;64(50):e202516957. doi: 10.1002/anie.202516957 (PMC12684313; doi:10.1002/anie.202516957)
Supplement: Supplementary file 1 — Supporting Information [file ANIE-64-e202516957-s001.pdf]

Supporting Information

Controlled Dual Activation of Rhenium(I) Photosensitizers and Profluorophores/Prodrugs via a Dissociative Bioorthogonal Tetrazine–Isonitrile Reaction

Eunice Chiu-Lam Mak,[a] Lawrence Cho-Cheung Lee,[a] and Kenneth Kam-Wing Lo*[a,b]

[a] Department of Chemistry, City University of Hong Kong, Kowloon, Hong Kong, P. R. China

[b] State Key Laboratory of Terahertz and Millimetre Waves, City University of Hong Kong, Kowloon, Hong Kong, P. R. China

Table of Contents

**Experimental Section**		S10
**Table S1**	Electronic absorption spectral data of the rhenium(I) complexes and ligands py-OCH_2-Tz-^{t}Bu and py-OH at 298 K.	S30
**Table S2**	Photophysical data of the rhenium(I) complexes.	S32
**Table S3**	Emission wavelengths (λ_{em}), emission lifetimes (τ), and emission enhancement factors (I/I_o) of complexes **1a** – **3a** (10 μM) upon incubation without or with ICPrc-Bn (500 μM) in aerated H_2O/DMSO (4:1, *v*/*v*) at 37°C for 18 h.	S34
**Table S4**	Emission wavelengths (λ_{em}), emission lifetimes (τ), and emission enhancement factors (I/I_o) of complexes **1a** – **3a** (10 μM) upon incubation without or with ICPrc-Bn (500 μM) in aerated McIlvaine buffer (pH 5.0)/DMSO (4:1, *v*/*v*) at 37°C for 18 h.	S35
**Table S5**	Emission wavelengths (λ_{em}), emission lifetimes (τ), and emission enhancement factors (I/I_o) of complexes **1a** – **3a** (10 μM) upon incubation without or with ICPrc-Bn (500 μM) in aerated McIlvaine buffer (pH 7.4)/DMSO (4:1, *v*/*v*) at 37°C for 18 h.	S36
**Table S6**	$^1O_2$ generation quantum yields (Φ_Δ) of the rhenium(I) complexes in aerated CH_3CN at 298 K.	S37
**Table S7**	$^1O_2$ generation quantum yields (Φ_Δ) of complexes **3a** and **3b** in aerated McIlvaine buffer (pH 3.0, 5.0, and 7.4)/CH_3CN (4:1, *v*/*v*) at 298 K using ABDA as the $^1O_2$ indicator.	S38

**Table S8**	Flow cytometric results of HeLa cells incubated with complexes **1a** – **3a** (10 μM) for 3 h, followed by incubation with ICPrc-Bn (200 μM) or fresh medium for 4 h.	S39
**Table S9**	Cellular uptake of complexes **1a** – **3a** in HeLa and HEK-293 cells.	S40
**Table S10**	(Photo)cytotoxicity of complex **3b** toward HeLa cells without or with ICPrc-Dox post-treatment in the dark and upon irradiation at 365 nm (5 mW cm^{-2}) for 5 min.[a] Photocytotoxicity index (PI) is the ratio $IC_{50,dark}/IC_{50,light}$.	S41
**Scheme S1**	Synthesis of the ligand py-OCH$_2$-Tz-^{t}Bu.	S42
**Figure S1**	Electronic absorption spectra of the rhenium(I) complexes and ligands py-OCH$_2$-Tz-^{t}Bu and py-OH in CH_2Cl_2 (black) and CH_3CN (red) at 298 K.	S43
**Figure S2**	Emission spectra of the rhenium(I) complexes in CH_2Cl_2 (black) and CH_3CN (red) at 298 K and alcohol glass at 77 K (blue).	S44
**Figure S3**	ESI mass spectra of the reaction mixture of complexes **1a** – **3a** (10 μM) with ICPrc-Bn (500 μM) in aerated H_2O/DMSO (4:1, *v*/*v*) after incubation at 37°C for 18 h.	S45
**Figure S4**	Emission spectra of complexes **1a** – **3a** (10 μM) before (black) and after (red) incubation with ICPrc-Bn (500 μM) in aerated McIlvaine buffer (pH 7.4)/DMSO (4:1, *v*/*v*) at 37°C for 18 h.	S46
**Figure S5**	ESI mass spectrum of the reaction mixture of complex **3a** (10 μM) with BCN-OH (500 μM) in aerated H_2O/DMSO (4:1, *v*/*v*) after incubation at 37°C for 18 h.	S47

Figure S6 Rate of decay in the absorbance of ABDA (100 μM) at ≈ 378 nm in aerated McIlvaine buffer (pH 3.0, 5.0, and 7.4)/CH_3CN (4:1, *v*/*v*) in the presence of complex **3a** or **3b** upon irradiation at 355 nm. A solution of ABDA without any sensitizers was used as the negative control. S48

Figure S7 HPLC chromatograms of the reaction mixtures of complexes **1a** – **3a** (20 μM) without (black) or with ICPr-coum (25 μM) (red) in aerated (a) McIlvaine buffer (pH 7.4)/DMSO (4:1, *v*/*v*) and (b) McIlvaine buffer (pH 5.0)/DMSO (4:1, *v*/*v*) at 37°C for 18 h. The absorbance was monitored at 325 nm. S49

Figure S8 Second-order kinetics for the reaction of complexes **1a** – **3a** (20 μM) with ICPr-coum (25 μM) at different time points in aerated (a) McIlvaine buffer (pH 7.4)/DMSO (4:1, *v*/*v*) and (b) McIlvaine buffer (pH 5.0)/DMSO (4:1, *v*/*v*) after incubation at 37°C. The slope of the linear fit corresponds to the k_2 value of the reaction. S50

Figure S9 HPLC chromatograms of the reaction mixtures of complexes **1a** – **3a** (50 μM) without (black) or with ICPr-coum (20 μM) (red) in aerated (a) McIlvaine buffer (pH 7.4)/DMSO (4:1, *v*/*v*) and (b) McIlvaine buffer (pH 5.0)/DMSO (4:1, *v*/*v*) at 37°C for 18 h. The absorbance was monitored at 325 nm. S51

Figure S10 Release profiles of umbelliferone from ICPr-coum (20 μM) after incubation with complexes **1a** – **3a** (50 μM) in aerated (a) McIlvaine buffer (pH 7.4)/DMSO (4:1, *v*/*v*) and (b) McIlvaine buffer (pH 5.0)/DMSO (4:1, *v*/*v*) at 37°C. S52

Figure S11 HPLC chromatograms of the reaction mixtures of the ligand py-OCH_2-Tz-^{t}Bu (200 μM) without (black) or with ICPr-coum (250 μM) (red) in aerated (a) McIlvaine buffer (pH 7.4)/CH_3CN (4:1, *v*/*v*) and (b) McIlvaine buffer (pH 5.0)/CH_3CN (4:1, *v*/*v*) at 37°C for 18 h. The absorbance was monitored at 270 and 325 nm. S53

Figure S12 Second-order kinetics for the reaction of the ligand py-OCH_2-Tz-^{t}Bu (200 μM) with ICPr-coum (250 μM) at different time points in aerated (a) McIlvaine buffer (pH 7.4)/CH_3CN (4:1, *v*/*v*) and (b) McIlvaine buffer (pH 5.0)/CH_3CN (4:1, *v*/*v*) after incubation at 37°C. The slope of the linear fit corresponds to the k_2 value of the reaction. S54

Figure S13 HPLC chromatograms of the reaction mixtures of the ligand py-OCH_2-Tz-^{t}Bu (200 μM) without (black) or with ICPr-coum (80 μM) (red) in aerated (a) McIlvaine buffer (pH 7.4)/CH_3CN (4:1, *v*/*v*) and (b) McIlvaine buffer (pH 5.0)/CH_3CN (4:1, *v*/*v*) at 37°C for 18 h. The absorbance was monitored at 270 and 325 nm. S55

Figure S14 Release profiles of umbelliferone from ICPr-coum (80 μM) after incubation with the ligand py-OCH_2-Tz-^{t}Bu (200 μM) in aerated (a) McIlvaine buffer (pH 7.4)/CH_3CN (4:1, *v*/*v*) and (b) McIlvaine buffer (pH 5.0)/CH_3CN (4:1, *v*/*v*) at 37°C. S56

Figure S15 ESI mass spectra of a CH_2Cl_2 extract of lysed HeLa cells that were incubated with complex **3a** (10 μM, 3 h), followed by (a) ICPrc-Bn (200 μM, 4 h) or (b) fresh DMEM (4 h) at 37°C. S57

Figure S16 Flow cytometric results of HeLa cells incubated with (a) complex **1a**, (b) complex **2a**, and (c) complex **3a** under the following conditions: (i) S58

blank medium for 4 h (red), (ii) the complex (10 μM) for 3 h, followed by fresh medium for 4 h (green), and (iii) the complex (10 μM) for 3 h, followed by ICPrc-Bn (200 μM) for 4 h (magenta) at 37°C.

Figure S17 LSCM images of HeLa cells incubated with complexes **1a** – **3a** (10 μM, 3 h, λ_{ex} = 405 nm, λ_{em} = 500 – 600 nm for complexes **1a** and **2a** and 550 − 600 nm for complex **3a**) and ICPrc-Bn (200 μM, 4 h), and further incubated with MitoTracker Deep Red (100 nM, 20 min, λ_{ex} = 635 nm, λ_{em} = 650 − 670 nm) at 37°C. PCC = 0.21 (complex **1a**), 0.17 (complex **2a**), and 0.56 (complex **3a**). Scale bar = 25 μm. S59

Figure S18 Relative amounts of rhenium associated with an average HeLa cell upon incubation with complex **3a** (10 μM, 1 h) at 37°C without or with preincubation of the cells at 4°C for 1 h, EIPA (50 μM, 1.5 h), Me-β-CD (5 mM, 1 h), or chlorpromazine (30 μM, 1 h) at 37°C. The uptake values at 37°C without pretreatment were taken as the reference. S60

Figure S19 LSCM images of HeLa cells incubated with ICPr-coum (200 μM, 4 h, λ_{ex} = 405 nm, λ_{em} = 430 – 500 nm) alone, or incubated with complex **3a** (10 μM, 3 h, λ_{ex} = 405 nm, λ_{em} = 550 – 600 nm), followed by incubation without or with ICPr-coum (10 μM, 4 h), or incubation with ICPrc-Bn (200 μM, 4 h) at 37°C. PCC = 0.58 (complex **3a** + ICPr-coum). Scale bar = 25 μm. S61

Figure S20 HPLC analysis of the stability of (a) ICPr-fluorescein (25 μM) and (b) ICPrc-Dox (25 μM) in 25% FBS at 37°C in the dark for 48 h. The S62

absorbance was monitored at 250 nm for ICPr-fluorescein and at 480 nm for ICPrc-Dox.

Figure S21 HPLC chromatograms of (a) fluorescein (10 μM) (black) and ICPr-fluorescein (10 μM) (red), and (b) the reaction mixture of complex **3a** (10 μM) without (black) or with ICPr-fluorescein (10 μM) (red) in aerated PBS/DMSO (4:1, *v*/*v*) at 37°C for 4 h and (c) 24 h. The absorbance was monitored at 250 and 325 nm. S63

Figure S22 HPLC chromatograms of (a) Dox (10 μM) (black) and ICPrc-Dox (10 μM) (red), and (b) the reaction mixture of complex **3a** (10 μM) without (black) or with ICPrc-Dox (10 μM) (red) in aerated PBS/DMSO (4:1, *v*/*v*) at 37°C for 4 h and (c) 24 h. The absorbance was monitored at 325 and 480 nm. S64

Figure S23 HPLC traces of the reaction mixtures of complex **3a** (50 μM) with (a) ICPr-coum (50 μM), (b) ICPr-fluorescein (50 μM), and (c) ICPrc-Dox (μM) in aerated McIlvaine buffer (pH 7.4)/DMSO (4:1, *v*/*v*) at 37°C, recorded at various time points. The absorbance was monitored at 325 nm for ICPr-coum and at 250 nm for ICPr-fluorescein and ICPrc-Dox. (d) Release profiles of umbelliferone, fluorescein, and Dox from ICPr/ICPrc-caged compounds (50 μM) after incubation with complex **3a** (50 μM) under the same conditions. S65

Figure S24 Viability of HEK-293 cells incubated with complex **3a** for 3 h, and then incubated (a) with fresh growth medium, (b) ICPrc-Dox (10 μM), or (c) ICPrc-Dox (50 μM) for 4 h. HeLa cells incubated with (d) Dox for 4 h served as a positive control. The cells were further incubated in the dark S66

	(blue) or irradiated at 365 nm (5 mW cm^{-2}) (red) for 5 min, and then incubated with fresh growth medium for 24 h.	
**Figure S25**	^{1}H NMR spectrum of py-OCH$_2$-Tz-^{t}Bu in CDCl$_3$ at 298 K.	S67
**Figure S26**	^{1}H NMR spectrum of complex **1a** in CDCl$_3$ at 298 K.	S68
**Figure S27**	^{13}C NMR spectrum of complex **1a** in CDCl$_3$ at 298 K.	S69
**Figure S28**	^{1}H NMR spectrum of complex **1b** in (CD$_3$)$_2$CO at 298 K.	S70
**Figure S29**	^{13}C NMR spectrum of complex **1b** in (CD$_3$)$_2$CO at 298 K.	S71
**Figure S30**	^{1}H NMR spectrum of complex **2a** in CDCl$_3$ at 298 K.	S72
**Figure S31**	^{13}C NMR spectrum of complex **2a** in CDCl$_3$ at 298 K.	S73
**Figure S32**	^{1}H NMR spectrum of complex **2b** in (CD$_3$)$_2$CO at 298 K.	S74
**Figure S33**	^{13}C NMR spectrum of complex **2b** in (CD$_3$)$_2$CO at 298 K.	S75
**Figure S34**	^{1}H NMR spectrum of complex **3a** in CDCl$_3$ at 298 K.	S76
**Figure S35**	^{13}C NMR spectrum of complex **3a** in CDCl$_3$ at 298 K.	S77
**Figure S36**	^{1}H NMR spectrum of complex **3b** in (CD$_3$)$_2$CO at 298 K.	S78
**Figure S37**	^{13}C NMR spectrum of complex **3b** in (CD$_3$)$_2$CO at 298 K.	S79
**Figure S38**	^{1}H NMR spectrum of ICPr-fluorescein in CDCl$_3$ at 298 K.	S80
**Figure S39**	(a) Experimental and (b) simulated HR-ESI mass spectra of complex **1a** in CH$_3$CN.	S81
**Figure S40**	(a) Experimental and (b) simulated HR-ESI mass spectra of complex **1b** in CH$_3$CN.	S82
**Figure S41**	(a) Experimental and (b) simulated HR-ESI mass spectra of complex **2a** in CH$_3$CN.	S83
**Figure S42**	(a) Experimental and (b) simulated HR-ESI mass spectra of complex **2b** in CH$_3$CN.	S84

Figure S43 (a) Experimental and (b) simulated HR-ESI mass spectra of complex **3a** in CH_3CN. S85

Figure S44 (a) Experimental and (b) simulated HR-ESI mass spectra of complex **3b** in CH_3CN. S86

References S87

Experimental Section

Materials and Synthesis

All solvents were of analytical reagent grade and purified according to standard procedures.[1] 3-Hydroxypyridine, K_2CO_3, anhydrous $MgSO_4$, triethylamine, hydrazine monohydrate, $Re_2(CO)_{10}$, and cisplatin were purchased from Acros. Sulfur, (1*R*,8*S*,9*s*)-bicyclo[6.1.0]non-4-yn-9-ylmethanol (BCN-OH), and methyl-β-cyclodextrin (Me-β-CD) were sourced from Aldrich. HCl was sourced from Honeywell Fluka. $NaNO_2$, bromoacetonitrile, trimethylacetonitrile, and doxorubicin (Dox) hydrochloride were sourced from Energy Chemical. 3-(4,5-Dimethylthiazol-2-yl)-2,5-diphenyltetrazolium bromide (MTT) and fluorescein were purchased from Macklin. Cs_2CO_3 and 5-(*N*-ethyl-*N*-isopropyl)amiloride (EIPA) were sourced from J&K Scientific. 9,10-Anthracenediyl-*bis*(methylene)dimalonic acid (ABDA) was purchased from Abcam. Chlorpromazine hydrochloride was sourced from Tokyo Chemical Industry. All these chemicals were used without further purification. $[Re(N^\wedge N)(CO)_3(CH_3CN)](CF_3SO_3)$ was prepared by modification of a previously reported method.[2] 3-Isocyanopropyl(pentafluorophenyl) carbonate (ICPr-PFC), 3-isocyanopropyl benzylcarbamate (ICPrc-Bn),[3] 3-isocyano-1-tosylpropane (ICPr-tos),[4] 7-(3-isocyanopropyl-1-oxy)coumarin (ICPr-coum)[4], and *N*-(3-isocyanopropyl-1-carbamoyl)doxorubicin (ICPrc-Dox)[3] were synthesized as described previously. All buffer components were of biological grade and used as received. Autoclaved Milli-Q H_2O was used for the preparation of the aqueous solutions. Dulbecco's modified Eagle's medium (DMEM), fetal bovine serum (FBS), phosphate-buffered saline (PBS) at pH 7.4, trypsin-EDTA, penicillin/streptomycin, MitoTracker Deep Red, and LysoTracker Deep Red were purchased from Invitrogen. The growth medium for cell culture contained DMEM with 10% FBS and 1% penicillin/streptomycin. HeLa and HEK-293 cells were obtained from American Type Culture Collection.

3-(*tert*-Butyl)-6-((pyridin-3-yloxy)methyl)-1,2,4,5-tetrazine (py-OCH_2-Tz-^{t}Bu)

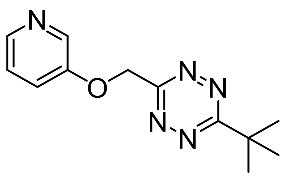

A mixture of 3-hydroxypyridine (2.0 g, 21.03 mmol), bromoacetonitrile (1.22 mL, 17.53 mmol), and K_2CO_3 (2.6 g, 18.81 mmol) in $(CH_3)_2CO$ (30 mL) was stirred at 298 K for 18 h. The reaction mixture was filtered and evaporated to dryness. The dark brown crude product was purified by column chromatography on silica gel and eluted with *n*-hexane/ethyl acetate (1:1, *v*/*v*). The solvent was removed under vacuum to afford the product as a yellow oil. The resultant 3-(cyanomethoxy)pyridine was used in the next step without further purification. A mixture of 3-(cyanomethoxy)pyridine (400 mg, 2.96 mmol), trimethylacetonitrile (1.64 mL, 14.81 mmol), sulfur (100 mg, 3.13 mmol), and hydrazine monohydrate (2.87 mL, 59.20 mmol) in EtOH (10 mL) was heated in an oil bath at 70°C under an inert atmosphere of N_2 for 24 h. After the mixture was cooled to room temperature, $NaNO_2$ (4.1 g, 59.20 mmol) in H_2O (5 mL) was added, followed by the addition of 1 M HCl until pH = 3. The aqueous phase was extracted with CH_2Cl_2 (100 mL × 3). The combined organic extract was dried over anhydrous $MgSO_4$, filtered, and the solvent was removed under reduced pressure. The purple solid residue was purified by column chromatography on silica gel using *n*-hexane/ethyl acetate (3:1, *v*/*v*) as the eluent. The solvent was removed under reduced pressure to give a purple oil. Yield: 145 mg (20%). ^{1}H NMR (300 MHz, $CDCl_3$, 298 K): δ 8.53 (d, *J* = 3.0 Hz, 1H, H2 of py), 8.37 (dd, *J* = 5.0, 1.1 Hz, 1H, H6 of py), 7.70 (ddd, *J* = 8.6, 3.0, 1.1 Hz, 1H, H5 of py), 7.51 (dd, *J* = 8.6, 5.0 Hz, 1H, H4 of py), 5.78 (s, 2H, CH_2), 1.61 (s, 9H, $(CH_3)_3$). MS (ESI, positive mode, *m*/*z*): 246.3 $[M + H]^+$.

[Re(Me_2-bpy)($CO)_3$(py-OCH_2-Tz-^{t}Bu)](CF_3SO_3) (**1a**)

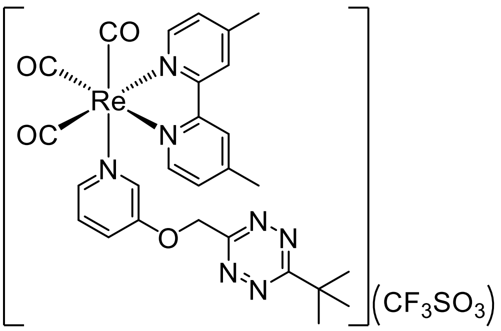

A mixture of [Re(Me_2-bpy)($CO)_3$(CH_3CN)](CF_3SO_3) (50 mg, 0.078 mmol) and py-OCH_2-Tz-^{t}Bu (19 mg, 0.078 mmol) in THF (12 mL) was refluxed under an inert atmosphere of N_2 in the dark for 12 h. The solvent was removed under reduced pressure, and the red-purple solid was purified by column chromatography on silica gel using CH_2Cl_2/$(CH_3)_2CO$ (2:1, *v*/*v*) as the eluent. The solvent was removed under reduced pressure to give a dark pink solid. Subsequent recrystallization of the solid from CH_2Cl_2/Et_2O afforded the complex as dark pink crystals. Yield: 43 mg (65%). ^{1}H NMR (300 MHz, $CDCl_3$, 298 K): δ 8.86 (d, *J* = 5.6 Hz, 2H, H6 and H6' of bpy), 8.68 (s, 2H, H3 and H3' of bpy), 8.08 (d, *J* = 2.6 Hz, 1H, H2 of py), 7.70 (d, *J* = 5.3 Hz, 1H, H6 of py), 7.59 (dd, *J* = 8.1, 2.1 Hz, 1H, H5 of py), 7.49 (d, *J* = 4.8 Hz, 2H, H5 and H5' of bpy), 7.32 (dd, 1H, *J* = 8.4, 5.4 Hz, H4 of py), 5.60 (s, 2H, CH_2), 2.66 (s, 6H, CH_3 at C4 and C4' of bpy), 1.60 (s, 9H, $(CH_3)_3$). ^{13}C NMR (150 MHz, $CDCl_3$, 298 K): δ 177.5, 164.1, 156.4, 155.8, 155.1, 151.7, 144.5, 141.4, 129.5, 127.7, 127.2, 125.2, 119.8. IR (KBr) $\tilde{\nu}$/cm^{-1}: 2031 (s, C≡O), 1923 (s, C≡O), 1156 (m, $CF_3SO_3^-$), 1034 (m, $CF_3SO_3^-$). HR-MS (ESI, positive mode, *m*/*z*): [M – $CF_3SO_3^-$]$^+$ calcd for $ReC_{27}H_{27}N_7O_4$ 700.1679, found 700.1667.

[Re(Me_2-bpy)(CO)$_3$(py-OH)](CF_3SO_3) (**1b**)

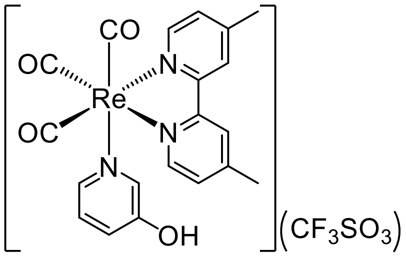

The synthetic procedure was similar to that for the preparation of complex **1a**, except that 3-hydroxypyridine (7.4 mg, 0.078 mmol) was used instead of py-OCH_2-Tz-^{t}Bu. Subsequent recrystallization of the solid from CH_2Cl_2/Et_2O afforded the complex as dark pink crystals. Yield: 37 mg (68%). ^{1}H NMR (400 MHz, $(CD_3)_2CO$, 298 K): δ 9.66 (br, 1H, OH), 9.28 (d, *J* = 5.7 Hz, 2H, H6 and H6' of bpy), 8.64 (s, 2H, H3 and H3' of bpy), 8.06 (dd, *J* = 5.4, 0.6 Hz, 1H, H6 of py), 8.02 (d, *J* = 2.6 Hz, 1H, H2 of py), 7.84 (dd, *J* = 5.7, 1.0 Hz, 2H, H5 and H5' of bpy), 7.47 – 7.42 (m, 1H, H5 of py), 7.31 (dd, *J* = 8.4, 5.4 Hz, 1H, H4 of py), 2.65 (s, 6H, CH_3 at C4 and C4' of bpy). ^{13}C NMR (150 MHz, $CDCl_3$, 298 K): δ 155.9, 155.6, 154.2, 153.2, 143.6, 140.0, 129.7, 127.2, 126.3, 125.5, 120.3. IR (KBr) $\tilde{\nu}$/cm^{-1}: 2032 (s, C≡O), 1925 (s, C≡O), 1158 (m, $CF_3SO_3^-$), 1032 (m, $CF_3SO_3^-$). HR-MS (ESI, positive mode, *m*/*z*): [M – $CF_3SO_3^-$]$^+$ calcd for $ReC_{20}H_{17}N_3O_4$ 550.0785, found 550.0764.

[Re(phen)(CO)$_3$(py-OCH$_2$-Tz-^{t}Bu)](CF$_3$SO$_3$) (**2a**)

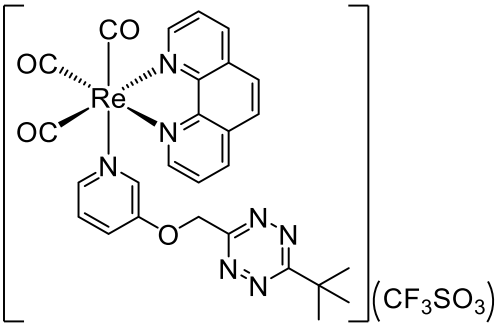

The synthetic procedure was similar to that for the preparation of complex **1a**, except that [Re(phen)(CO)$_3$(CH$_3$CN)](CF$_3$SO$_3$) (50 mg, 0.078 mmol) was used instead of [Re(Me$_2$-bpy)(CO)$_3$(CH$_3$CN)](CF$_3$SO$_3$). Subsequent recrystallization of the solid from CH$_2$Cl$_2$/Et$_2$O afforded the complex as dark pink crystals. Yield: 40 mg (60%). ^{1}H NMR (300 MHz, CDCl$_3$, 298 K): δ 9.59 (dd, J = 5.2, 1.1 Hz, 2H, H2 and H9 of phen), 8.86 (dd, J = 8.3, 1.1 Hz, 2H, H4 and H7 of phen), 8.24 – 8.15 (m, 5H, H3, H5, H6, and H8 of phen and H2 of py), 7.89 (d, J = 5.3 Hz, 1H, H6 of py), 7.49 (dd, J = 8.7, 2.1 Hz, 1H, H5 of py), 7.31 (dd, J = 8.7, 5.5 Hz, 1H, H4 of py), 5.55 (s, 2H, CH$_2$), 1.59 (s, 9H, (CH$_3$)$_3$). ^{13}C NMR (150 MHz, CDCl$_3$, 298 K): δ 177.17, 163.98, 156.16, 153.77, 146.49, 145.05, 140.70, 140.56, 131.43, 128.70, 127.85, 127.56, 125.52, 121.81, 119.69, 68.51, 65.86, 38.30, 29.12, 15.28. IR (KBr) $\tilde{\nu}$/cm^{-1}: 2038 (s, C≡O), 1918 (s, C≡O), 1158 (m, CF$_3$SO$_3^-$), 1034 (m, CF$_3$SO$_3^-$). HR-MS (ESI, positive mode, *m*/*z*): [M – CF$_3$SO$_3^-$]$^+$ calcd for ReC$_{27}$H$_{23}$N$_7$O$_4$ 696.1378, found 696.1349.

[Re(phen)(CO)$_3$(py-OH)](CF$_3$SO$_3$) (**2b**)

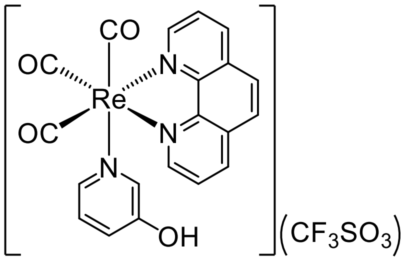

The synthetic procedure was similar to that for the preparation of complex **1b**, except that [Re(phen)(CO)$_3$(CH$_3$CN)](CF$_3$SO$_3$) (50 mg, 0.078 mmol) was used instead of [Re(Me$_2$-bpy)(CO)$_3$(CH$_3$CN)](CF$_3$SO$_3$). Subsequent recrystallization of the solid from CH$_2$Cl$_2$/Et$_2$O afforded the complex as dark pink crystals. Yield: 35 mg (65%). ^{1}H NMR (300 MHz, (CD$_3$)$_2$CO, 298 K): δ 9.80 (d, J = 5.0 Hz, 2H, H2 and H9 of phen), 9.04 (d, J = 8.3 Hz, 2H, H4 and H7 of phen), 8.35 – 8.25 (m, 4H, H3, H5, H6, and H8 of phen), 7.87 (d, J = 4.5 Hz, 1H, H6 of py), 7.78 (s, 1H, H2 of py), 7.11 – 7.00 (m, 2H, H4 and H5 of py). ^{13}C NMR (150 MHz, (CD$_3$)$_2$CO, 298 K): δ 154.59, 146.51, 140.66, 140.48, 140.42, 131.27, 128.29, 127.41, 126.80, 126.50, 54.06, 14.70. IR (KBr) $\tilde{\nu}$/cm^{-1}: 2031 (s, C≡O), 1923 (s, C≡O), 1168 (m, CF$_3$SO$_3^-$), 1031 (m, CF$_3$SO$_3^-$). HR-MS (ESI, positive mode, m/z): [M – CF$_3$SO$_3^-$]$^+$ calcd for ReC$_{20}$H$_{13}$N$_3$O$_4$ 546.0461, found 546.0445.

[Re(Ph$_2$-phen)(CO)$_3$(py-OCH$_2$-Tz-^{t}Bu)](CF$_3$SO$_3$) (**3a**)

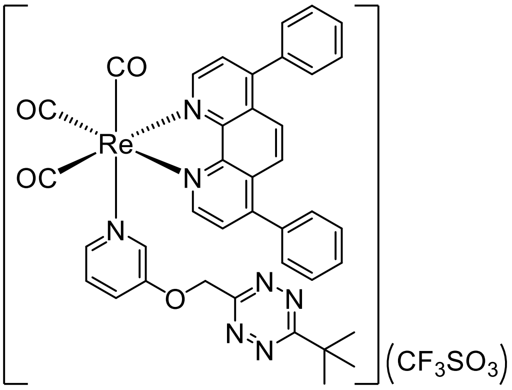

The synthetic procedure was similar to that for the preparation of complex **1a**, except that [Re(Ph$_2$-phen)(CO)$_3$(CH$_3$CN)](CF$_3$SO$_3$) (62 mg, 0.078 mmol) was used instead of [Re(Me$_2$-bpy)(CO)$_3$(CH$_3$CN)](CF$_3$SO$_3$). Subsequent recrystallization of the solid from CH$_2$Cl$_2$/Et$_2$O afforded the complex as dark pink crystals. Yield: 48 mg (62%).^{1}H NMR (300 MHz, CDCl$_3$, 298 K): δ 9.60 (d, J = 5.3 Hz, 2H, H2 and H9 of Ph$_2$-phen), 8.35 (d, J = 2.1 Hz, 1H, H2 of py), 8.17 (s, 2H, H5 and H6 of Ph$_2$-phen), 8.07 (d, J = 5.3 Hz, 2H, H3 and H8 of Ph$_2$-phen), 8.03 (d, J = 5.1 Hz, 1H, H6 of py), 7.66 – 7.58 (m, 11H, H5 of py and C$_6$H$_5$ at C4 and C7 of Ph$_2$-phen), 7.49 (dd, J = 7.9, 5.1 Hz, 1H, H4 of py), 5.65 (s, 2H, CH$_2$), 1.56 (s, 9H, (CH$_3$)$_3$). ^{13}C NMR (150 MHz, CDCl$_3$, 298 K): δ 177.15, 164.16, 156.17, 153.18, 153.10, 147.47, 145.34, 141.04, 134.96, 130.29, 129.91, 129.56, 129.30, 128.33, 127.45, 126.47, 125.56, 68.59, 65.85, 38.25, 29.10, 15.27. IR (KBr) $\tilde{\nu}$/cm^{-1}: 2032 (s, C≡O), 1922 (s, C≡O), 1151 (m, CF$_3$SO$_3^-$), 1032 (m, CF$_3$SO$_3^-$). HR-MS (ESI, positive mode, *m*/*z*): [M – CF$_3$SO$_3^-$]$^+$ calcd for ReC$_{39}$H$_{31}$N$_7$O$_4$ 848.2008, found 848.1966.

[Re(Ph_2-phen)$(CO)_3$(py-OH)](CF_3SO_3) (**3b**)

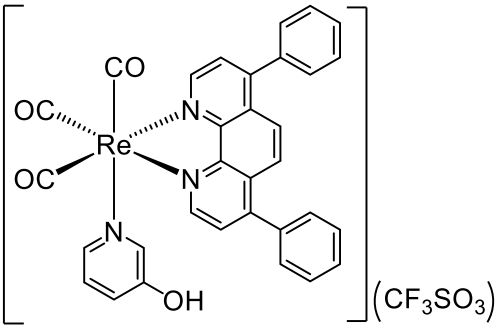

The synthetic procedure was similar to that for the preparation of complex **1b**, except that [Re(Ph_2-phen)$(CO)_3$(CH_3CN)](CF_3SO_3) (62 mg, 0.078 mmol) was used instead of [Re(Me_2-bpy)$(CO)_3$(CH_3CN)](CF_3SO_3). Subsequent recrystallization of the solid from CH_2Cl_2/Et_2O afforded the complex as dark pink crystals. Yield: 43 mg (65%). ^{1}H NMR (300 MHz, $(CD_3)_2CO$, 298 K): δ 9.97 (d, J = 5.4 Hz, 2H, H2 and H9 of Ph_2-phen), 9.67 (s, 1H, OH), 8.30 (d, J = 5.4 Hz, 2H, H5 and H6 of Ph_2-phen), 8.25 – 8.20 (m, 4H, H2 and H6 of py and H3 and H8 of Ph_2-phen), 7.76 – 7.67 (m, 10H, C_6H_5 at C4 and C7 of Ph_2-phen), 7.36 (dd, J = 8.5, 1.6 Hz, 1H, H5 of py), 7.23 (dd, J = 8.5, 5.4 Hz, 1H, H4 of py). ^{13}C NMR (150 MHz, $(CD_3)_2CO$, 298 K): δ 154.44, 152.53, 147.46, 143.51, 140.29, 135.45, 130.10, 129.87, 129.24, 129.14, 127.55, 127.15, 126.39, 126.15, 22.37, 13.43. IR (KBr) $\tilde{\nu}$/cm^{-1}: 2031 (s, C≡O), 1922 (s, C≡O), 1163 (m, $CF_3SO_3^-$), 1030 (m, $CF_3SO_3^-$). HR-MS (ESI, positive mode, *m*/*z*): [M – $CF_3SO_3^-$]$^+$ calcd for $ReC_{32}H_{21}N_3O_4$ 698.1089, found 698.1052.

3’-Hydroxy-6’-(3-isocyanopropoxy)-3*H*-spiro[isobenzofuran-1,9’-xanthen]-3-one (ICPr-fluorescein)

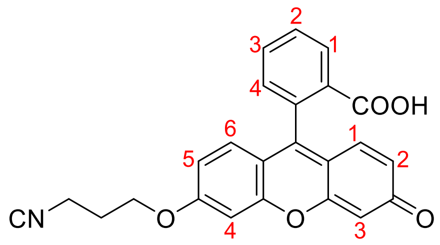

To a solution of fluorescein (60 mg, 0.18 mmol) in DMF (1 mL) was added Cs_2CO_3 (59 mg, 0.18 mmol), and the reaction mixture was stirred at 298 K for 15 min, which was followed by dropwise addition of ICPr-tos[4] (41 mg, 0.17 mmol) in DMF (0.5 mL), and the reaction mixture was stirred at 60°C for 4 h. After cooling to room temperature, the reaction mixture was filtered. The orange filtrate was diluted with CH_2Cl_2 (100 mL) and washed with water (50 mL × 3). The organic layer was dried over anhydrous $MgSO_4$, filtered, and the solvent was removed under reduced pressure. The orange residue was purified by column chromatography on silica gel using CH_2Cl_2/MeOH (100:1, *v*/*v*) as the eluent. The solvent was removed under reduced pressure to give an orange solid. Yield: 34 mg (50%). ^{1}H NMR (300 MHz, $CDCl_3$, 298 K): δ 8.05 – 7.99 (m, 1H, H1 of isobenzofuran), 7.70 – 7.58 (m, 2H, H3 and H4 of isobenzofuran), 7.16 (d, J = 7.4 Hz, 1H, H2 of isobenzofuran), 6.75 (dd, J = 4.4, 2.4 Hz, 2H, H1 and H6 of xanthene), 6.71 – 6.51 (m, 4H, H2 to H5 of xanthene), 4.13 (t, J = 5.6 Hz, 2H, OCH_2), 3.64 (t, J = 6.2 Hz, 2H, CH_2NC), 2.16 (t, J = 5.9 Hz, 2H, CH_2). MS (ESI, positive mode, *m*/*z*): 400.7 $[M + H]^+$.

Physical Measurements and Instrumentation

^{1}H and ^{13}C NMR spectra were recorded on a Bruker 300, 400, or 600 MHz AVANCE III spectrometer at 298 K using deuterated solvents. Chemical shifts (δ, ppm) were reported relative to tetramethylsilane (TMS) or the residual peak of the deuterated solvent ($CDCl_3$, 7.26 ppm; or $(CD_3)_2CO$, 2.05 ppm). Positive-ion ESI mass spectra were recorded on a SCIEX API-3200 Triple-Q MS/MS mass spectrometer at 298 K. HR-ESI mass spectra were recorded on a SCIEX X500R Q-TOF at 298 K. IR spectra of the samples in KBr pellets were recorded in the range of 4000 – 400 cm^{-1} using a Perkin Elmer Spectrum 100 FTIR spectrometer. HPLC was performed on an Agilent 1260 Infinity II system coupled with a diode array detector WR using H_2O containing 0.1% (*v*/*v*) TFA (solvent A) and CH_3CN containing 0.1% (*v*/*v*) TFA (solvent B) as the solvents, and the detector was set to 220, 250, 270, 325, 350, and 480 nm.

Photophysical Studies

Electronic absorption spectra were recorded on an Agilent 8453 diode array spectrophotometer. Steady-state emission spectra were recorded on a HORIBA FluoroMax-4 spectrofluorometer. Unless specified otherwise, all solutions for photophysical studies were degassed with no fewer than four successive freeze-pump-thaw cycles and stored in a 10-cm^3 round-bottomed flask equipped with a side-arm 1-cm fluorescence cuvette and sealed from the atmosphere by a Rotaflo HP6/6 quick-release Teflon stopper.

Emission quantum yields were measured by the optically dilute method[5] using a degassed CH_3CN solution of $[Re(phen)(CO)_3(py)](CF_3SO_3)$ (Φ_{em} = 0.18, λ_{ex} = 355 nm)[6] as the standard solution. The concentrations of the standard and sample solutions were adjusted until the absorbance at 355 nm was ≈ 0.1. The quantum yields of the sample were calculated according to the following equation:

$$\Phi_s = \Phi_r \left(\frac{I_r}{I_s}\right)\left(\frac{B_r}{B_s}\right)\left(\frac{n_s}{n_r}\right)^2\left(\frac{D_s}{D_r}\right)$$

where the subscripts *s* and *r* refer to the sample and reference solutions, respectively, Φ is luminescence quantum yield, *I* is excitation intensity, *B* is $1 - 10^{-AL}$, *A* is absorbance at the excitation wavelength, *L* is path length in cm, *n* is refractive index of the solvent, and *D* is integrated intensity.

The emission lifetimes were measured on an Edinburgh Instruments LP920 laser flash photolysis spectrometer using the third harmonic output (355 nm; 6 – 8 ns fwhm pulse width) of a Spectra-Physics Quanta-Ray Q-switched LAB-150 pulsed Nd:YAG laser (10 Hz) as the excitation source. The lifetime τ_o was determined by a single exponential fitting of the luminescence decay trace with the relationship $I = I_o \exp(-t/\tau_o)$, where I_o and I are the luminescence intensities at time = 0 and *t*, respectively.

Bioorthogonal Reactivity

The TzMe complexes **1a** – **3a** (10 μM) were incubated with ICPrc-Bn (500 μM) in aerated H_2O/DMSO (4:1, *v*/*v*) or McIlvaine buffer (pH 7.4 or 5.0)/DMSO (4:1, *v*/*v*) at 37°C for 18 h. The emission spectra were recorded on a HORIBA FluoroMax-4 spectrofluorometer, and the emission lifetimes were measured on an Edinburgh Instruments LP920 laser flash photolysis spectrometer. The reaction mixtures were subjected to ESI-MS analysis. Additionally, complex **3a** (10 μM) was incubated with BCN-OH (500 μM) in aerated H_2O/DMSO (4:1, *v*/*v*) at 37°C for 18 h. The reaction mixture was subjected to ESI-MS analysis.

Determination of Singlet Oxygen ($^1O_2$) Generation Quantum Yields (Φ_Δ)

The $^1O_2$ generation quantum yields (Φ_Δ) were measured by the optically dilute method[5] using phenalenone in aerated benzene (Φ_Δ = 0.97)[7] as a reference for $^1O_2$ sensitization. An air-equilibrated CH_3CN solution (2 mL) containing the complex was introduced to a quartz cuvette of 1-cm path length. The concentrations of the reference and sample solutions were adjusted until the absorbance at 350 nm was ≈ 0.15. The solutions were excited at λ = 350 nm, and the emission spectra of $^1O_2$ at 1200 – 1350 nm were recorded on an Edinburgh Instruments FLS980 spectrometer equipped with an R5509-73 NIR photomultiplier tube and C9940-02 exclusive coolers. The Φ_Δ value of the complex was determined using the following equation:

$$\Phi_s = \Phi_r \left(\frac{I_r}{I_s}\right)\left(\frac{B_r}{B_s}\right)\left(\frac{n_s}{n_r}\right)^2\left(\frac{D_s}{D_r}\right)$$

where the subscripts *s* and *r* refer to the sample and reference solutions, respectively, Φ is $^1O_2$ quantum yield, *I* is excitation intensity, *B* is $1 - 10^{-AL}$, *A* is absorbance at the excitation wavelength, *L* is path length in cm, *n* is refractive index of the solvent, and *D* is integrated intensity.

To assess the pH effect on the $^1O_2$ generation efficiencies of the complexes, a steady-state method was used with ABDA as the $^1O_2$ indicator[8] and riboflavin in aerated H_2O (Φ_Δ = 0.54)[9] as the reference. An air-equilibrated McIlvaine buffer (pH 3.0, 5.0, and 7.4)/CH_3CN (4:1, *v*/*v*) solution (2 mL) containing complex **3a** or **3b** and ABDA (100 μM) was introduced to a quartz cuvette of 1-cm path length. The concentrations of the reference and sample solutions were adjusted until absorbance at the excitation wavelength (355 nm) was ≈ 0.15. The solutions were irradiated at 355 nm using a Xenon lamp (Ushio) (150 W) with a bandwidth of 20 nm. The absorbance of ABDA at ≈ 378 nm was monitored at time intervals. The Φ_Δ value of the complex was determined using the following equation:

$$\Phi_s = \Phi_r \times \frac{m_s \times B_r}{m_r \times B_s}$$

where the subscripts s and r refer to the sample and reference solutions, respectively, Φ is $^1O_2$ quantum yield, m is the slope of a linear fit of the change of absorbance at 378 nm against the irradiation time, B is $1 - 10^{-AL}$, A is absorbance at the excitation wavelength, and L is path length in cm.

Dual-release Studies

To confirm the liberation of ICPr/ICPrc-caged compounds during the reaction, the concurrent release of compounds from their respective tetrazine and ICPr/ICPrc caging groups was studied by RP-HPLC analysis. Complex **3a** (50 μM) and ICPr-coum (40 μM) were incubated in aerated McIlvaine buffer (pH 7.4)/DMSO (4:1, *v*/*v*) at 37°C for 18 h. The reaction mixture was subjected to HPLC analysis. The HPLC analysis was performed using an Agilent analytical column (ZORBAX Eclipse Plus C18 column: 4.6 × 150 mm, 5 μm) with a linear gradient of 0 − 100% solvent B over 12 min and a flow rate of 1 mL min^{-1}. The emission was monitored at the emission peak maxima of the released products, umbelliferone (also known as 7-hydroxycoumarin) (λ_{ex} = 350 nm, λ_{em} = 460 nm) and complex **3b** (λ_{ex} = 350 nm, λ_{em} = 562 nm). Additionally, the product peak at t_R = 6.5 min was collected and characterized by ESI-MS (*m*/*z*: 163.1 $[M + H]^+$, consistent with umbelliferone) and emission spectroscopy. The emission spectrum was recorded on a HORIBA FluoroMax-4 spectrofluorometer. To confirm the dual release of complex **3b** and ICPr/ICPrc-caged payload before conducting cellular studies, complex **3a** (10 μM) and ICPr-fluorescein or ICPrc-Dox (10 μM) were incubated in aerated PBS/DMSO (4:1, *v*/*v*) at 37°C. The reaction was monitored by HPLC analyses after incubation for 4 and 24 h.

Kinetic Studies

All reactions were performed on a 100-μL scale. The reaction kinetics of the tetrazine complexes **1a** – **3a** (20 μM) with ICPr-coum (25 μM) in aerated McIlvaine buffer (pH 7.4 or 5.0)/DMSO (4:1, *v*/*v*) at 37°C was measured by RP-HPLC. The reaction kinetics of the ligand py-OCH_2-Tz-^{t}Bu (200 μM) with ICPr-coum (250 μM) in aerated McIlvaine buffer (pH 7.4 or 5.0)/CH_3CN (4:1, *v*/*v*) at 37°C was measured by RP-HPLC. The substitution of DMSO with CH_3CN was necessary to address the issue of the product py-OH peak overlapping with the solvent peak. Consequently, the outcomes obtained for the ligand solely served as a reference. The reactions at different time points were quenched by the addition of 900 μL of cold H_2O/CH_3CN (1:1, *v*/*v*) and then analyzed by RP-HPLC. The HPLC analysis was carried out using an Agilent analytical column (ZORBAX Eclipse Plus C18 column: 4.6 × 150 mm, 5 μm) with a linear gradient of 0 − 100% solvent B over 12 min and a flow rate of 1 mL min^{-1} for the complexes. As for the ligand, the elution profile was as follows: an isocratic elution at 0% solvent B over 10 min, followed by a linear gradient of 0 – 60% solvent B over 10 min. The k_2 values were determined by fitting the data to the following equation:

$$y = \frac{ln\frac{[\mathrm{A}]_\mathrm{o}[\mathrm{B}]_t}{[\mathrm{A}]_t[\mathrm{B}]_\mathrm{o}}}{([\mathrm{B}]_\mathrm{o} - [\mathrm{A}]_\mathrm{o})} = k_2 t$$

where $[A]_o$ and $[A]_t$ are the concentrations of the tetrazine-containing compound (ligand or complex) at time = 0 and *t* s, respectively; and $[B]_o$ and $[B]_t$ are the concentrations of ICPr-coum at time = 0 and *t* s, respectively. All kinetic curves generated using the OriginPro 8.0 software package are summarized in Figures S8 and S12.

To determine the half-lives and release yields of ICPr/ICPrc derivatives in the reaction, complexes **1a** – **3a** (50 μM) was incubated with ICPr-coum (20 μM) in aerated McIlvaine buffer (pH 7.4 or 5.0)/DMSO (4:1, *v*/*v*) at 37°C, whereas ligand py-OCH_2-Tz-^{t}Bu (200 μM)

was incubated with ICPr-coum (80 μM) in aerated McIlvaine buffer (pH 7.4 or 5.0)/CH_3CN (4:1, *v*/*v*) at 37°C. The reactions at different time points were quenched by the addition of 900 μL of cold H_2O/CH_3CN (1:1, *v*/*v*) and then analyzed by RP-HPLC. The HPLC analysis was conducted following the elution conditions specified for the reaction kinetic studies. The release yields were plotted against time. The half-lives ($t_{1/2}$) were defined as the time required for the concentration of the reactant to decrease by half of its initial quantity, obtained through an exponential fit of the curve. The release yields were defined as the quantity of the final product, umbelliferone, liberated in the reaction. Furthermore, to evaluate the effect of ether versus carbamate linkages within the ICPr/ICPrc moiety on release kinetics, the half-lives of ICPr-coum, ICPr-fluorescein, and ICPrc-Dox were determined by HPLC analyses. Equimolar mixtures of complex **3a** (50 μM) and each ICPr/ICPrc derivative (50 μM) were incubated in aerated McIlvaine buffer (pH 7.4)/DMSO (4:1, *v*/*v*) at 37°C. The reactions at different time points were quenched by the addition of 900 μL of cold H_2O/CH_3CN (1:1, *v*/*v*) and then analyzed by RP-HPLC.

Stability of ICPr/ICPrc Derivatives in Serum

ICPr-fluorescein or ICPrc-Dox (25 μM) was incubated in 25% FBS at 37°C in the dark for 48 h. Following incubation, cold CH_3CN (4 equivalents) was added to precipitate the serum proteins, and the mixture was centrifuged at 14,000 rpm for 15 min. The supernatant was filtered through a 0.2-μm membrane filter and subjected to HPLC analysis. The HPLC analysis was carried out with a linear gradient of 0 − 100% solvent B over 12 min and a flow rate of 1 mL min^{-1}.

Cell Cultures

HeLa and HEK-293 cells were grown in DMEM supplemented with 10% FBS and 1% penicillin/streptomycin at 37°C under a 5% CO_2 atmosphere. They were subcultured every 2 to 3 days.

ESI-MS Analysis of the Cell Lysates

HeLa cells in growth medium were seeded in a 5-mm tissue culture dish and incubated at 37°C under a 5% CO_2 atmosphere for 72 h. The growth medium was removed, and the cells were washed with PBS and incubated with complex **3a** (10 μM) in growth medium/DMSO (99:1, *v*/*v*) at 37°C for 3 h. The cell layer was washed with PBS (1 mL × 3) and then incubated with ICPrc-Bn (200 μM) in growth medium/DMSO (99:1, *v*/*v*) or fresh growth medium at 37°C for 4 h. The growth medium was removed, and the cell layer was washed with PBS (1 mL × 3). The cells in each dish were trypsinized and harvested with PBS (2 mL). The resultant solutions were subjected to probe sonication with 90 cycles of 10 seconds on, 10 seconds off, at 80% power on an ice bath. The mixture was extracted with CH_2Cl_2 (1 mL × 3). The organic extract was dried over anhydrous $MgSO_4$, filtered, and the solvent was removed under reduced pressure. The residue was dissolved in MeOH and analyzed by ESI-MS.

Live-cell Confocal Imaging

HeLa cells in growth medium were seeded in a 35-mm confocal dish and incubated at 37°C under a 5% CO_2 atmosphere for 48 h. The growth medium was removed, and the cell layer was washed with PBS (1 mL × 3) and incubated with complexes **1a** – **3a** (10 μM) in growth medium/DMSO (99:1, *v*/*v*) at 37°C for 3 h. The cell layer was washed with PBS (1 mL × 3) and then incubated with ICPrc-Bn (200 μM) in growth medium/DMSO (99:1, *v*/*v*) at 37°C for 4 h. After washing with PBS (1 mL × 3), the cells were imaged using a Leica Point Scanning

Confocal Microscope (Stellaris 8) with an oil-immersion 63× objective lens and an excitation wavelength of 405 nm. For the co-staining experiments, after HeLa cells were treated with the complexes and ICPrc-Bn, the growth medium was removed, and the cell layer was washed with PBS (1 mL × 3). The cells were then incubated with LysoTracker Deep Red (75 nM) at 37°C for 1 h or MitoTracker Deep Red (100 nM) at 37°C for 20 min. The growth medium was then removed, and the cell layer was washed with PBS (1 mL × 3). The excitation wavelength of LysoTracker Deep Red and MitoTracker Deep Red was 635 nm. The Pearson's correlation coefficients (PCC's) were determined using the program ImageJ (Version 1.4.3.67). For dual-release studies, the cells were incubated with ICPr-coum (200 μM) or ICPr-fluorescein (10 μM) alone at 37°C for 4 h, or incubated with complex **3a** (10 μM) at 37°C for 3 h, followed by incubation without or with ICPr-coum or ICPr-fluorescein at 37°C for 4 h. The excitation wavelengths of ICPr-coum and ICPr-fluorescein were 405 nm and 488 nm, respectively.

Cellular Uptake Measurements

HeLa and HEK-293 cells in growth medium were seeded in a 35-mm tissue culture dish and incubated at 37°C under a 5% CO_2 atmosphere for 72 h. The growth medium was removed, and the cell layer was washed with PBS (1 mL × 3) and incubated with complexes **1a** – **3a** (10 μM) in growth medium/DMSO (99:1, *v*/*v*) at 37°C for 3 h. The growth medium was removed, and the cell layer was washed thoroughly with PBS (1 mL × 3). The cells were trypsinized and harvested with PBS (1 mL). Cell numbers were obtained by a Logos Biosystems LUNA-II automated cell counter. The resultant solution was digested with 65% HNO_3 (1 mL) at 70°C for 2 h, allowed to cool to room temperature, and analyzed by a NexION 2000 ICP-MS (PerkinElmer SCIEX Instruments).

For cellular uptake mechanism studies of complex **3a**, HeLa cells were treated with the complex (10 μM) in growth medium/DMSO (99:1, *v*/*v*) at 37°C for 1 h. In the low-temperature experiments, the cells were preincubated at 4°C for 1 h prior to incubation with complex **3a** in growth medium/DMSO (99:1, *v*/*v*) at 37°C for 1 h. In the chemical inhibition experiments, the cells were pretreated with EIPA (50 μM) for 1.5 h, Me-β-CD (5 mM) for 1 h, or chlorpromazine (30 μM) in growth medium for 1 h at 37°C under a 5% CO_2 atmosphere. The cell layer was then washed with PBS (1 mL × 3) and incubated with complex **3a** (10 μM) in growth medium/DMSO (99:1, *v*/*v*) at 37°C for 1 h.

Flow Cytometry

HeLa cells in growth medium were seeded in a 35-mm tissue culture dish and incubated at 37°C under a 5% CO_2 atmosphere for 72 h. The growth medium was removed, and the cell layer was washed with PBS (1 mL × 3) and incubated with complexes **1a** – **3a** (10 μM) in growth medium/DMSO (99:1, *v*/*v*) at 37°C for 3 h. The cells were washed with PBS (1 mL × 3) and then incubated with ICPrc-Bn (200 μM) in growth medium/DMSO (99:1, *v*/*v*) or fresh growth medium at 37°C for 4 h. The growth medium was removed, and the cell layer was washed with PBS (1 mL × 3). The cells in each dish were trypsinized and harvested with PBS (1 mL). The resultant cell suspensions were analyzed by a CytoFLEX Flow Cytometer (Beckman Coulter, Brea, California, USA) with an excitation wavelength of 405 nm and an emission wavelength of 525 nm. The number of cells analyzed for each sample was ≈ 10,000.

MTT Assays

HeLa and HEK-293 cells were seeded in a 96-well flat-bottomed microplate (≈ 10,000 cells per well) in growth medium (100 μL) and incubated at 37°C under a 5% CO_2 atmosphere for

48 h. The growth medium was removed, and then incubated with either fresh growth medium or complexes **1a** – **3a**, with concentrations ranging from 10^{-5} to 10^{-8} M, in growth medium/DMSO (99:1, *v*/*v*) at 37°C for 3 h. The growth medium was removed and replaced with either fresh growth medium or ICPrc-Bn (200 µM) in growth medium/DMSO (99:1, *v*/*v*) at 37°C for 4 h. Wells containing untreated cells were used as a blank control. Wells containing cells treated with ICPrc-Bn (200 µM) alone for 4 h were used as a complex-free control. For evaluation of photocytotoxicity, the treated cells were incubated in phenol red-free growth medium (100 µL). One of the microplates was irradiated at 365 nm (5 mW cm^{-2}) with an LED cellular photocytotoxicity irradiator (PURI Materials, Shenzhen, China) for 5 min, whereas the other one was kept in the dark for 10 min. The cells were then incubated in fresh growth medium (100 µL) in the dark for 24 h. A solution containing MTT in PBS (10 µL, 5 mg mL^{-1}) and fresh growth medium (90 µL) was added to each well. The microplate was incubated at 37°C for 4 h. The growth medium was removed, and DMSO (200 µL) was added to each well. After 20 min, the absorbance of the solutions at 570 nm was measured with a Multiskan SkyHigh Microplate Spectrophotometer (Thermo Scientific). The IC_{50} values of the complex were determined from the dose dependence of surviving cells after the treatment using the OriginPro 8.0 software package. For dual-release studies, the cells were incubated with complex **3a**, with concentrations ranging from 10^{-5} to 10^{-8} M, in growth medium/DMSO (99:1, *v*/*v*) at 37°C for 3 h, and replaced with either fresh growth medium or ICPrc-Dox (10 or 50 µM) in growth medium/DMSO (99:1, *v*/*v*) at 37°C for 4 h. Wells containing cells treated with ICPrc-Dox (10 or 50 µM) alone for 4 h were used as a complex-free control. Wells containing cells treated with cisplatin (3 h) or Dox (4 h), with concentrations ranging from 10^{-5} to 10^{-8} M, in growth medium/DMSO (99:1, *v*/*v*) at 37°C were used as positive controls. Furthermore, to evaluate the maximum therapeutic effect of the dual release, concentrations of complex **3b** and Dox were serially diluted in a 1:1 ratio to serve as a reference, assuming similar cellular uptake

of complex **3a** with **3b** and ICPrc-Dox with Dox. Briefly, HeLa cells were incubated with complex **3b**, with concentrations ranging from 10^{-5} to 10^{-8} M, in growth medium/DMSO (99:1, *v*/*v*) at 37°C for 3 h, and replaced with either fresh growth medium or Dox, with concentrations ranging from 10^{-5} to 10^{-8} M, in growth medium/DMSO (99:1, *v*/*v*) at 37°C for 4 h.

Table S1. Electronic absorption spectral data of the rhenium(I) complexes and ligands py-OCH_2-Tz-^{t}Bu and py-OH at 298 K.

Compound	Solvent	λ_{abs}/nm (ε/dm^3 mol^{-1} cm^{-1})
**1a**	CH_2Cl_2	277 (20,075), 306 sh (11,860), 318 (10,625), 363 sh (3555), 548 (370)
	CH_3CN	269 (20,530), 303 sh (12,460), 317 (12,515), 350 sh (3885), 544 (400)
**1b**	CH_2Cl_2	254 (16,795), 278 (18,025), 305 sh (12,240), 318 (10,395), 348 (3805), 385 sh (2140)
	CH_3CN	251 (18,695), 266 (17,820), 303 sh (11,985), 317 (11,715), 355 sh (3630)
**2a**	CH_2Cl_2	257 sh (23,040), 277 (27,640), 328 sh (5520), 386 sh (3345), 544 (470)
	CH_3CN	257 sh (24,495), 274 (31,285), 326 sh (6765), 368 sh (4070), 537 (535)
**2b**	CH_2Cl_2	259 (30,570), 275 (31,895), 297 sh (16,775), 331 sh (6345), 400 sh (3155)
	CH_3CN	255 (28,815), 274 (31,425), 295 sh (16,675), 330 sh (7435), 380 sh (3800)
**3a**	CH_2Cl_2	272 sh (33,120), 293 (44,600), 344 sh (16,700), 393 sh (8620), 550 (695)
	CH_3CN	265 sh (34,250), 291 (48,570), 333 sh (19,155), 386 sh (8675), 543 (705)

**3b**	CH_2Cl_2	265 sh (29,805), 292 (45,960), 340 sh (15,420), 390 sh (8445)
	CH_3CN	262 sh (31,140), 291 (50,370), 336 sh (17,225), 390 sh (7760)
py-OCH_2-Tz-^{t}Bu	CH_2Cl_2	271 (6335), 308 sh (395), 546 (530)
	CH_3CN	271 (5230), 302 sh (415), 540 (415)
py-OH	CH_2Cl_2	276 (3220), 284 sh (2190)
	CH_3CN	276 (3330), 286 sh (1995)

Table S2. Photophysical data of the rhenium(I) complexes.

Complex	Medium (T/K)	λ_{em}/nm[a]	τ_o/μs[b]	Φ_{em}[c]
**1a**	CH_2Cl_2 (298)	523, 584 (max)	0.78	0.002
	CH_3CN (298)	533, 579 (max)	0.42	0.001
	H_2O[d] (298)	568	0.22	0.001
	Glass[e] (77)	485 (max), 572	0.12	
**1b**	CH_2Cl_2 (298)	535	0.66	0.15
	CH_3CN (298)	548	0.31	0.053
	H_2O[d] (298)	551	0.19	0.028
	Glass[e] (77)	495	4.31	
**2a**	CH_2Cl_2 (298)	530	3.00	0.014
	CH_3CN (298)	547	1.67	0.006
	H_2O[d] (298)	549	0.93	0.003
	Glass[e] (77)	495 (max), 568	0.20	
**2b**	CH_2Cl_2 (298)	538	2.97	0.31
	CH_3CN (298)	553	1.48	0.14
	H_2O[d] (298)	549	0.92	0.077
	Glass[e] (77)	498	9.57	
**3a**	CH_2Cl_2 (298)	545	9.33	0.020
	CH_3CN (298)	561	4.73	0.009
	H_2O[d] (298)	562	2.15	0.003
	Glass[e] (77)	510, 560 sh	0.32	

**3b**	CH_2Cl_2 (298)	548	8.32	0.43
	CH_3CN (298)	564	3.07	0.17
	H_2O^d (298)	559	2.26	0.050
	Glass^e (77)	511, 534 sh	20.32	

^{a} λ_{ex} = 355 nm.

^{b} The lifetimes were measured at the emission maxima.

^{c} The emission quantum yields were determined using [Re(phen)(CO)$_3$(py)](CF$_3$SO$_3$) (Φ_{em} = 0.18 in degassed CH_3CN, λ_{ex} = 355 nm)[6] as a reference.

^{d} H_2O/MeOH (4:1, *v*/*v*).

^{e} EtOH/MeOH (4:1, *v*/*v*).

Table S3. Emission wavelengths (λ_{em}), emission lifetimes (τ), and emission enhancement factors (I/I_o) of complexes **1a** – **3a** (10 μM) upon incubation without or with ICPrc-Bn (500 μM) in aerated H_2O/DMSO (4:1, *v*/*v*) at 37°C for 18 h.

	– ICPrc-Bn		+ ICPrc-Bn		
Complex	λ_{em}/nm	τ/μs	λ_{em}/nm	I/I_o [a]	τ/μs
**1a**	546	0.18	549	21.7	0.18
**2a**	549	0.59	551	22.3	0.59
**3a**	566	0.97	566	13.4	0.99

[a] I_o and I are the emission intensities of the complexes in the absence and presence of ICPrc-Bn, respectively.

Table S4. Emission wavelengths (λ_{em}), emission lifetimes (τ), and emission enhancement factors (I/I_o) of complexes **1a** – **3a** (10 μM) upon incubation without or with ICPrc-Bn (500 μM) in aerated McIlvaine buffer (pH 5.0)/DMSO (4:1, *v*/*v*) at 37°C for 18 h.

	– ICPrc-Bn		+ ICPrc-Bn		
Complex	λ_{em}/nm	τ/μs	λ_{em}/nm	I/I_o [a]	τ/μs
**1a**	535	0.18	550	14.6	0.11
**2a**	549	0.63	551	6.2	0.20
**3a**	565	0.99	566	5.3	0.33

[a] I_o and I are the emission intensities of the complexes in the absence and presence of ICPrc-Bn, respectively.

Table S5. Emission wavelengths (λ_{em}), emission lifetimes (τ), and emission enhancement factors (I/I_o) of complexes **1a** – **3a** (10 μM) upon incubation without or with ICPrc-Bn (500 μM) in aerated McIlvaine buffer (pH 7.4)/DMSO (4:1, *v*/*v*) at 37°C for 18 h.

	– ICPrc-Bn		+ ICPrc-Bn		
Complex	λ_{em}/nm	τ/μs	λ_{em}/nm	I/I_o [a]	τ/μs
**1a**	540	0.19	540	1.0	0.19
**2a**	548	0.65	542	< 1.0	0.59
**3a**	563	1.14	560	< 1.0	0.97

[a] I_o and I are the emission intensities of the complexes in the absence and presence of ICPrc-Bn, respectively.

Table S6. $^1O_2$ generation quantum yields (Φ_Δ) of the rhenium(I) complexes in aerated CH_3CN at 298 K.

Complex	Φ_Δ[a]
**1a**	0.42
**2a**	0.57
**3a**	0.66
**1b**	0.38
**2b**	0.36
**3b**	0.63

[a] λ_{ex} = 350 nm and phenalenone in aerated benzene (Φ_Δ = 0.97)[7] was adopted as the reference.

Table S7. $^1O_2$ generation quantum yields (Φ_Δ) of complexes **3a** and **3b** in aerated McIlvaine buffer (pH 3.0, 5.0, and 7.4)/CH_3CN (4:1, *v*/*v*) at 298 K using ABDA as the $^1O_2$ indicator.[a]

pH	Φ_Δ (**3a**)	Φ_Δ (**3b**)
3.0	0.35	0.24
5.0	0.33	0.12
7.4	0.36	–[b]

[a] λ_{ex} = 355 nm and riboflavin in aerated H_2O (Φ_Δ = 0.54)[9] was adopted as the reference.

[b] Negligible.

Table S8. Flow cytometric results of HeLa cells incubated with complexes **1a** – **3a** (10 μM) for 3 h, followed by incubation with ICPrc-Bn (200 μM) or fresh medium for 4 h.

Entry	Relative Intensity/A. U.
Blank	1527.2
**1a**	1690.3
**1a** + ICPrc-Bn	2646.9
**2a**	1693.5
**2a** + ICPrc-Bn	2717.5
**3a**	3376.6
**3a** + ICPrc-Bn	10,735.9

Table S9. Cellular uptake of complexes **1a** – **3a** in HeLa and HEK-293 cells.

Complex	Amount of rhenium/fmol[a]	
	HeLa	HEK-293
**1a**	2.6 ± 0.1	0.61 ± 0.05
**2a**	2.7 ± 0.1	0.80 ± 0.07
**3a**	9.2 ± 0.3	3.8 ± 0.1

[a] Amount of rhenium associated with an average cell upon incubation with the complexes (10 μM) at 37°C for 3 h, as determined by ICP-MS.

Table S10. (Photo)cytotoxicity of complex **3b** toward HeLa cells without or with ICPrc-Dox post-treatment in the dark and upon irradiation at 365 nm (5 mW cm^{-2}) for 5 min.[a] Photocytotoxicity index (PI) is the ratio $IC_{50,dark}/IC_{50,light}$.

Entry	$IC_{50,dark}$/μM	$IC_{50,light}$/μM	PI
**3b**	29 ± 3	5.1 ± 0.5	6
**3b** + Dox	3.8 ± 0.3	1.9 ± 0.1	2

[a] The cells were first treated with complex **3b** for 3 h, and then incubated with either fresh growth medium or ICPrc-Dox in the dark for 4 h, replaced with fresh growth medium, followed by incubation in the dark or exposure to irradiation in fresh growth medium.

Scheme S1. Synthesis of the ligand py-OCH_2-Tz-^{t}Bu.

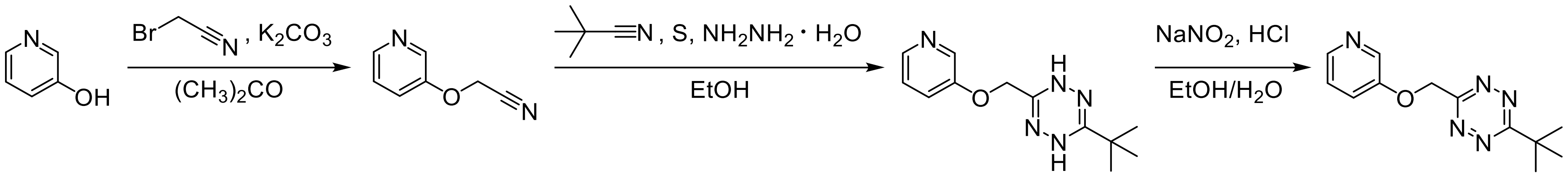

py-OCH_2-Tz-^{t}Bu

Figure S1. Electronic absorption spectra of the rhenium(I) complexes and ligands py-OCH_2-Tz-^{t}Bu and py-OH in CH_2Cl_2 (black) and CH_3CN (red) at 298 K.

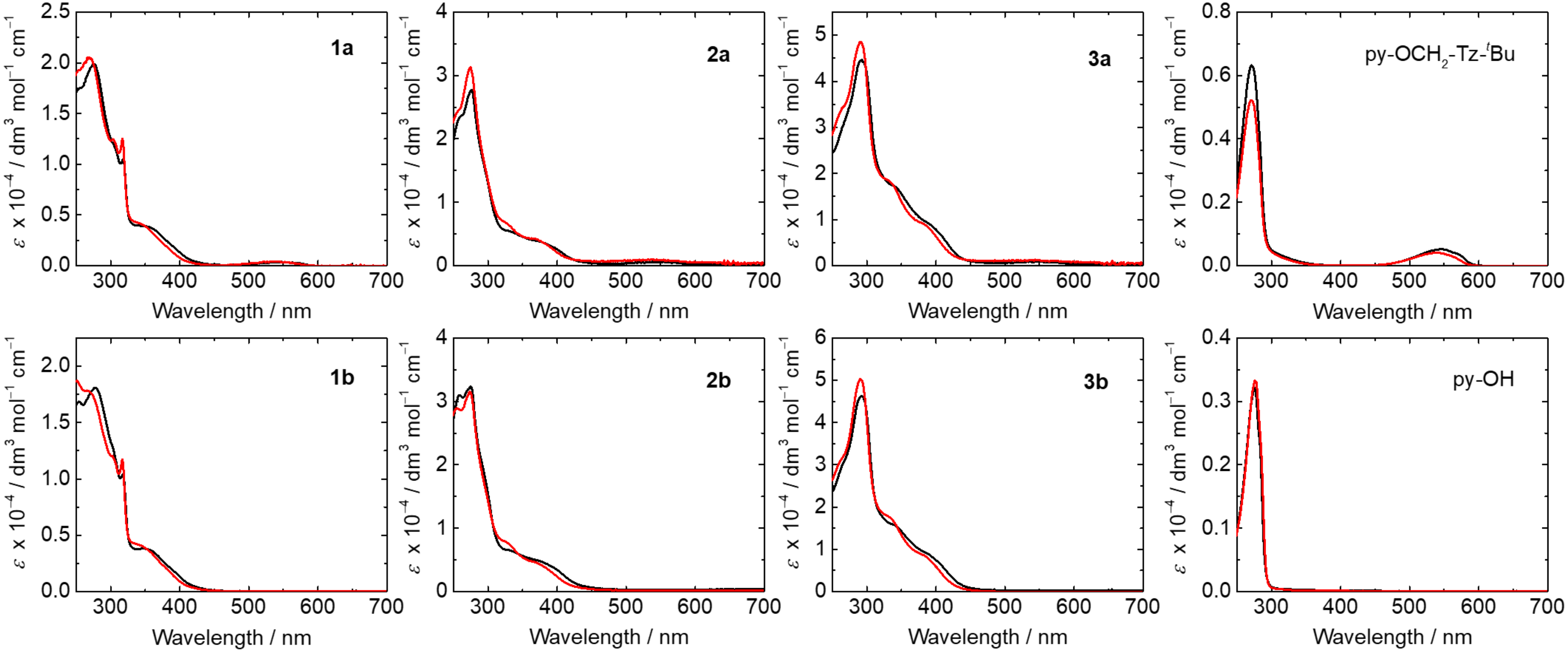

Figure S2. Emission spectra of the rhenium(I) complexes in CH_2Cl_2 (black) and CH_3CN (red) at 298 K and alcohol glass at 77 K (blue).

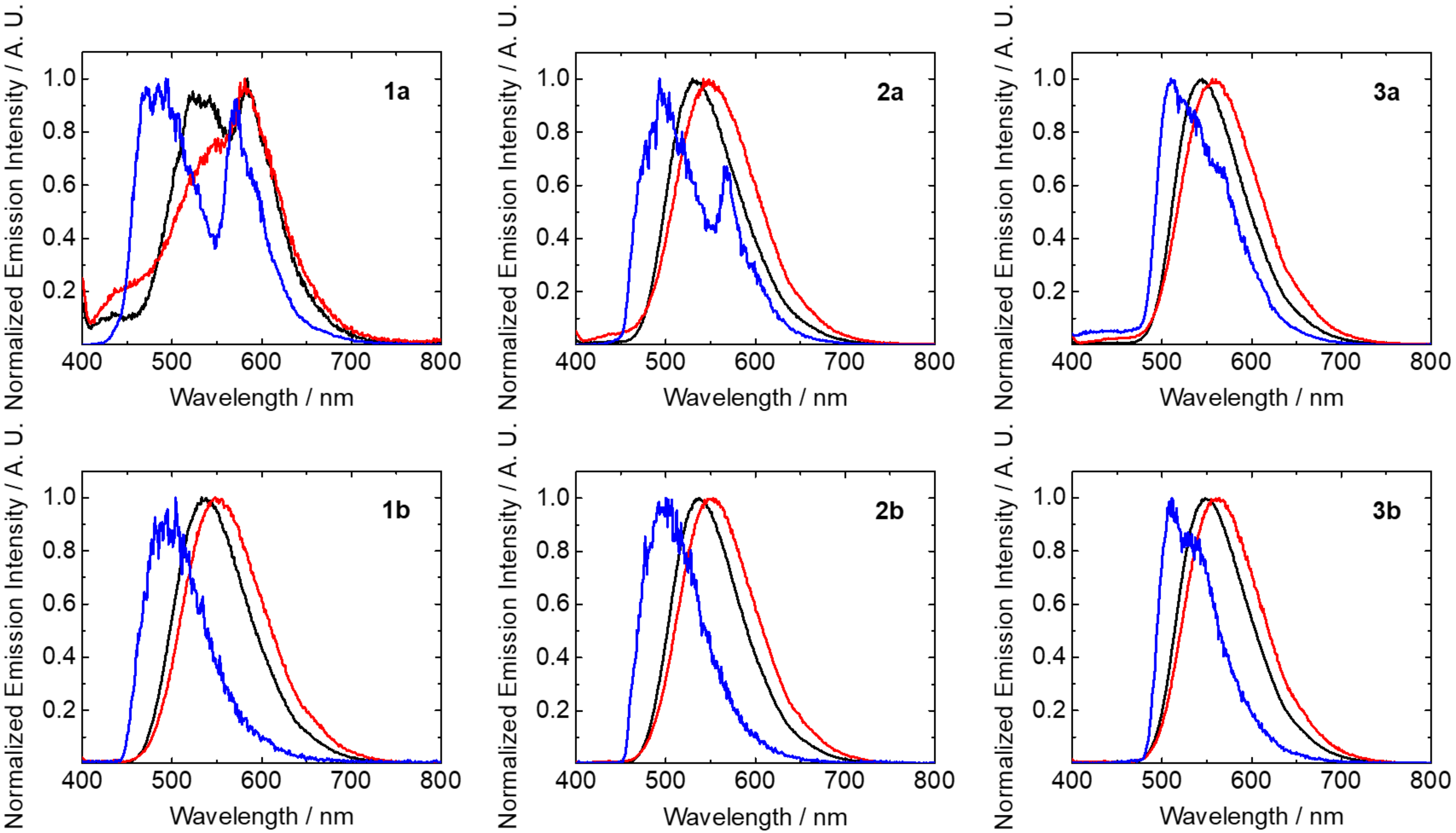

Figure S3. ESI mass spectra of the reaction mixture of complexes **1a** – **3a** (10 μM) with ICPrc-Bn (500 μM) in aerated H_2O/DMSO (4:1, *v*/*v*) after incubation at 37°C for 18 h.

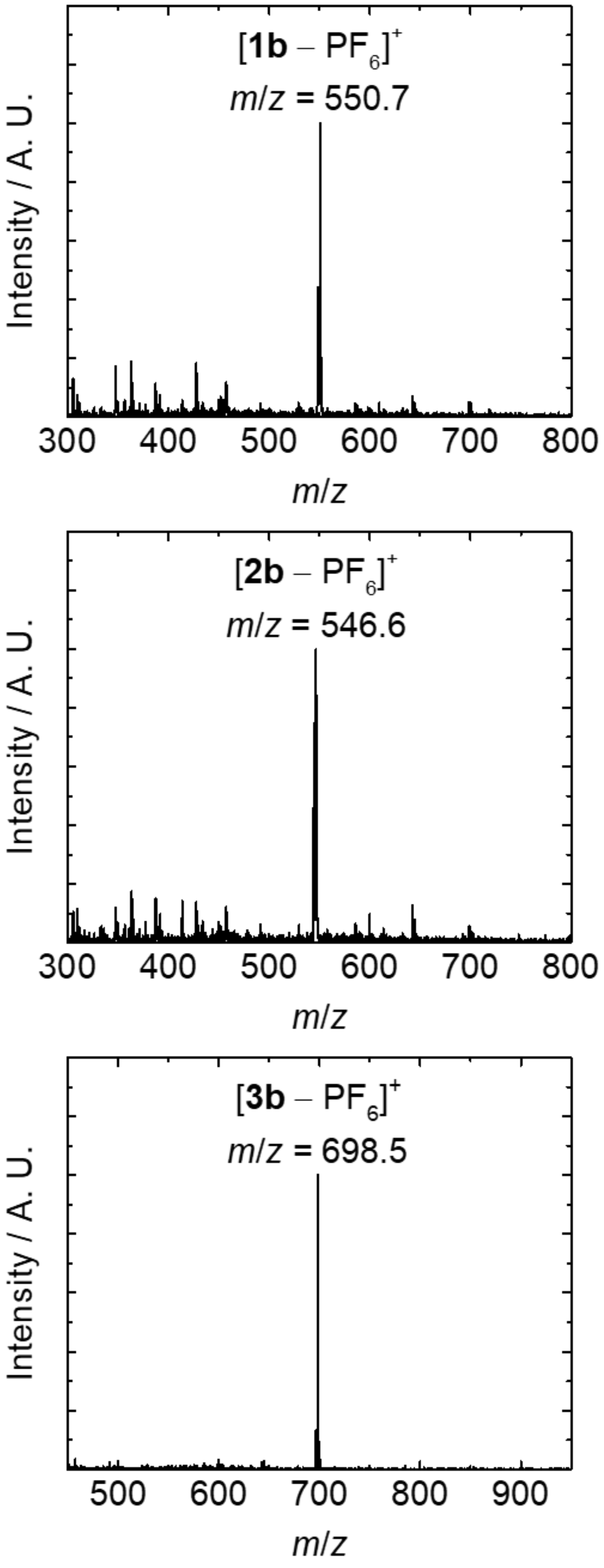

Figure S4. Emission spectra of complexes **1a** – **3a** (10 μM) before (black) and after (red) incubation with ICPrc-Bn (500 μM) in aerated McIlvaine buffer (pH 7.4)/DMSO (4:1, *v*/*v*) at 37°C for 18 h.

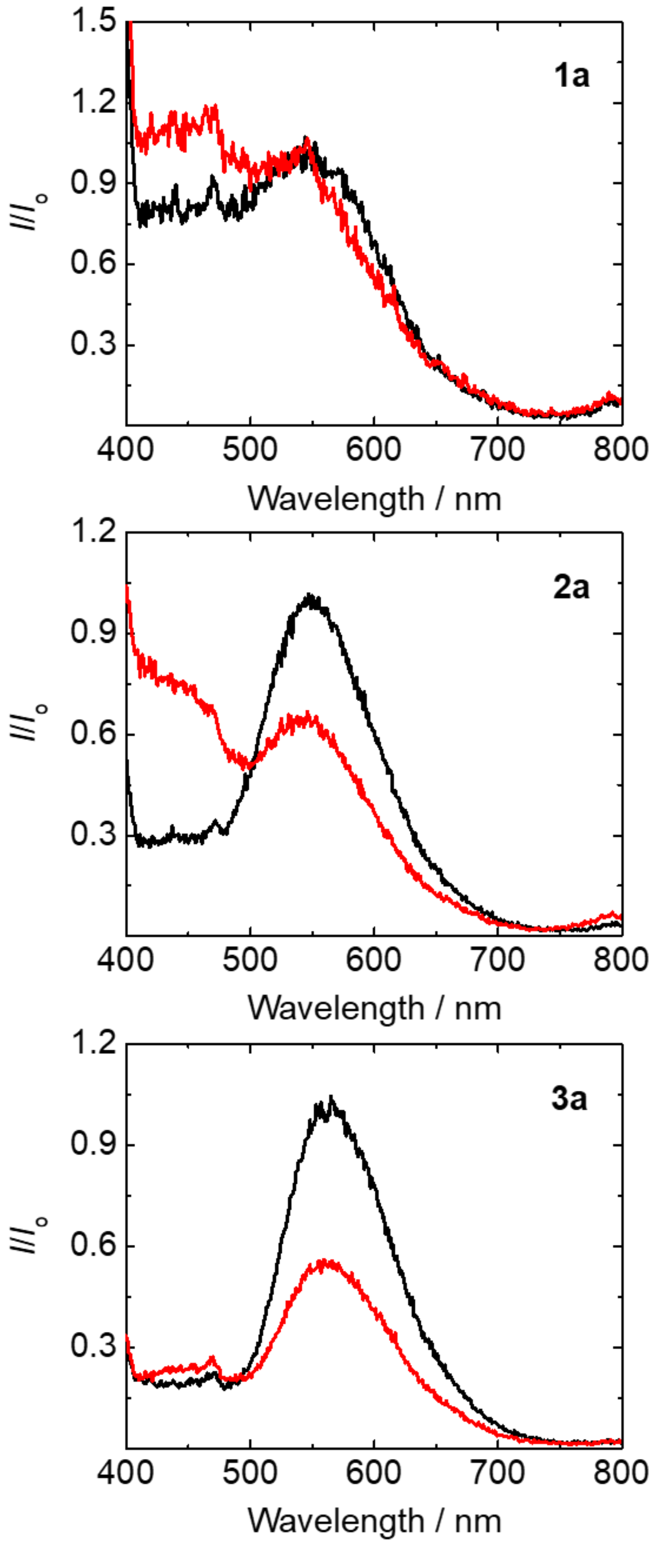

Figure S5. ESI mass spectrum of the reaction mixture of complex **3a** (10 μM) with BCN-OH (500 μM) in aerated H_2O/DMSO (4:1, *v*/*v*) after incubation at 37°C for 18 h.

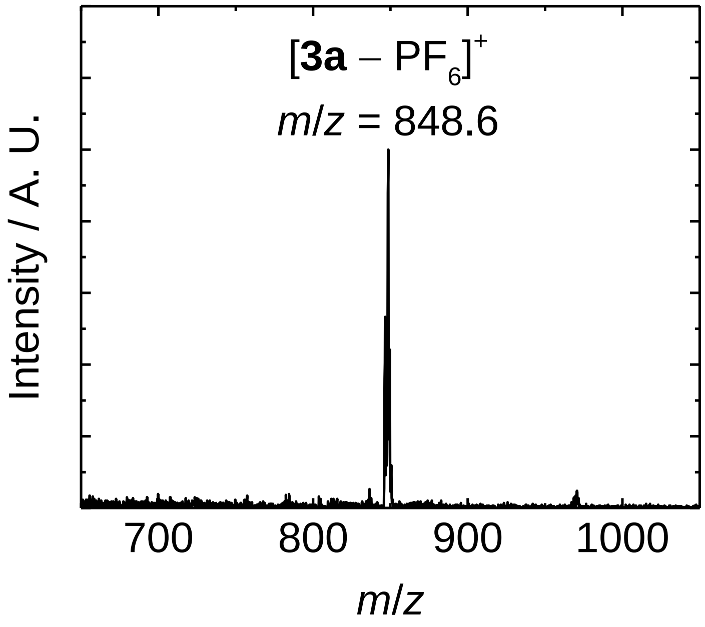

Figure S6. Rate of decay in the absorbance of ABDA (100 μM) at ≈ 378 nm in aerated McIlvaine buffer (pH 3.0, 5.0, and 7.4)/CH_3CN (4:1, *v*/*v*) in the presence of complex **3a** or **3b** upon irradiation at 355 nm. A solution of ABDA without any sensitizers was used as the negative control.

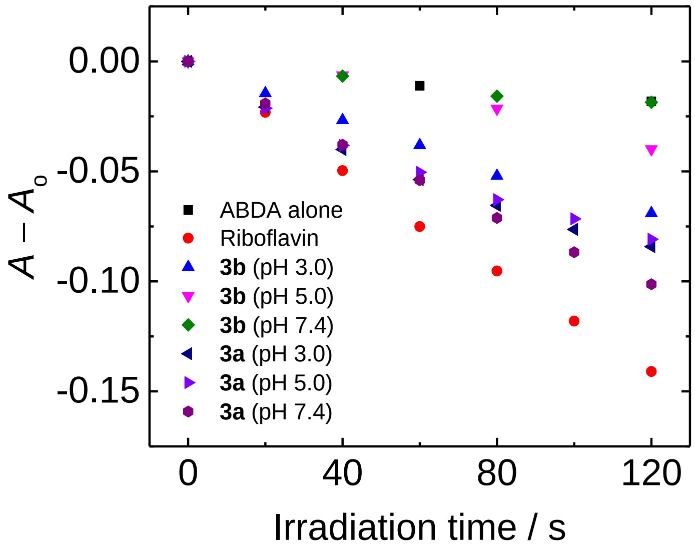

Figure S7. HPLC chromatograms of the reaction mixtures of complexes **1a** – **3a** (20 μM) without (black) or with ICPr-coum (25 μM) (red) in aerated (a) McIlvaine buffer (pH 7.4)/DMSO (4:1, *v*/*v*) and (b) McIlvaine buffer (pH 5.0)/DMSO (4:1, *v*/*v*) at 37°C for 18 h. The absorbance was monitored at 325 nm.[a]

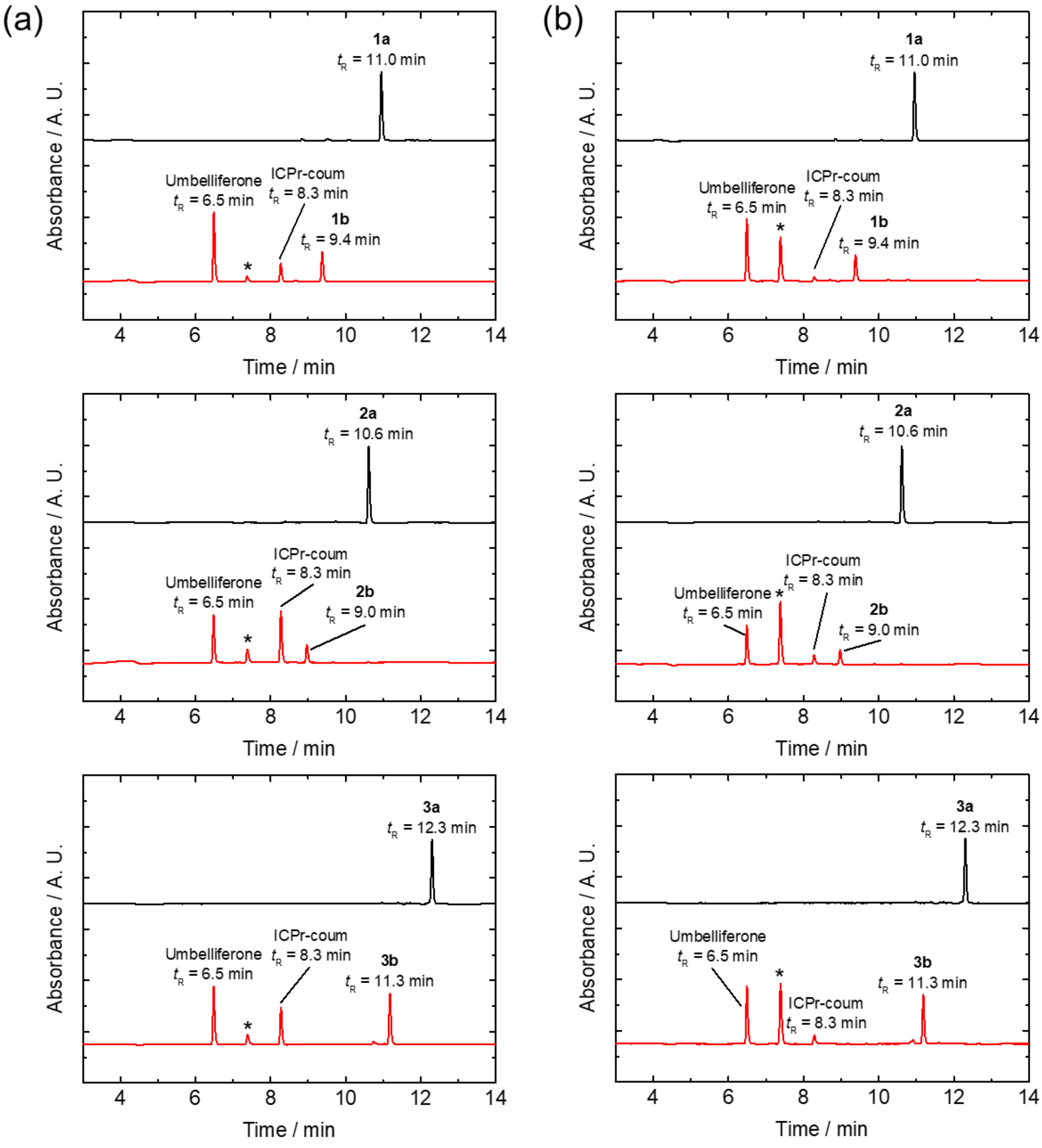

[a] The asterisk (*) denotes an intermediate species (t_R = 7.5 min) of ICPr-coum formed prior to the complete release of the product, umbelliferone.

Figure S8. Second-order kinetics for the reaction of complexes **1a** – **3a** (20 μM) with ICPr-coum (25 μM) at different time points in aerated (a) McIlvaine buffer (pH 7.4)/DMSO (4:1, *v*/*v*) and (b) McIlvaine buffer (pH 5.0)/DMSO (4:1, *v*/*v*) after incubation at 37°C. The slope of the linear fit corresponds to the k_2 value of the reaction.

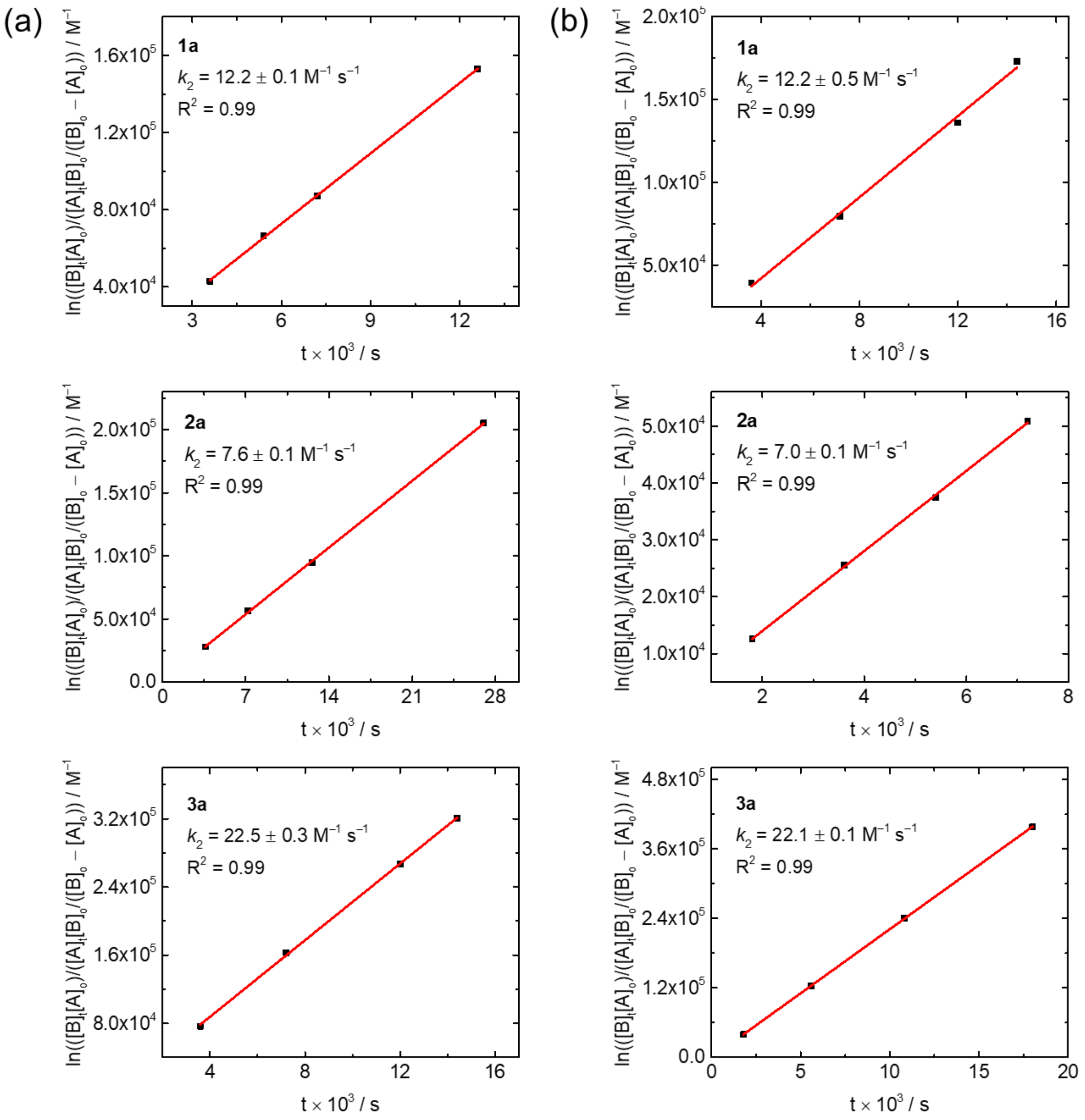

Figure S9. HPLC chromatograms of the reaction mixtures of complexes **1a** – **3a** (50 µM) without (black) or with ICPr-coum (20 µM) (red) in aerated (a) McIlvaine buffer (pH 7.4)/DMSO (4:1, *v*/*v*) and (b) McIlvaine buffer (pH 5.0)/DMSO (4:1, *v*/*v*) at 37°C for 18 h. The absorbance was monitored at 325 nm.[a]

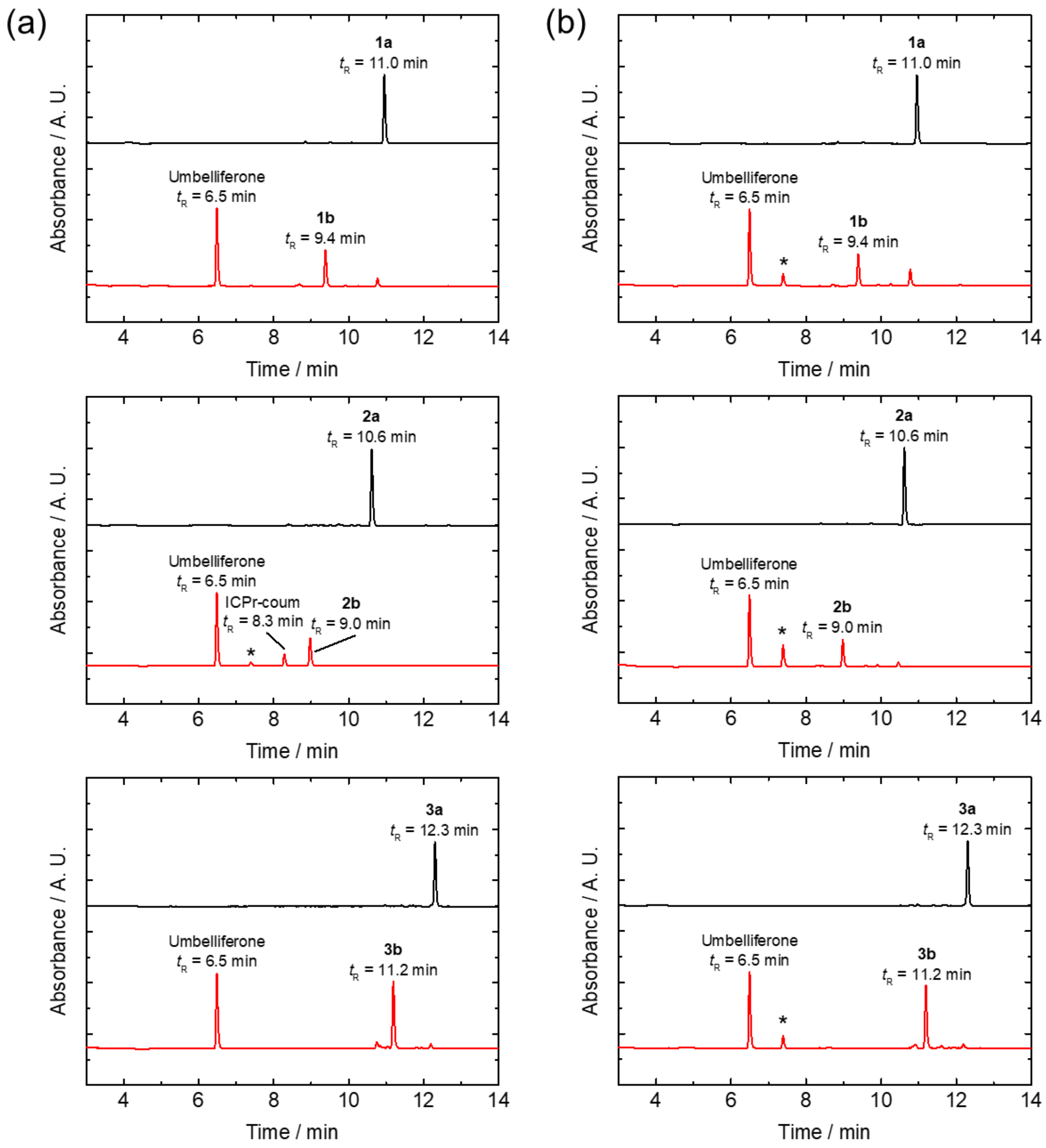

[a] The asterisk (*) denotes an intermediate species (t_R = 7.5 min) of ICPr-coum formed prior to the complete release of the product, umbelliferone.

Figure S10. Release profiles of umbelliferone from ICPr-coum (20 μM) after incubation with complexes **1a** – **3a** (50 μM) in aerated (a) McIlvaine buffer (pH 7.4)/DMSO (4:1, *v*/*v*) and (b) McIlvaine buffer (pH 5.0)/DMSO (4:1, *v*/*v*) at 37°C.

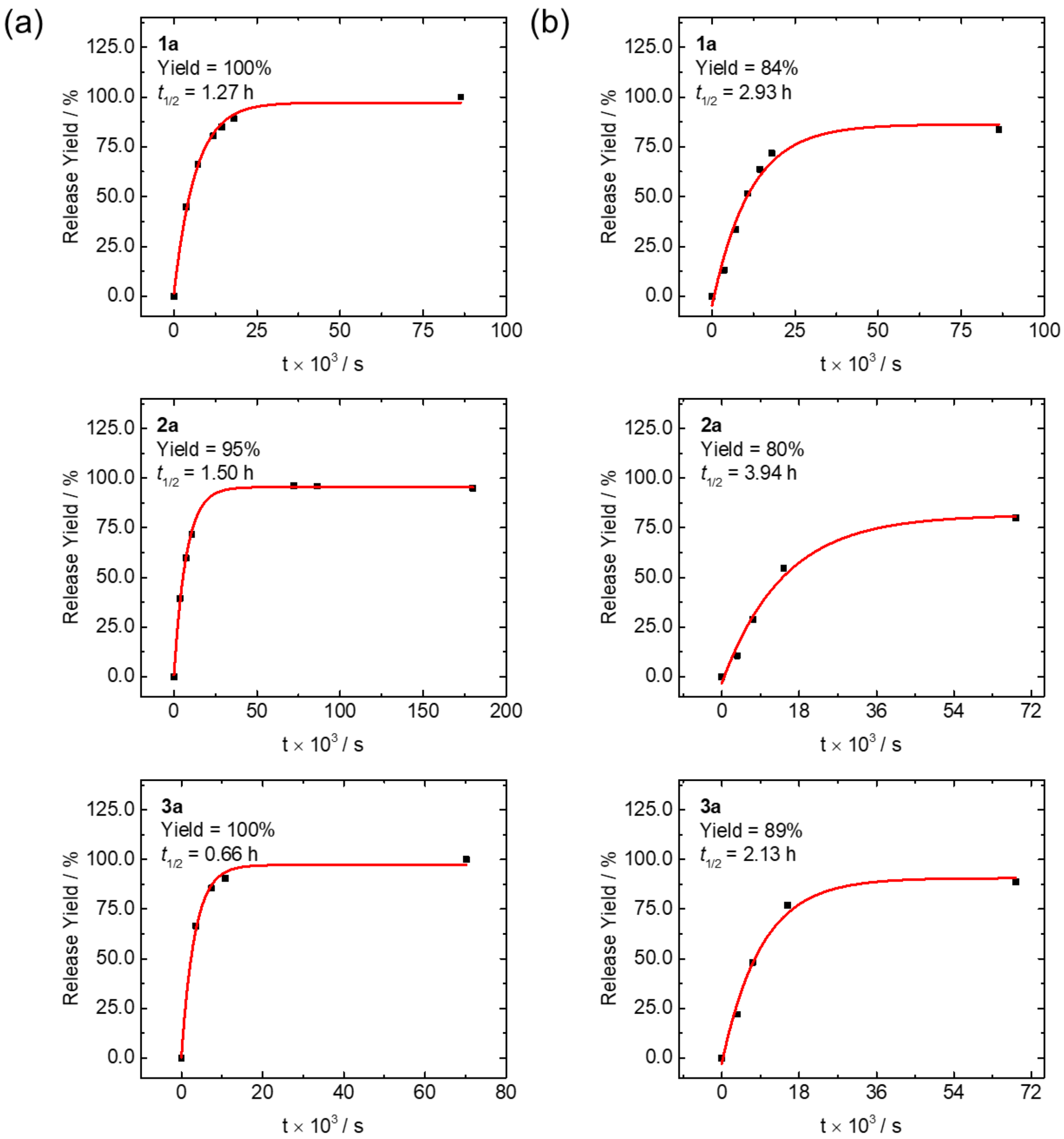

Figure S11. HPLC chromatograms of the reaction mixtures of the ligand py-OCH_2-Tz-^{t}Bu (200 μM) without (black) or with ICPr-coum (250 μM) (red) in aerated (a) McIlvaine buffer (pH 7.4)/CH_3CN (4:1, *v*/*v*) and (b) McIlvaine buffer (pH 5.0)/CH_3CN (4:1, *v*/*v*) at 37°C for 18 h. The absorbance was monitored at 270 and 325 nm.[a]

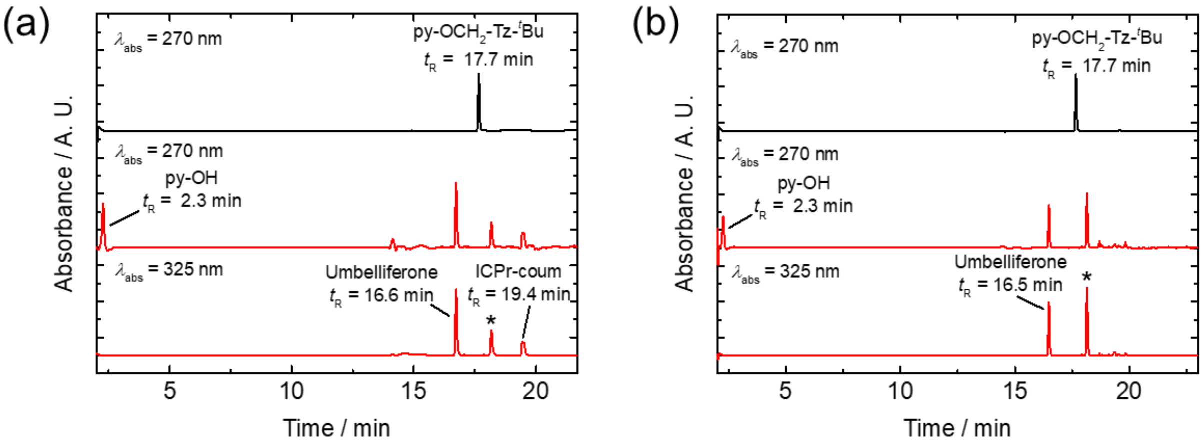

[a] The asterisk (*) denotes an intermediate species (t_R = 18.2 min) of ICPr-coum formed prior to the complete release of the product, umbelliferone.

Figure S12. Second-order kinetics for the reaction of the ligand py-OCH_2-Tz-^{t}Bu (200 μM) with ICPr-coum (250 μM) at different time points in aerated (a) McIlvaine buffer (pH 7.4)/CH_3CN (4:1, *v*/*v*) and (b) McIlvaine buffer (pH 5.0)/CH_3CN (4:1, *v*/*v*) after incubation at 37°C. The slope of the linear fit corresponds to the k_2 value of the reaction.

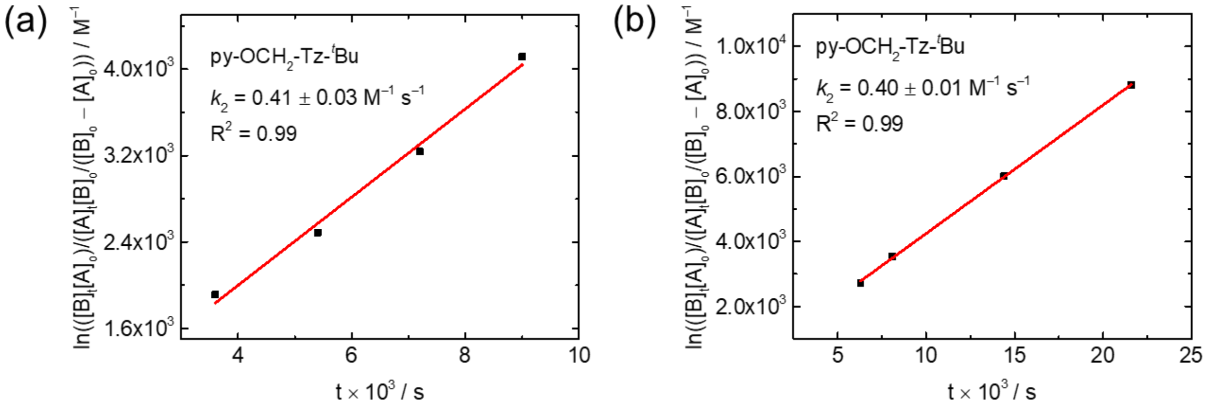

Figure S13. HPLC chromatograms of the reaction mixtures of the ligand py-OCH_2-Tz-^{t}Bu (200 μM) without (black) or with ICPr-coum (80 μM) (red) in aerated (a) McIlvaine buffer (pH 7.4)/CH_3CN (4:1, *v*/*v*) and (b) McIlvaine buffer (pH 5.0)/CH_3CN (4:1, *v*/*v*) at 37°C for 18 h. The absorbance was monitored at 270 and 325 nm.[a]

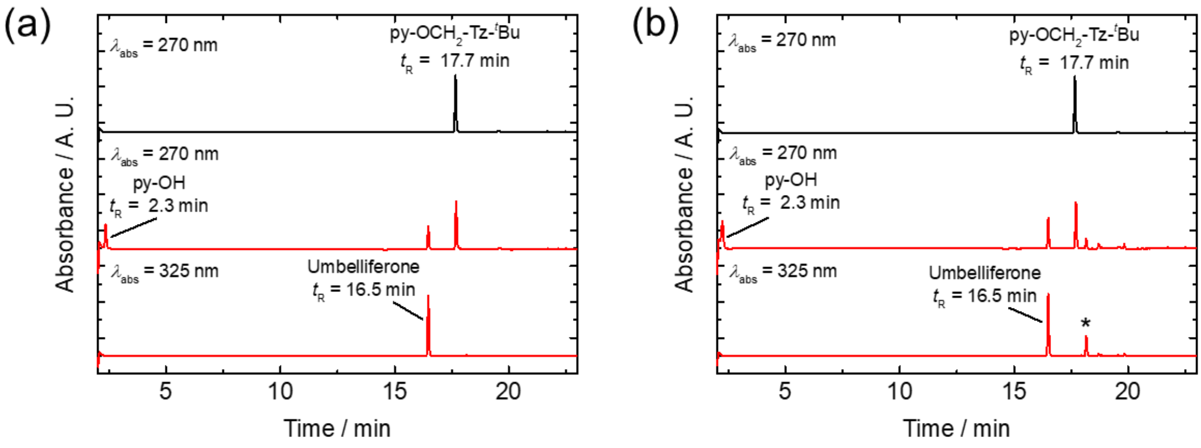

[a] The asterisk (*) denotes an intermediate species (t_R = 18.2 min) of ICPr-coum formed prior to the complete release of the product, umbelliferone.

Figure S14. Release profiles of umbelliferone from ICPr-coum (80 μM) after incubation with the ligand py-OCH_2-Tz-^{t}Bu (200 μM) in aerated (a) McIlvaine buffer (pH 7.4)/CH_3CN (4:1, *v*/*v*) and (b) McIlvaine buffer (pH 5.0)/CH_3CN (4:1, *v*/*v*) at 37°C.

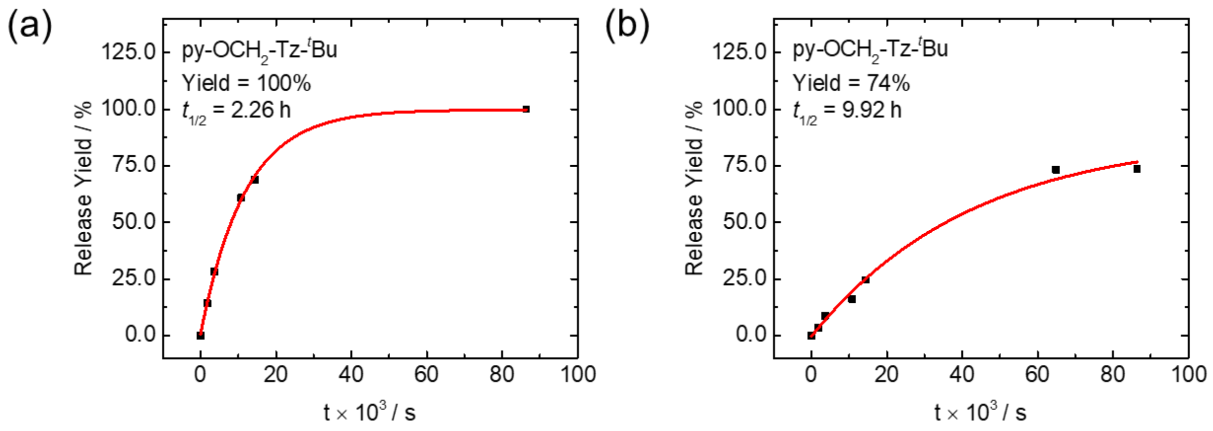

Figure S15. ESI mass spectra of a CH_2Cl_2 extract of lysed HeLa cells that were incubated with complex **3a** (10 μM, 3 h), followed by (a) ICPrc-Bn (200 μM, 4 h) or (b) fresh DMEM (4 h) at 37°C.

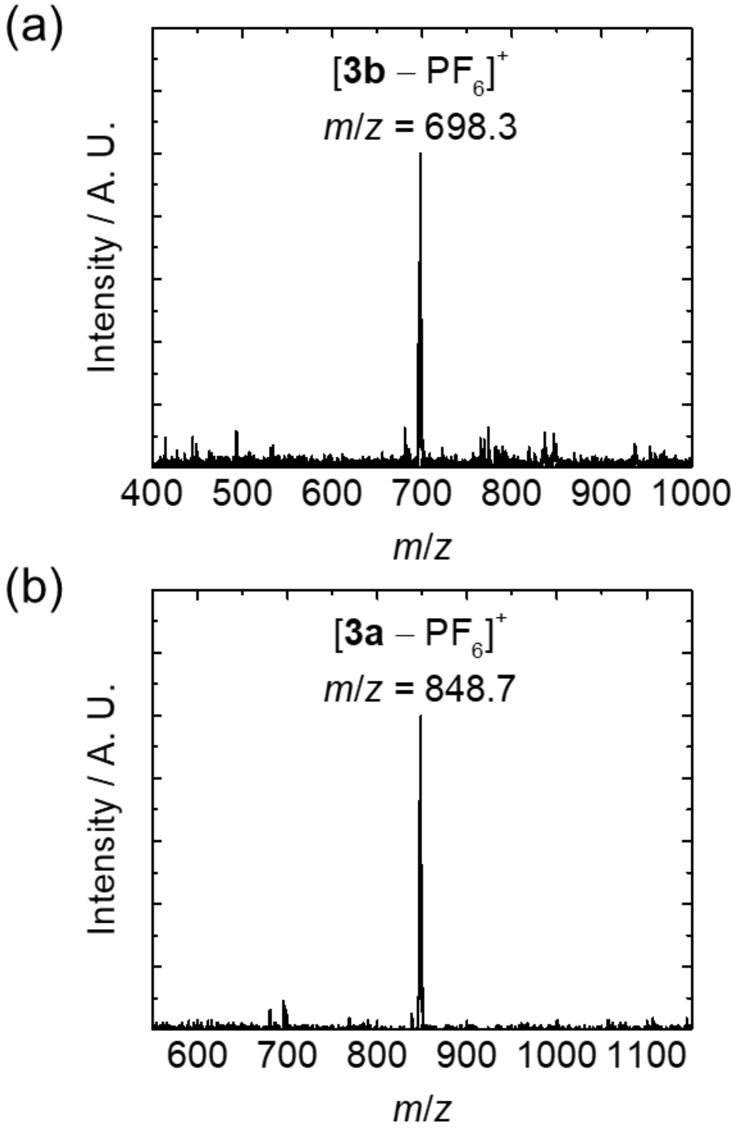

Figure S16. Flow cytometric results of HeLa cells incubated with (a) complex **1a**, (b) complex **2a**, and (c) complex **3a** under the following conditions: (i) blank medium for 4 h (red), (ii) the complex (10 μM) for 3 h, followed by fresh medium for 4 h (green), and (iii) the complex (10 μM) for 3 h, followed by ICPrc-Bn (200 μM) for 4 h (magenta) at 37°C.

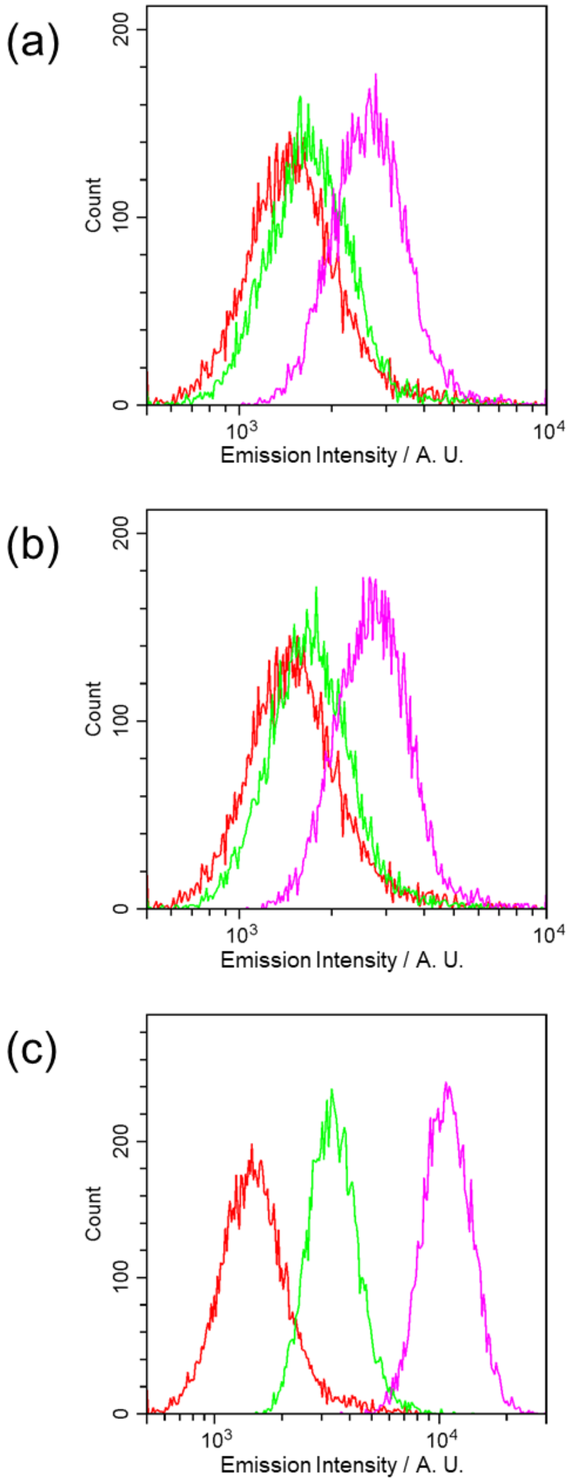

Figure S17. LSCM images of HeLa cells incubated with complexes **1a** – **3a** (10 μM, 3 h, λ_{ex} = 405 nm, λ_{em} = 500 – 600 nm for complexes **1a** and **2a** and 550 − 600 nm for complex **3a**) and ICPrc-Bn (200 μM, 4 h), and further incubated with MitoTracker Deep Red (100 nM, 20 min, λ_{ex} = 635 nm, λ_{em} = 650 − 670 nm) at 37°C. PCC = 0.21 (complex **1a**), 0.17 (complex **2a**), and 0.56 (complex **3a**). Scale bar = 25 μm.

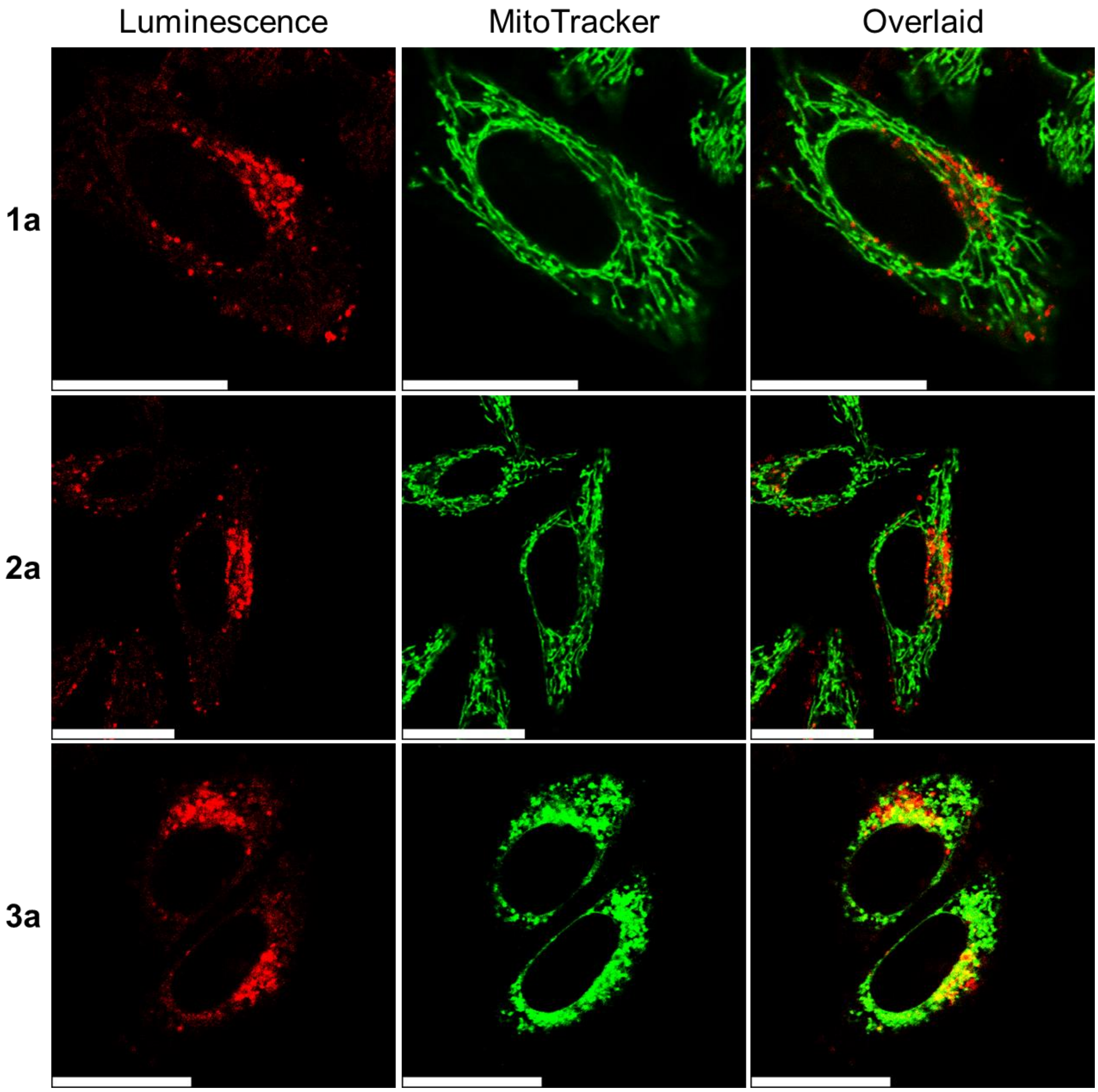

Figure S18. Relative amounts of rhenium associated with an average HeLa cell upon incubation with complex **3a** (10 μM, 1 h) at 37°C without or with preincubation of the cells at 4°C for 1 h, EIPA (50 μM, 1.5 h), Me-β-CD (5 mM, 1 h), or chlorpromazine (30 μM, 1 h) at 37°C. The uptake values at 37°C without pretreatment were taken as the reference.

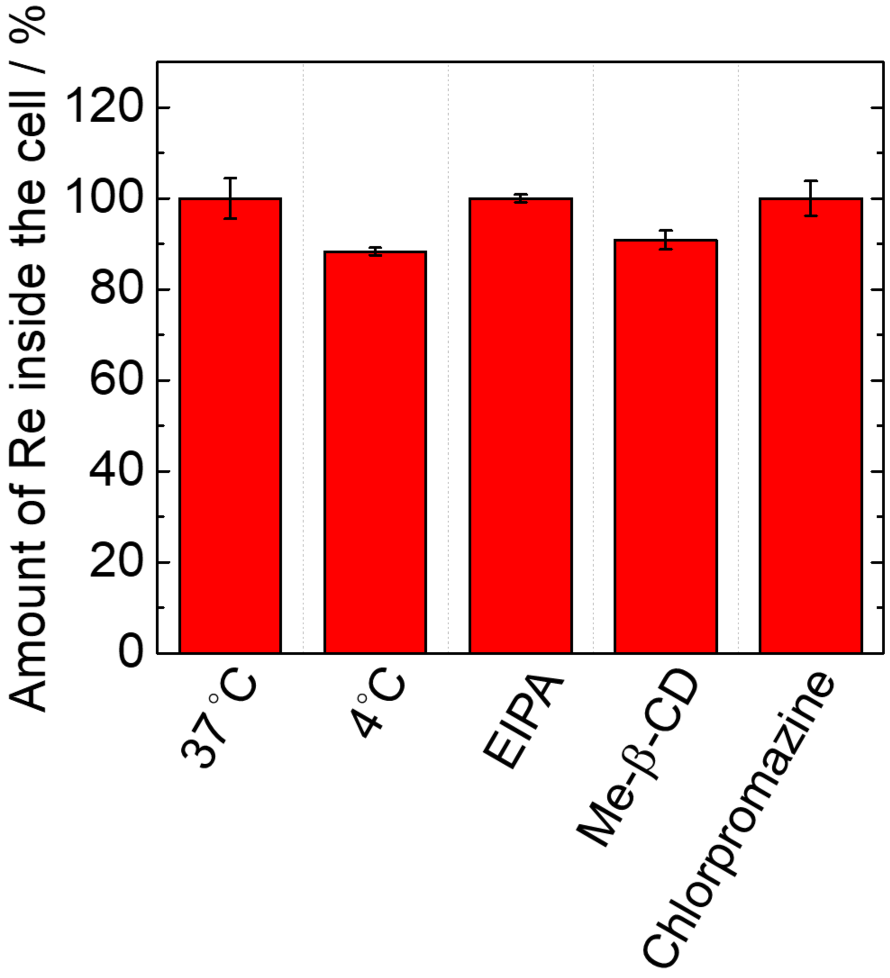

Figure S19. LSCM images of HeLa cells incubated with ICPr-coum (200 μM, 4 h, λ_{ex} = 405 nm, λ_{em} = 430 – 500 nm) alone, or incubated with complex **3a** (10 μM, 3 h, λ_{ex} = 405 nm, λ_{em} = 550 – 600 nm), followed by incubation without or with ICPr-coum (10 μM, 4 h), or incubation with ICPrc-Bn (200 μM, 4 h) at 37°C. PCC = 0.58 (complex **3a** + ICPr-coum). Scale bar = 25 μm.

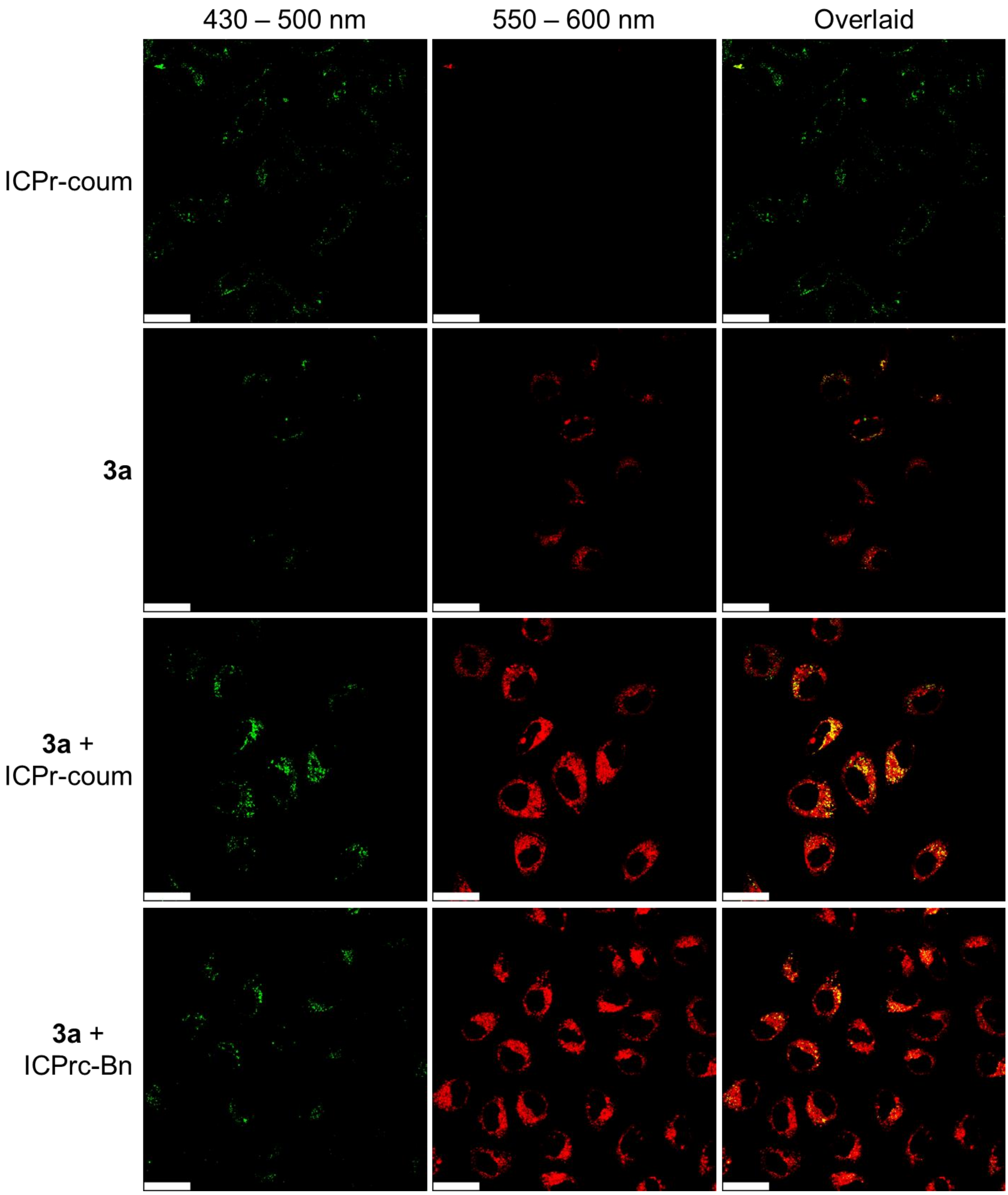

Figure S20. HPLC analysis of the stability of (a) ICPr-fluorescein (25 μM) and (b) ICPrc-Dox (25 μM) in 25% FBS at 37°C in the dark for 48 h. The absorbance was monitored at 250 nm for ICPr-fluorescein and at 480 nm for ICPrc-Dox.

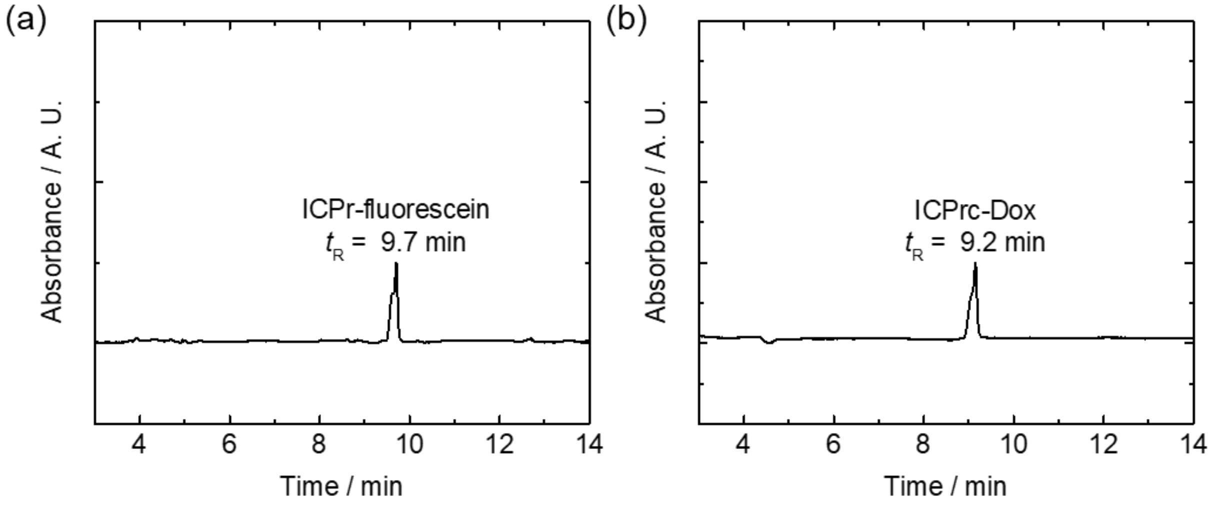

Figure S21. HPLC chromatograms of (a) fluorescein (10 μM) (black) and ICPr-fluorescein (10 μM) (red), and (b) the reaction mixture of complex **3a** (10 μM) without (black) or with ICPr-fluorescein (10 μM) (red) in aerated PBS/DMSO (4:1, *v*/*v*) at 37°C for 4 h and (c) 24 h. The absorbance was monitored at 250 and 325 nm.

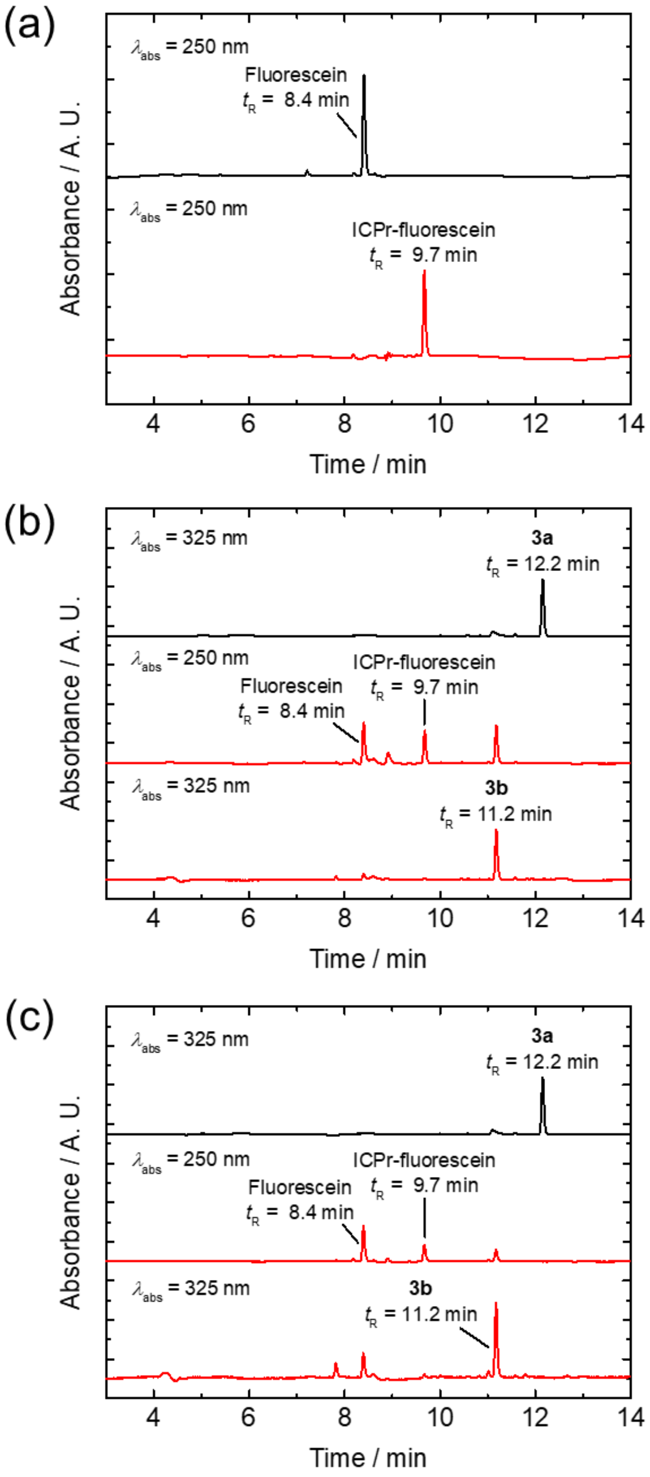

Figure S22. HPLC chromatograms of (a) Dox (10 μM) (black) and ICPrc-Dox (10 μM) (red), and (b) the reaction mixture of complex **3a** (10 μM) without (black) or with ICPrc-Dox (10 μM) (red) in aerated PBS/DMSO (4:1, *v*/*v*) at 37°C for 4 h and (c) 24 h. The absorbance was monitored at 325 and 480 nm.

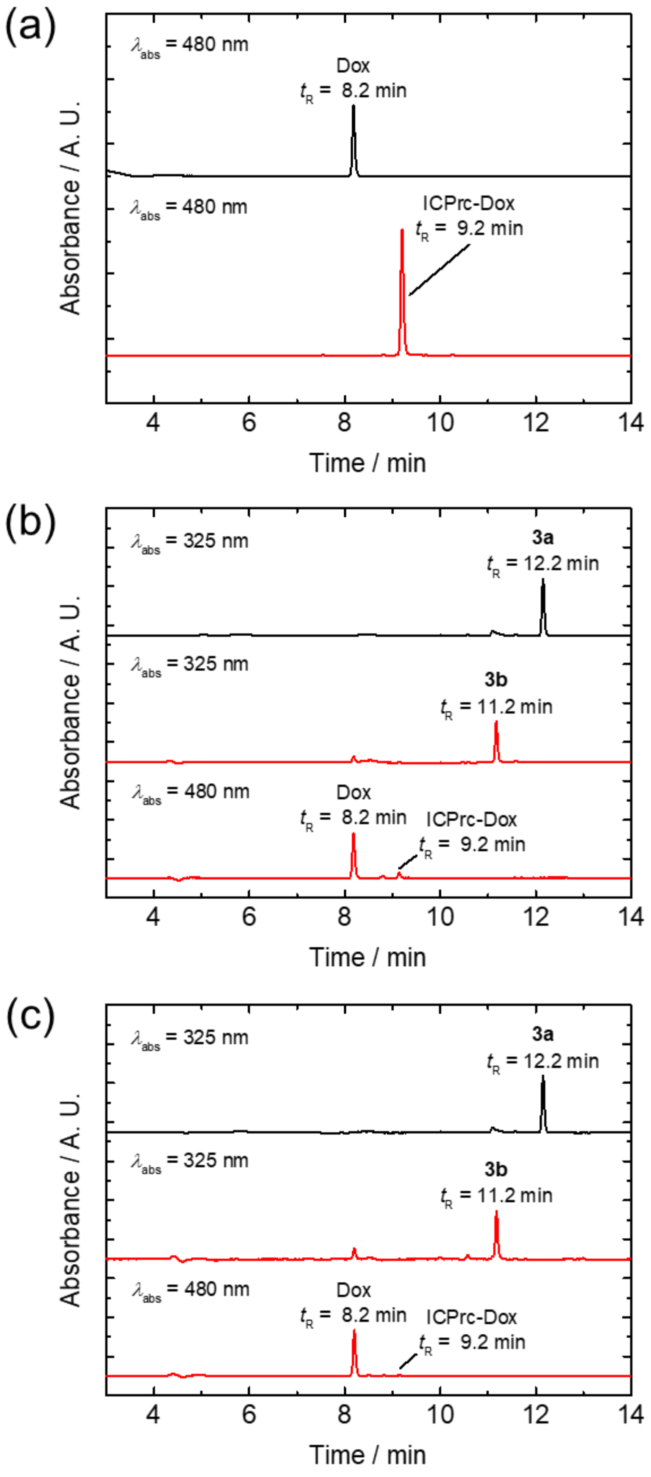

Figure S23. HPLC traces of the reaction mixtures of complex **3a** (50 μM) with (a) ICPr-coum (50 μM), (b) ICPr-fluorescein (50 μM), and (c) ICPrc-Dox (μM) in aerated McIlvaine buffer (pH 7.4)/DMSO (4:1, *v*/*v*) at 37°C, recorded at various time points. The absorbance was monitored at 325 nm for ICPr-coum and at 250 nm for ICPr-fluorescein and ICPrc-Dox. (d) Release profiles of umbelliferone, fluorescein, and Dox from ICPr/ICPrc-caged compounds (50 μM) after incubation with complex **3a** (50 μM) under the same conditions.

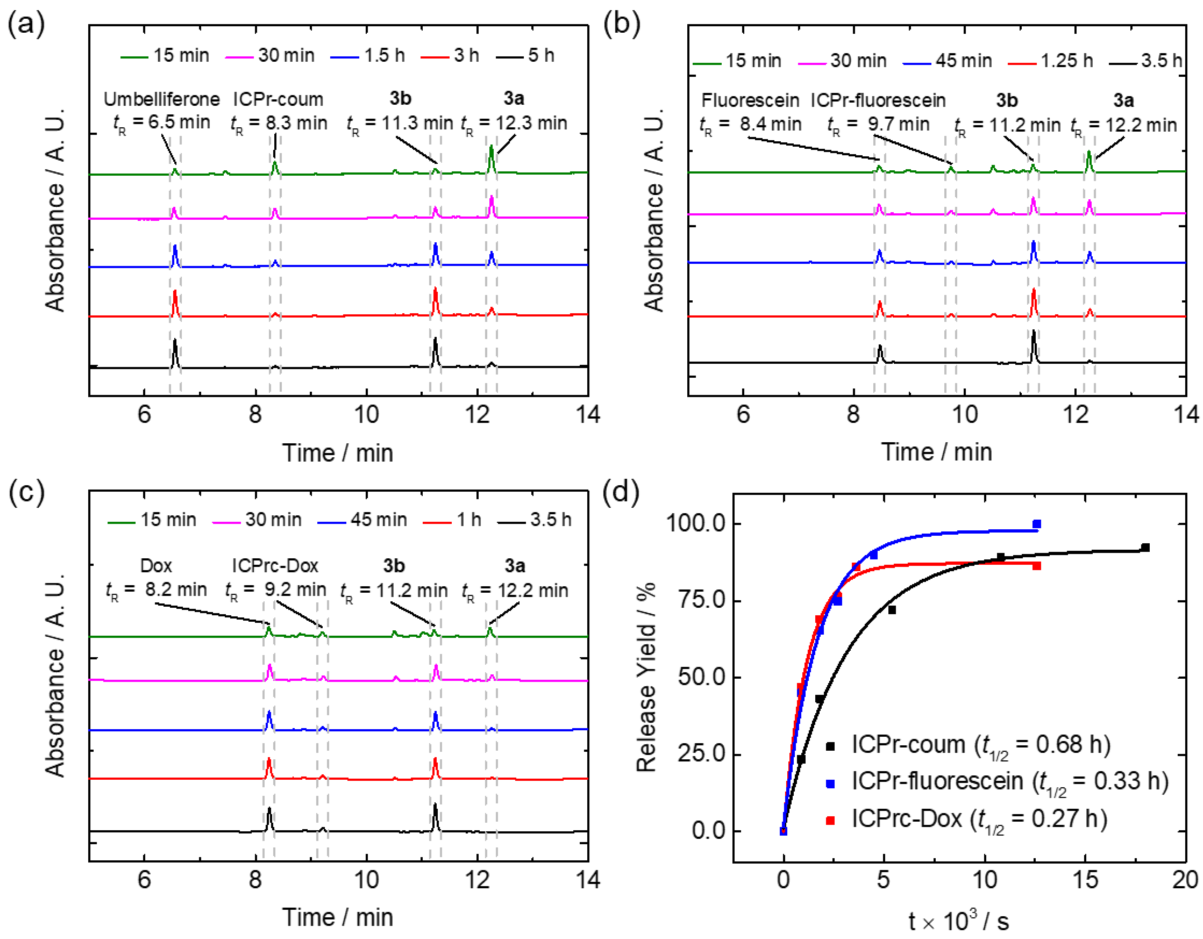

Figure S24. Viability of HEK-293 cells incubated with complex **3a** for 3 h, and then incubated (a) with fresh growth medium, (b) ICPrc-Dox (10 μM), or (c) ICPrc-Dox (50 μM) for 4 h. HeLa cells incubated with (d) Dox for 4 h served as a positive control. The cells were further incubated in the dark (blue) or irradiated at 365 nm (5 mW cm^{-2}) (red) for 5 min, and then incubated with fresh growth medium for 24 h.

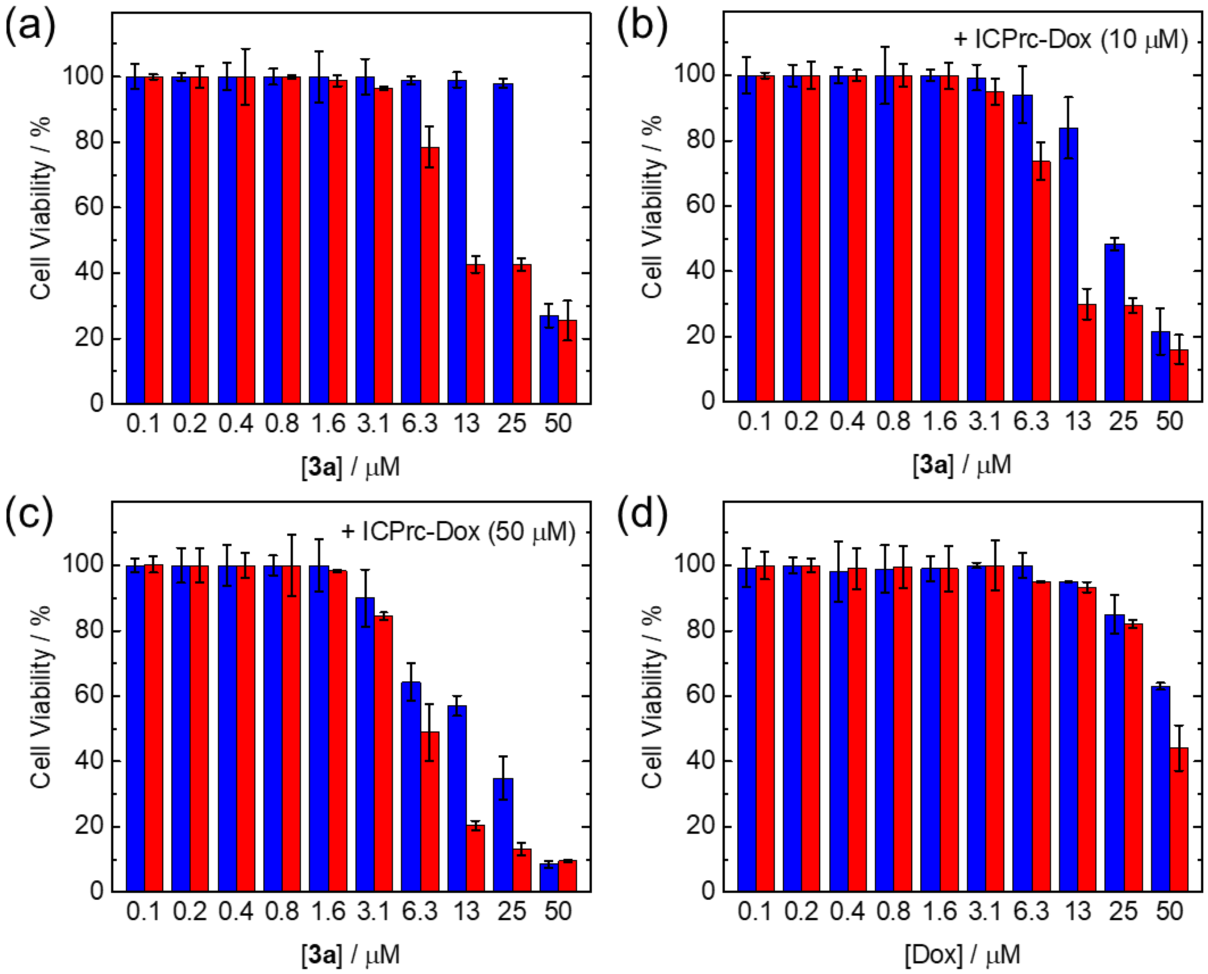

Figure S25. ^{1}H NMR spectrum of py-OCH$_2$-Tz-^{t}Bu in CDCl$_3$ at 298 K.

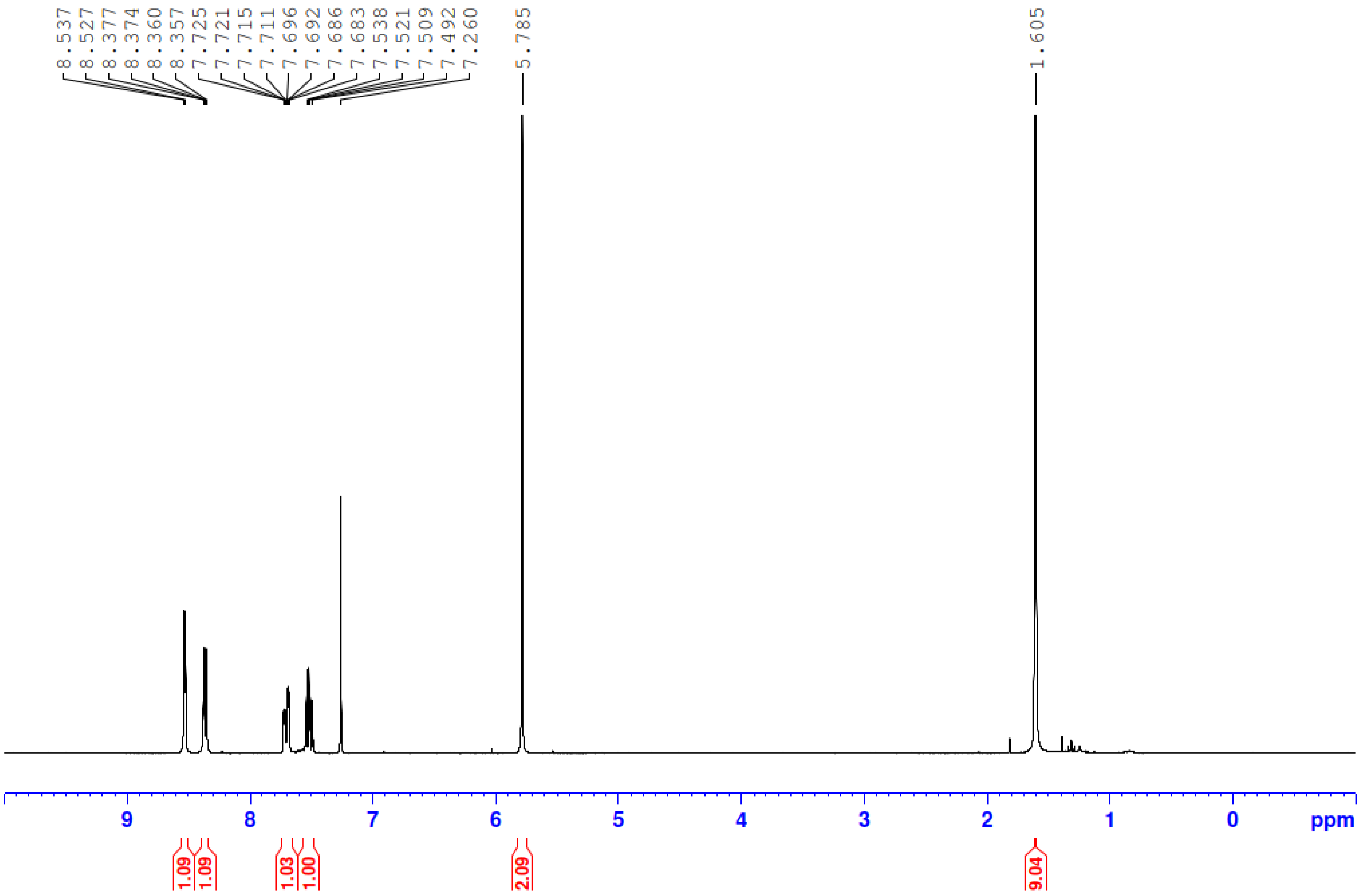

Figure S26. ^{1}H NMR spectrum of complex **1a** in $CDCl_3$ at 298 K.

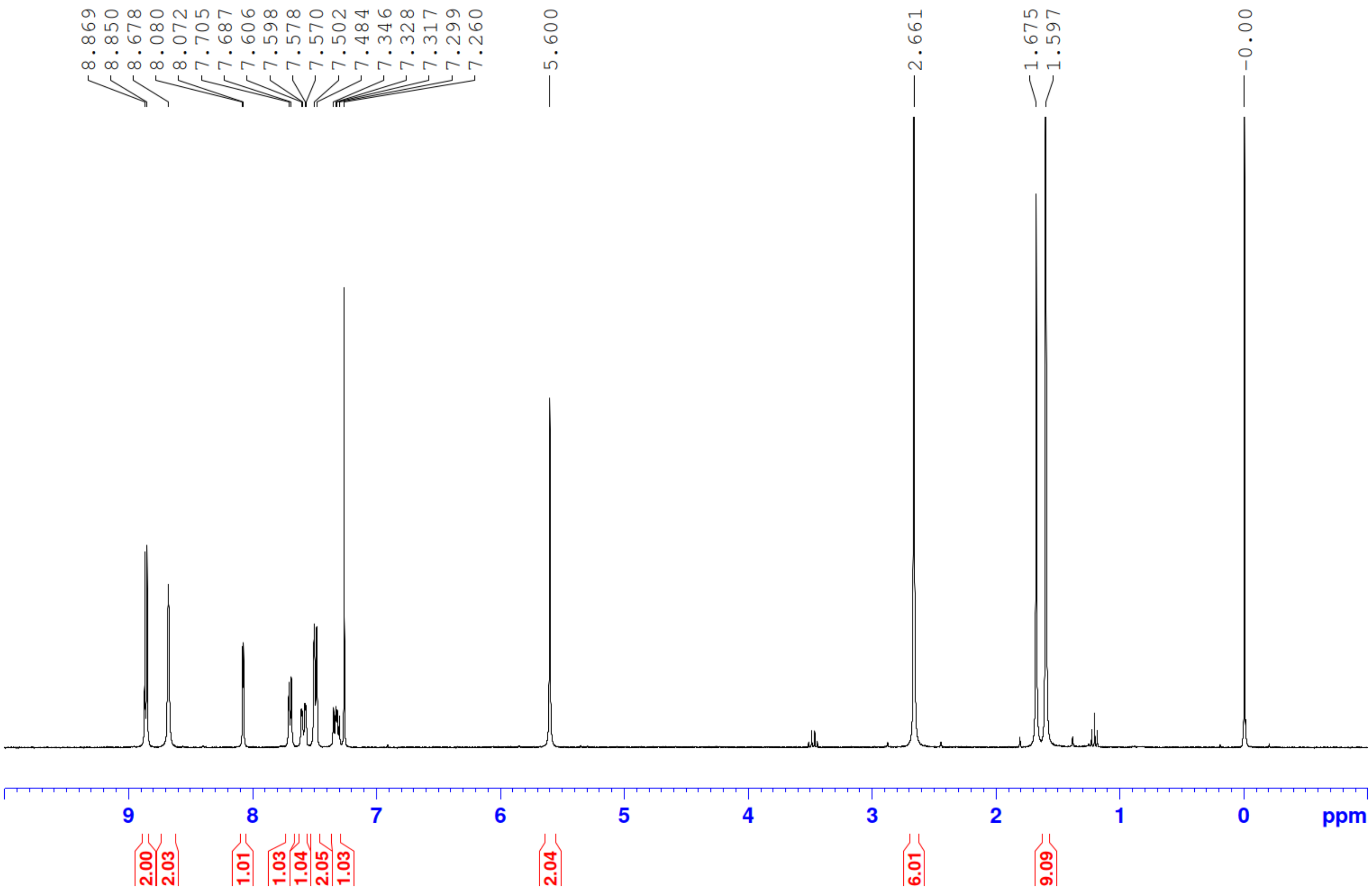

Figure S27. ^{13}C NMR spectrum of complex **1a** in $CDCl_3$ at 298 K.

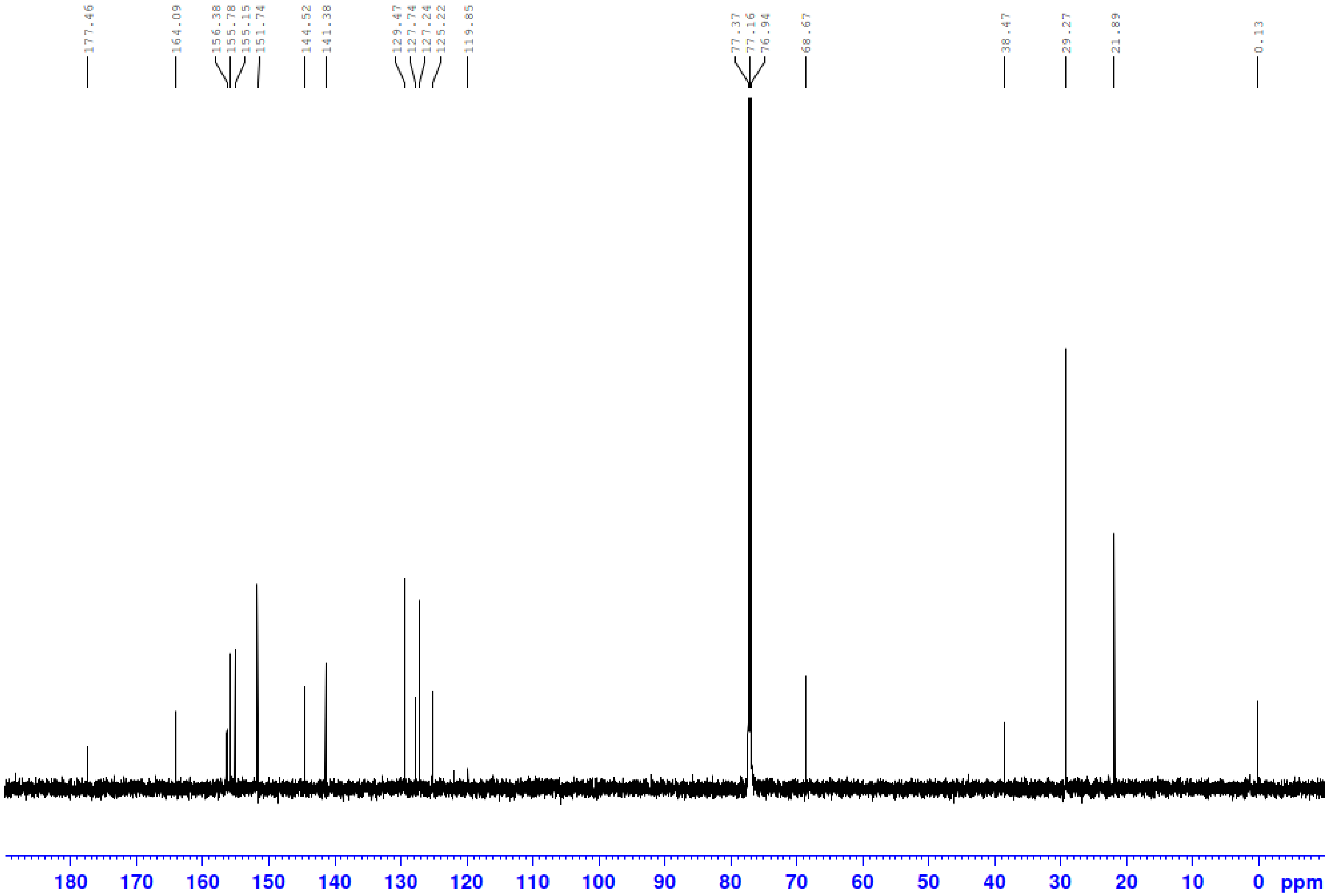

Figure S28. ^{1}H NMR spectrum of complex **1b** in $(CD_3)_2CO$ at 298 K.

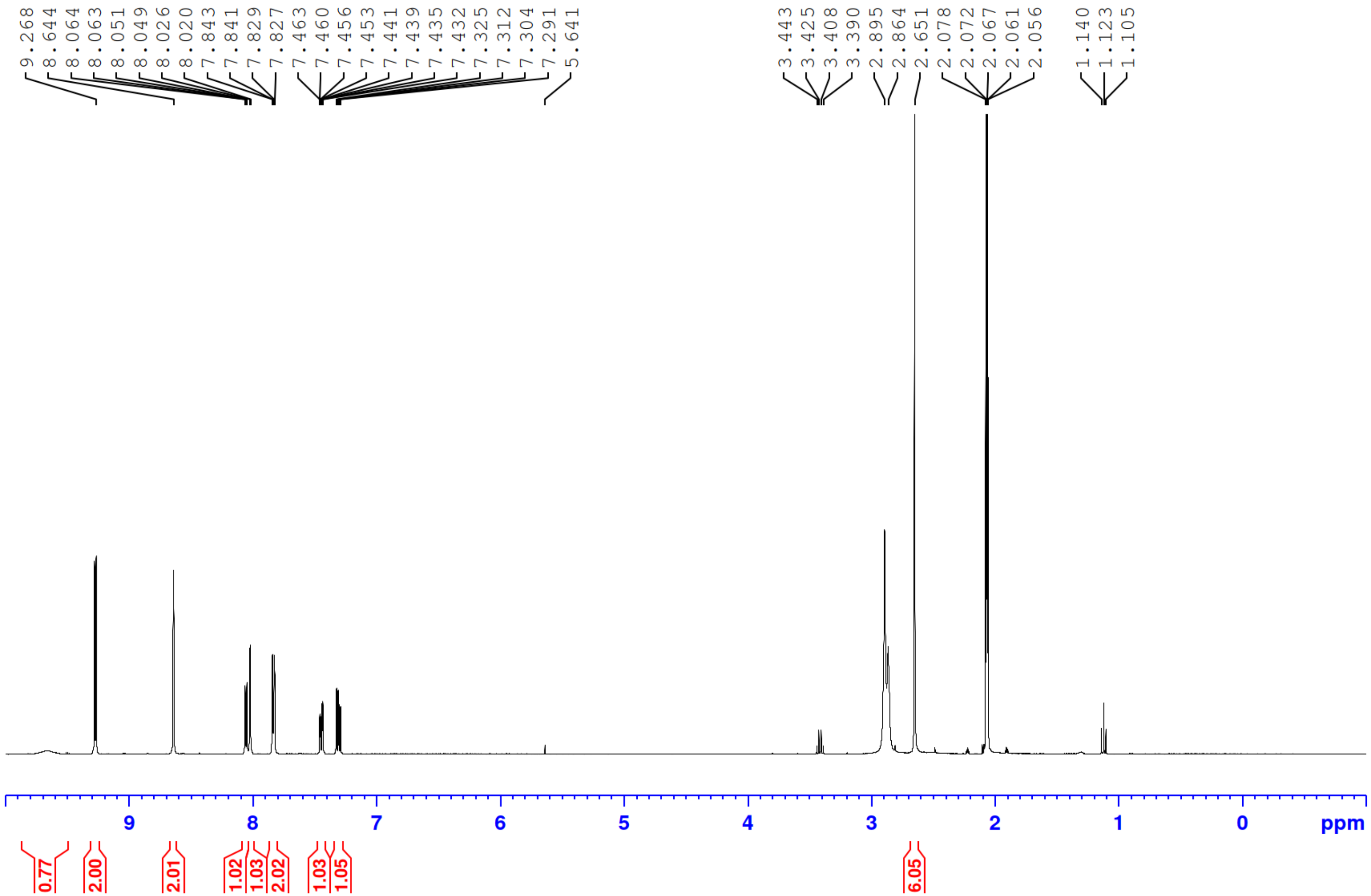

Figure S29. ^{13}C NMR spectrum of complex **1b** in $(CD_3)_2CO$ at 298 K.

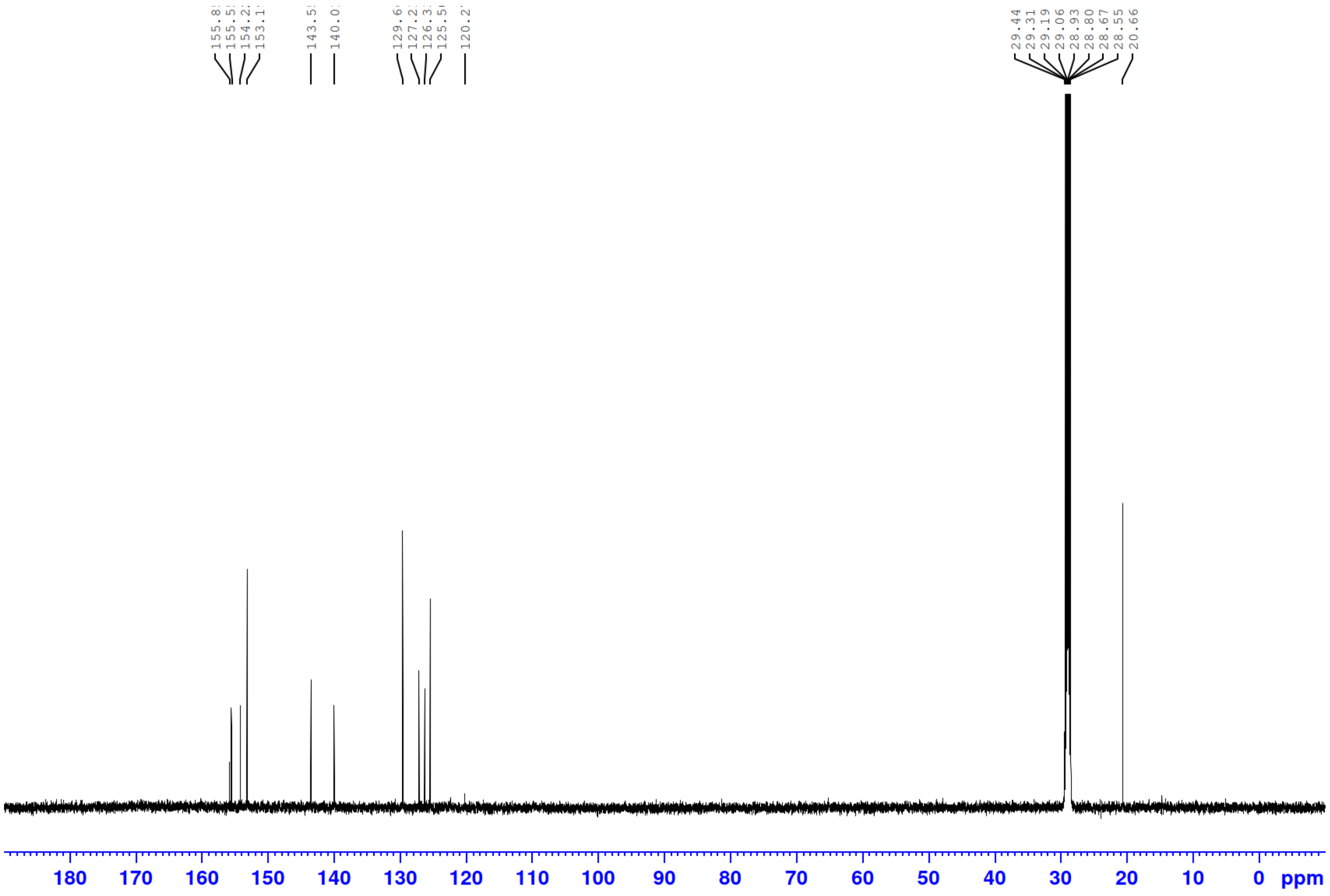

Figure S30. ^{1}H NMR spectrum of complex **2a** in $CDCl_3$ at 298 K.

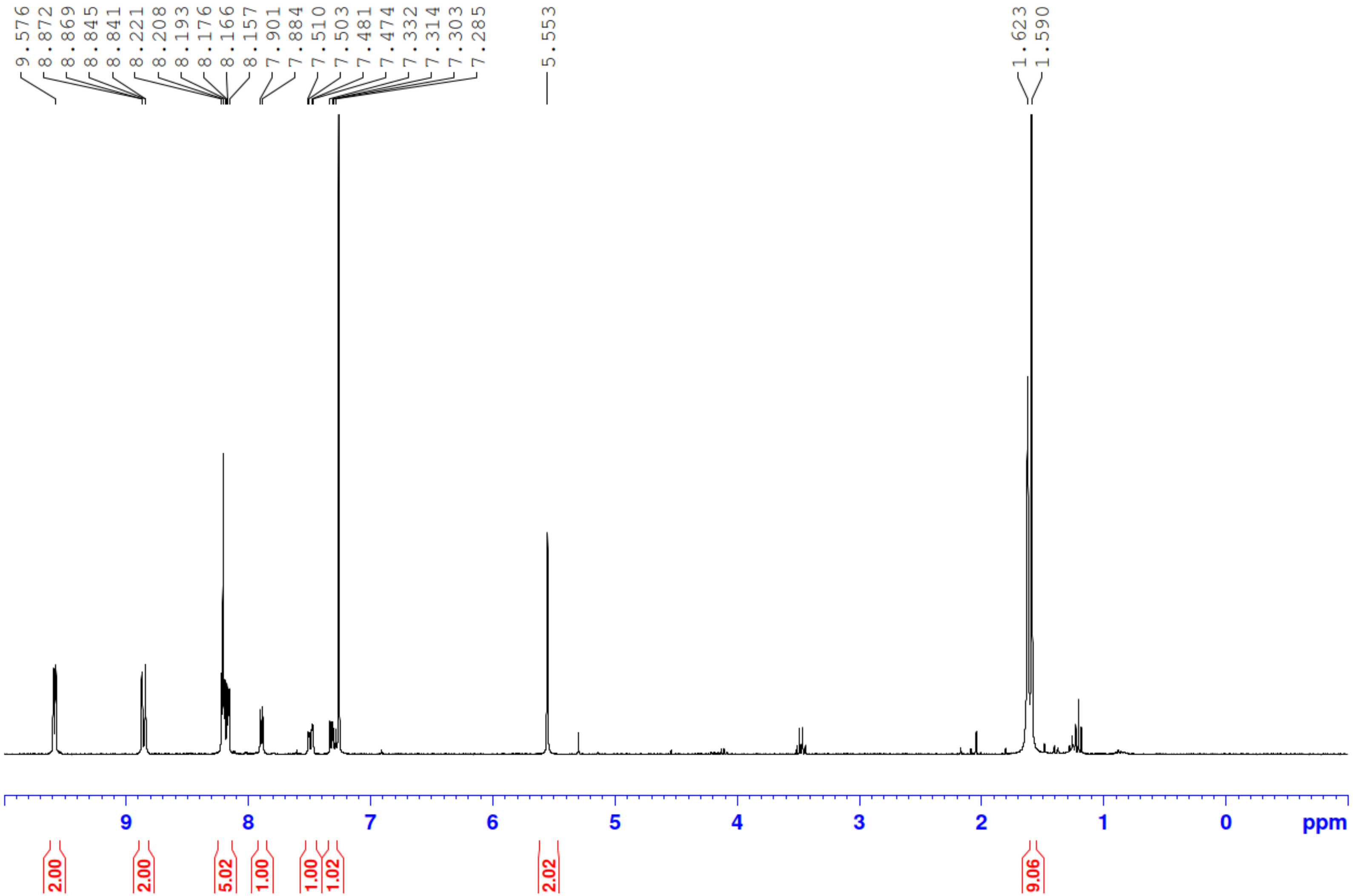

Figure S31. ^{13}C NMR spectrum of complex **2a** in $CDCl_3$ at 298 K.

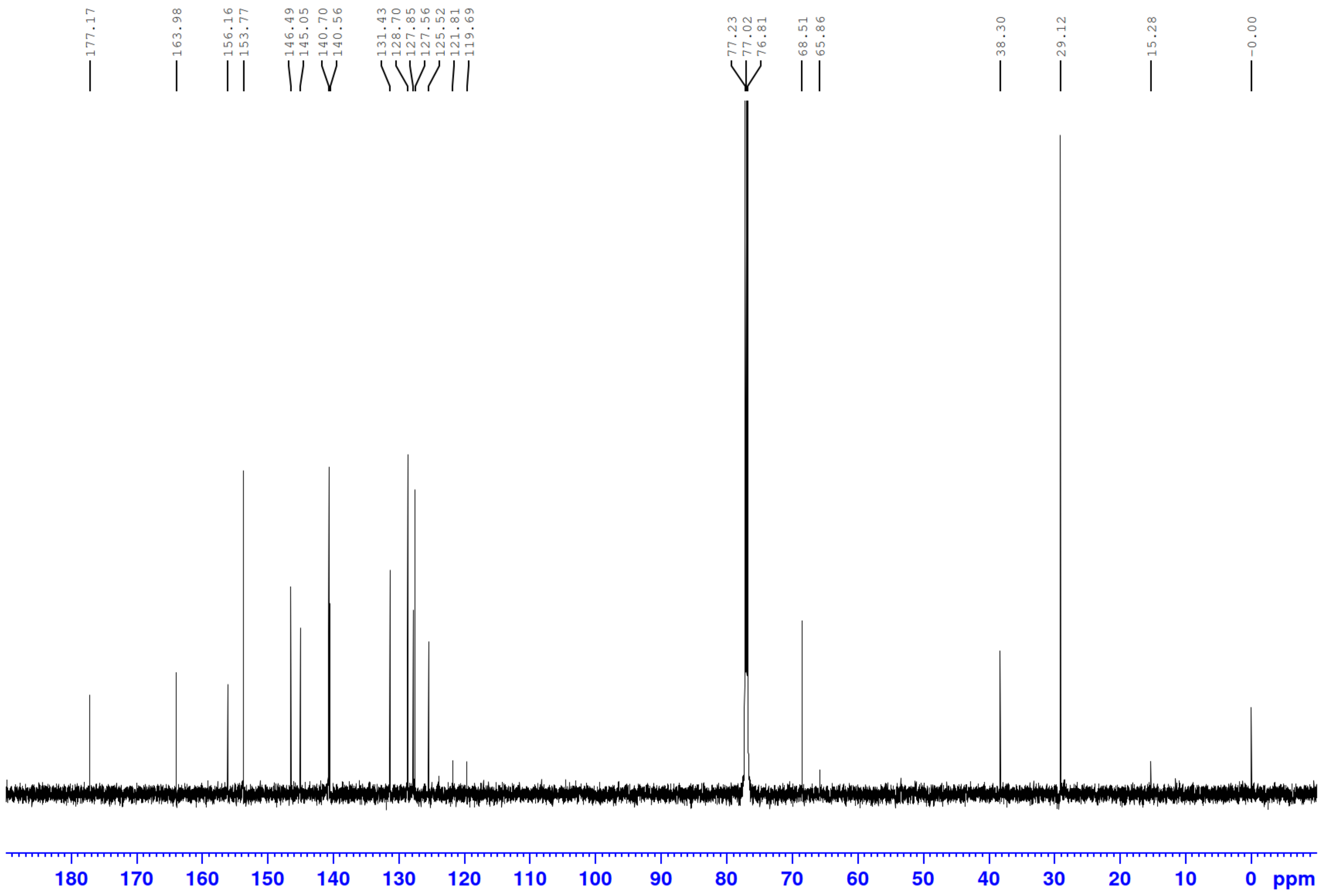

Figure S32. ^{1}H NMR spectrum of complex **2b** in $(CD_3)_2CO$ at 298 K.

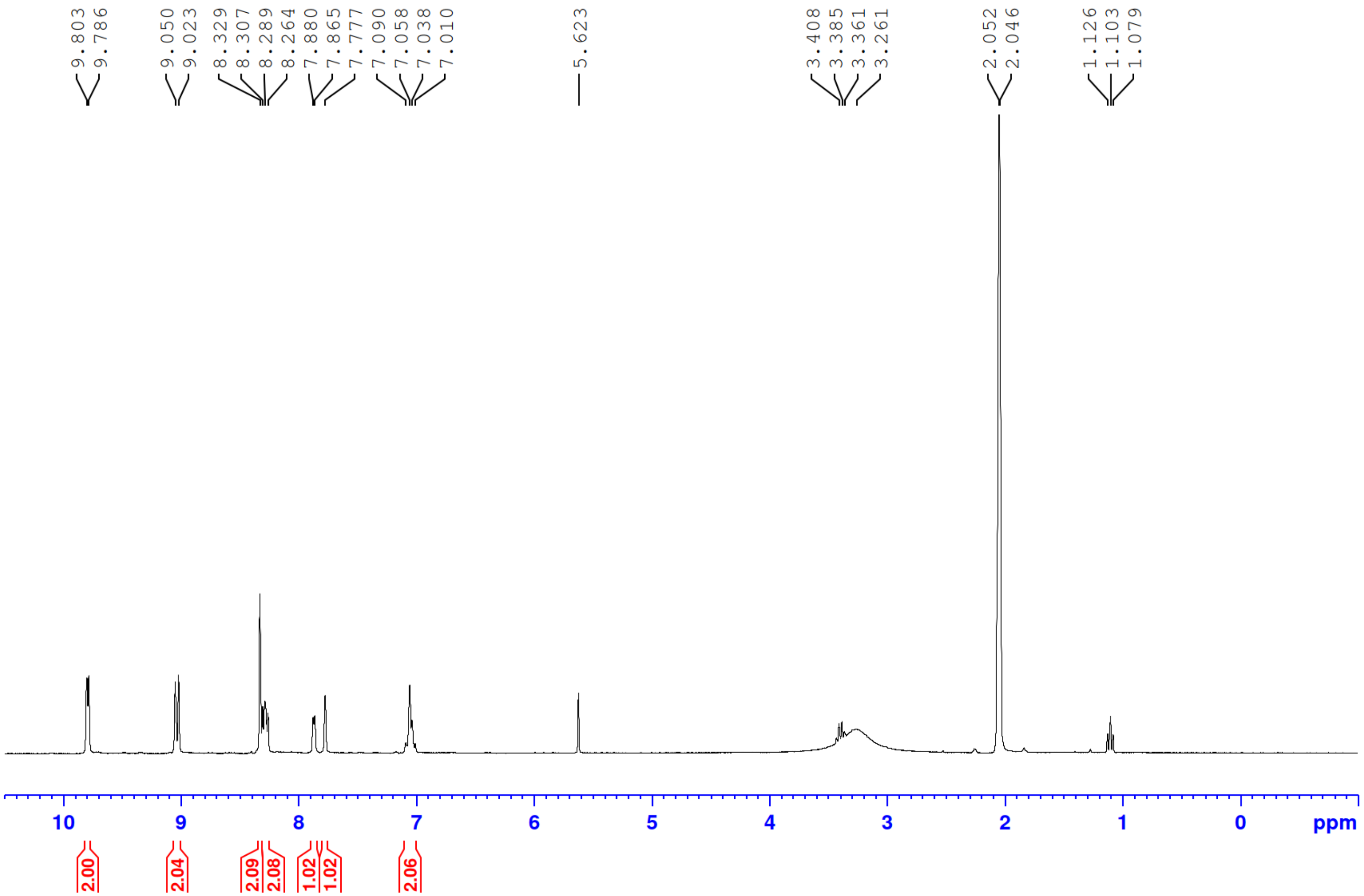

Figure S33. ^{13}C NMR spectrum of complex **2b** in $(CD_3)_2CO$ at 298 K.

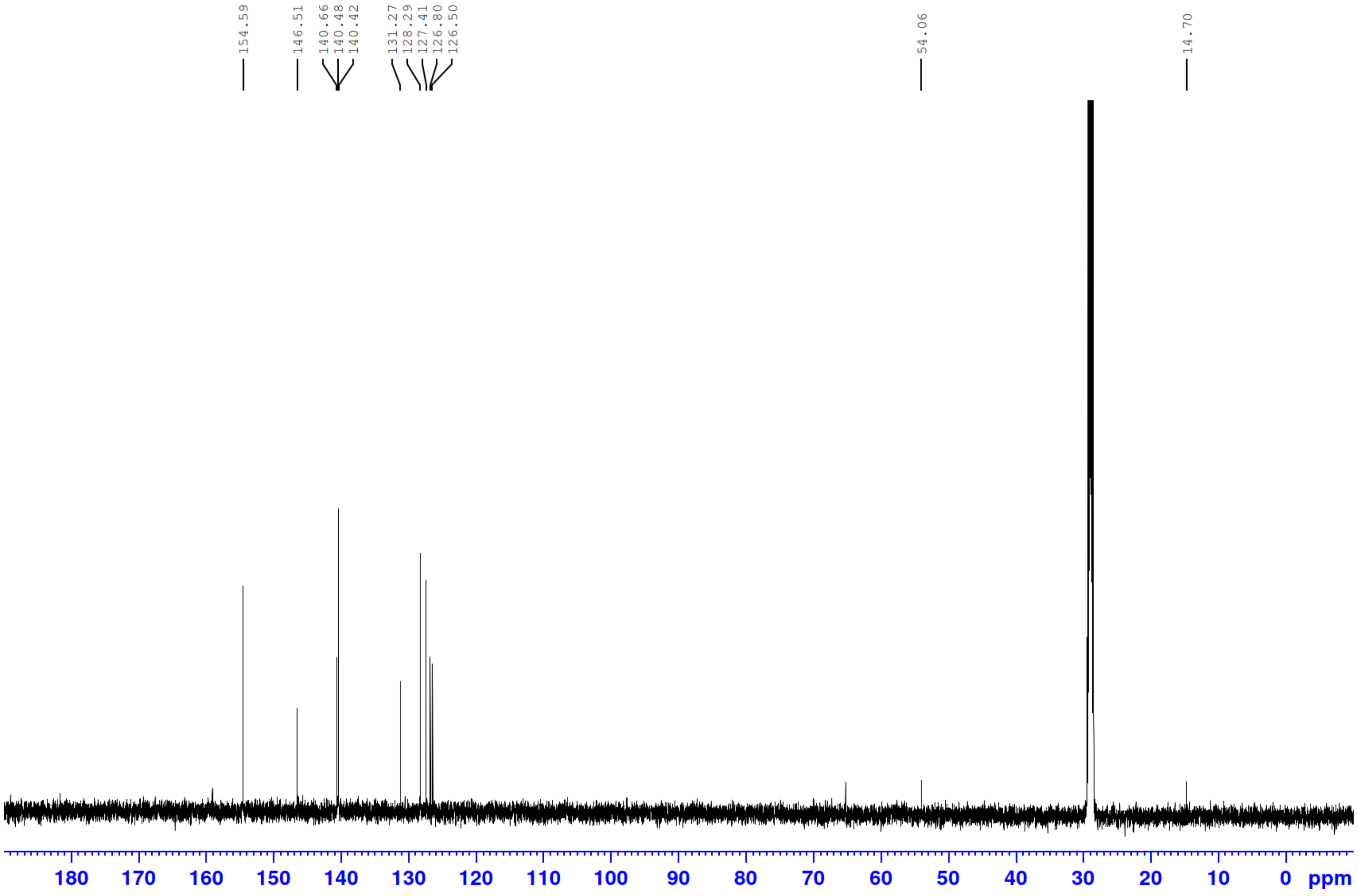

Figure S34. ^{1}H NMR spectrum of complex **3a** in $CDCl_3$ at 298 K.

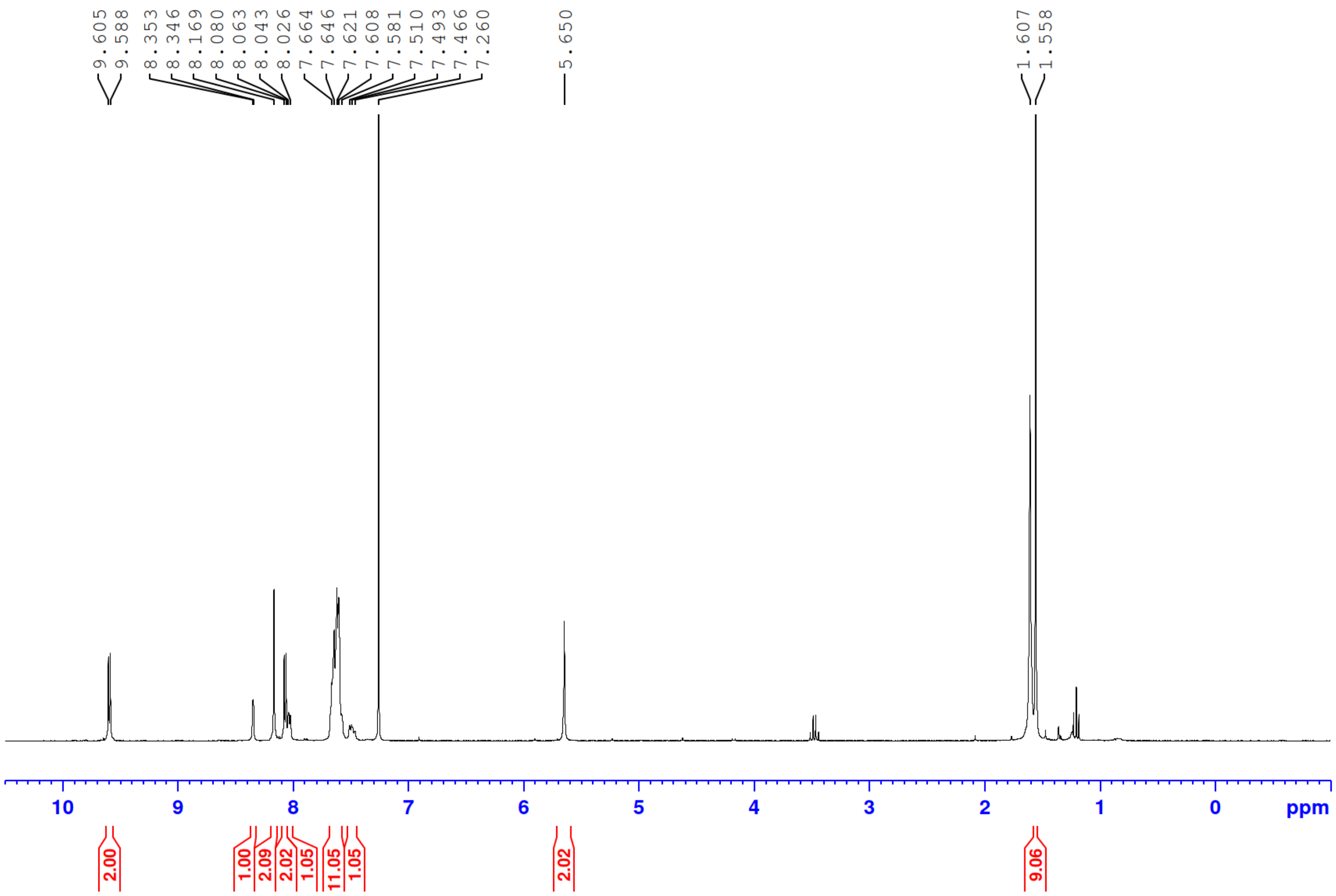

Figure S35. ^{13}C NMR spectrum of complex **3a** in $CDCl_3$ at 298 K.

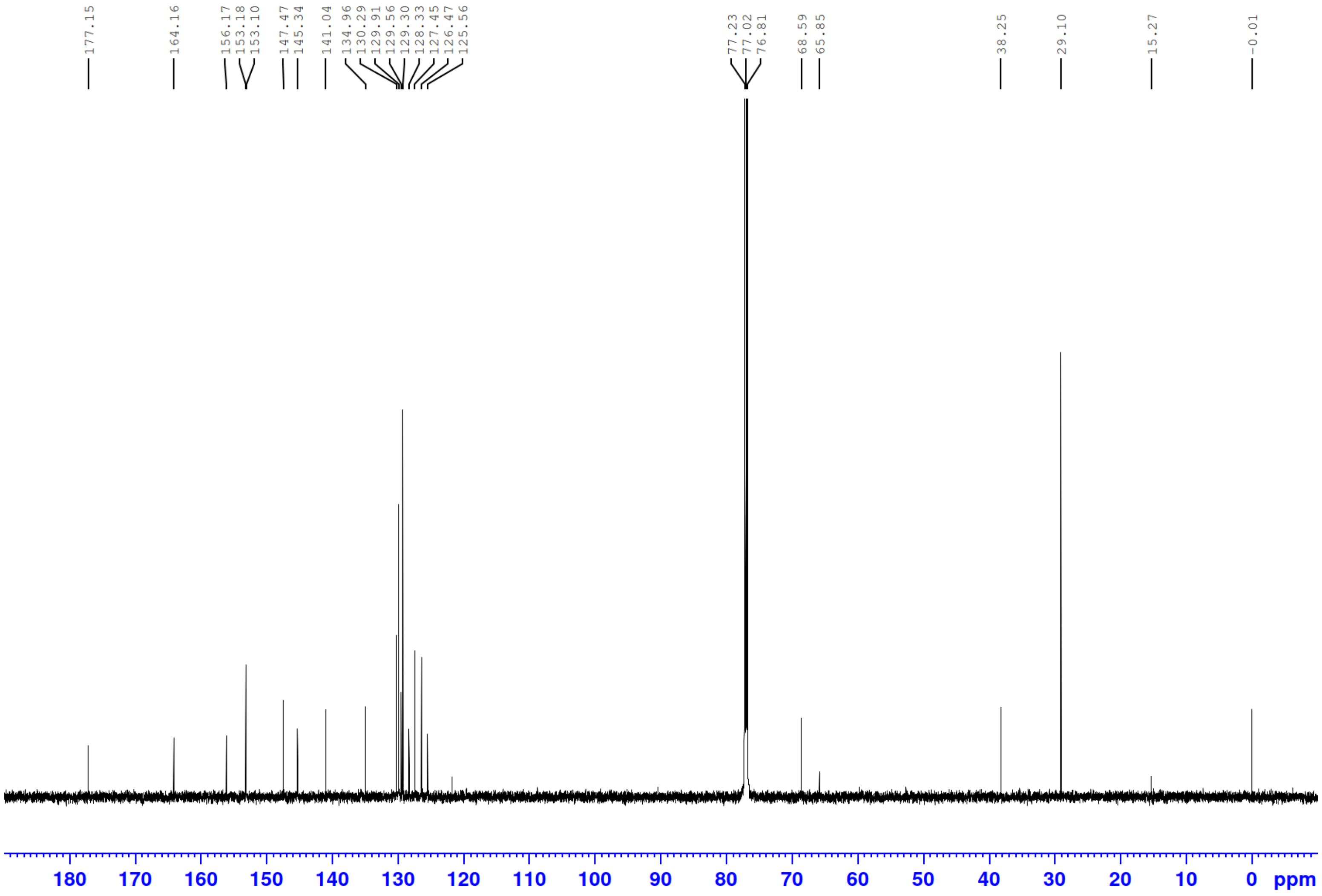

Figure S36. ^{1}H NMR spectrum of complex **3b** in $(CD_3)_2CO$ at 298 K.

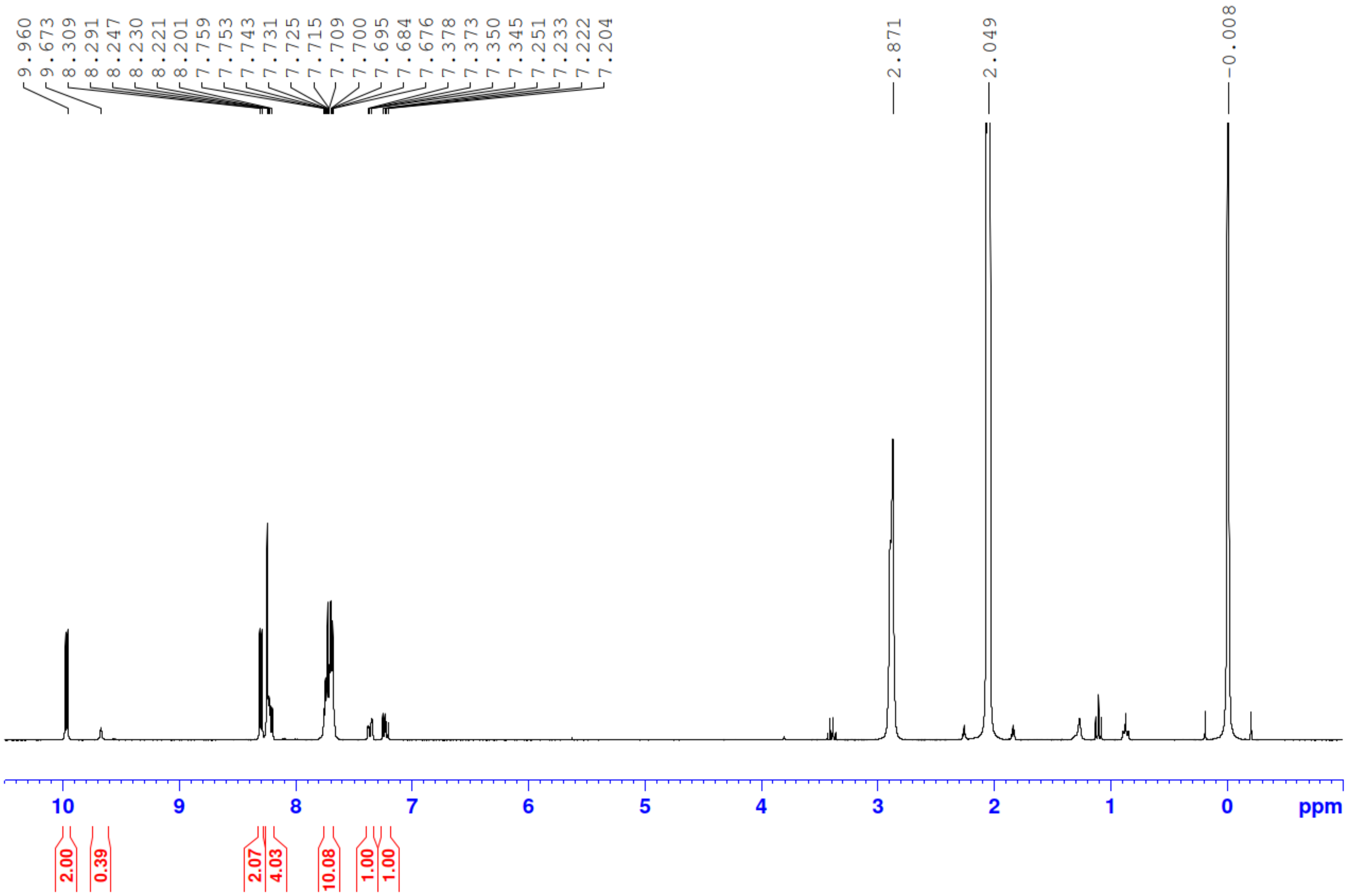

Figure S37. ^{13}C NMR spectrum of complex **3b** in $(CD_3)_2CO$ at 298 K.

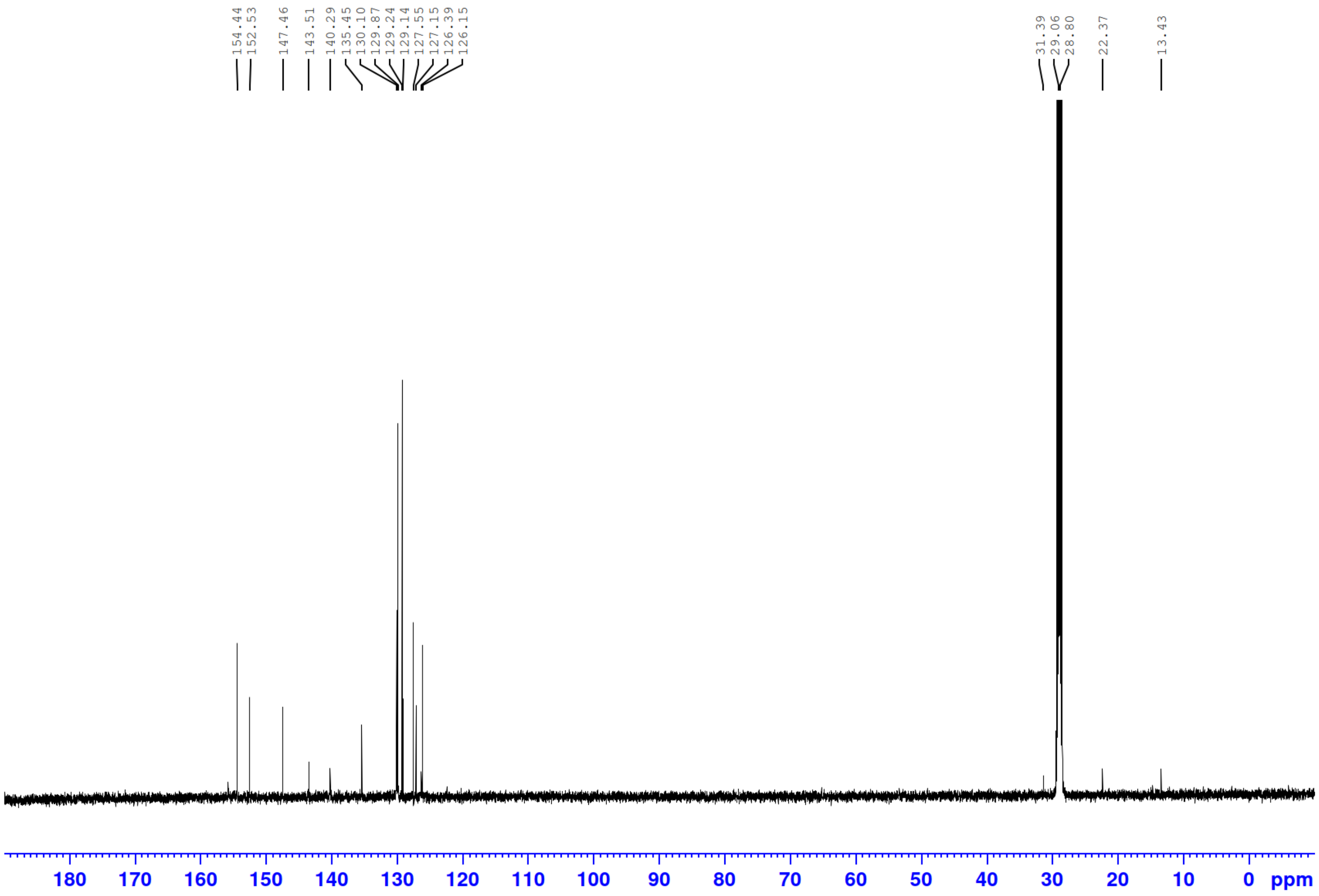

Figure S38. ^{1}H NMR spectrum of ICPr-fluorescein in $CDCl_3$ at 298 K.

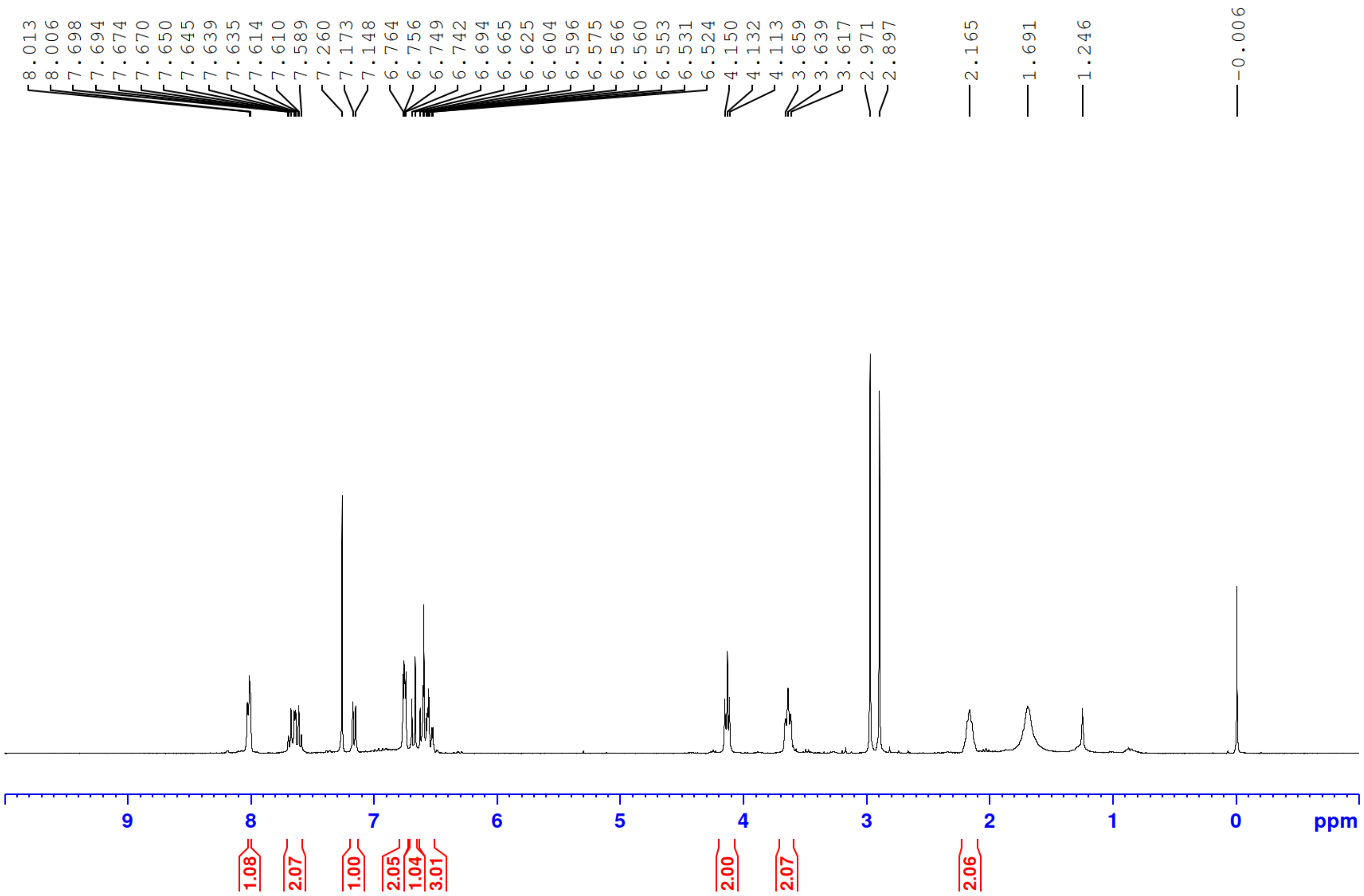

Figure S39. (a) Experimental and (b) simulated HR-ESI mass spectra of complex **1a** in CH_3CN.

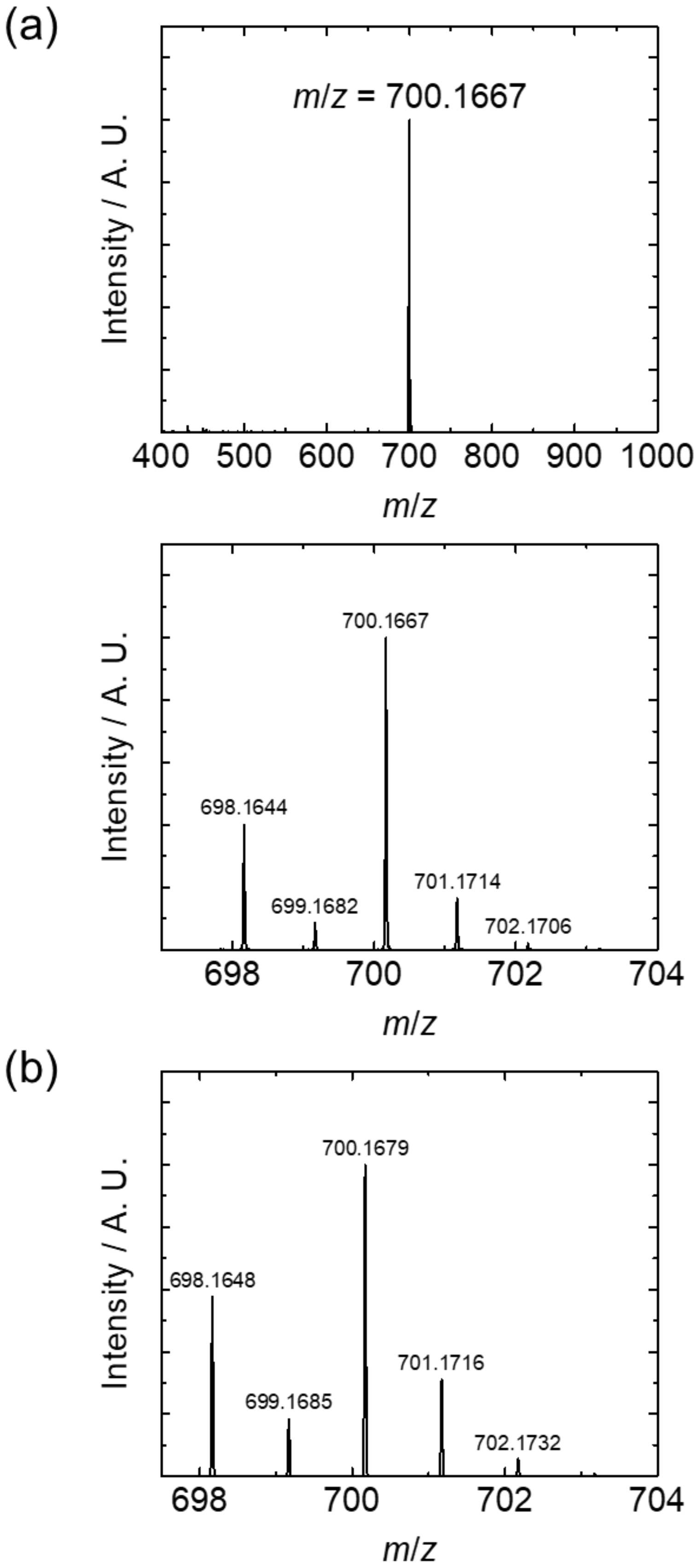

Figure S40. (a) Experimental and (b) simulated HR-ESI mass spectra of complex **1b** in CH_3CN.

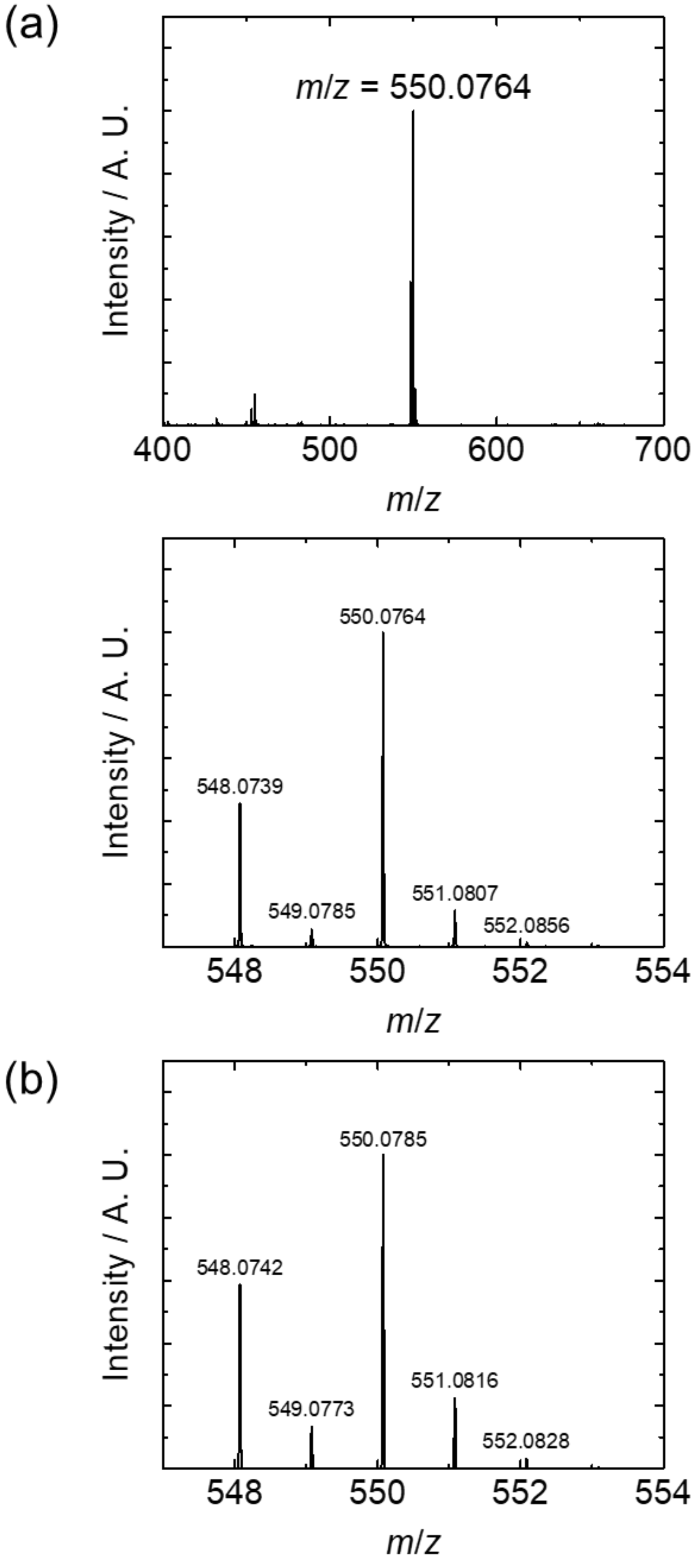

Figure S41. (a) Experimental and (b) simulated HR-ESI mass spectra of complex **2a** in CH_3CN.

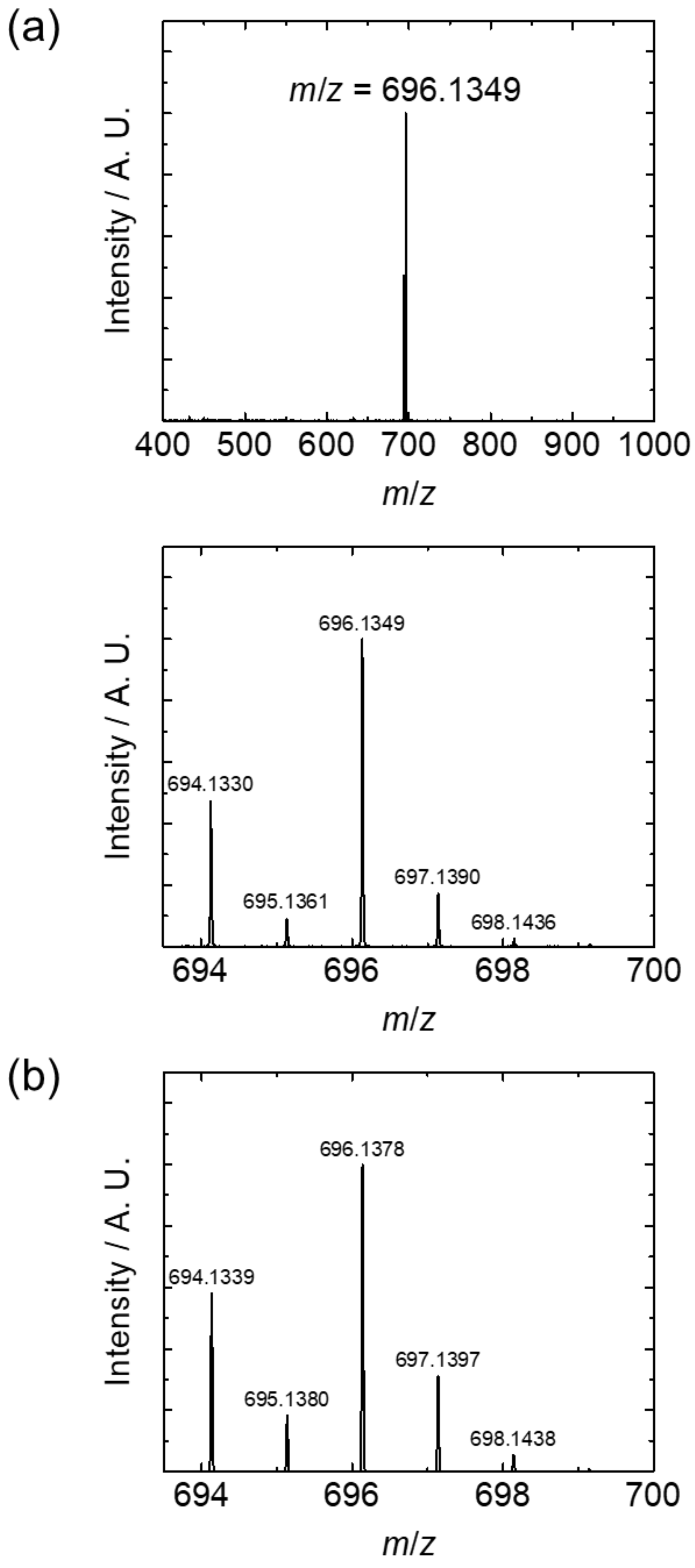

Figure S42. (a) Experimental and (b) simulated HR-ESI mass spectra of complex **2b** in CH_3CN.

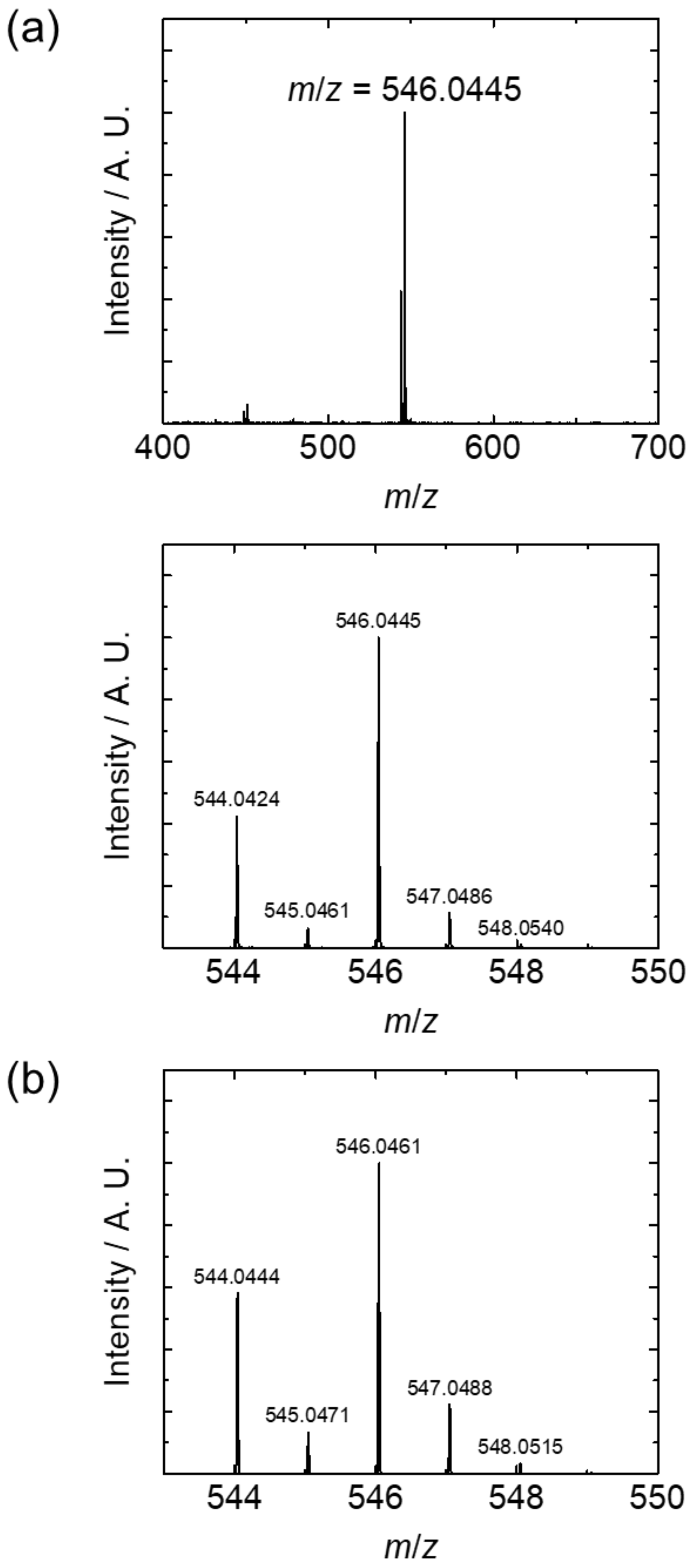

Figure S43. (a) Experimental and (b) simulated HR-ESI mass spectra of complex **3a** in CH_3CN.

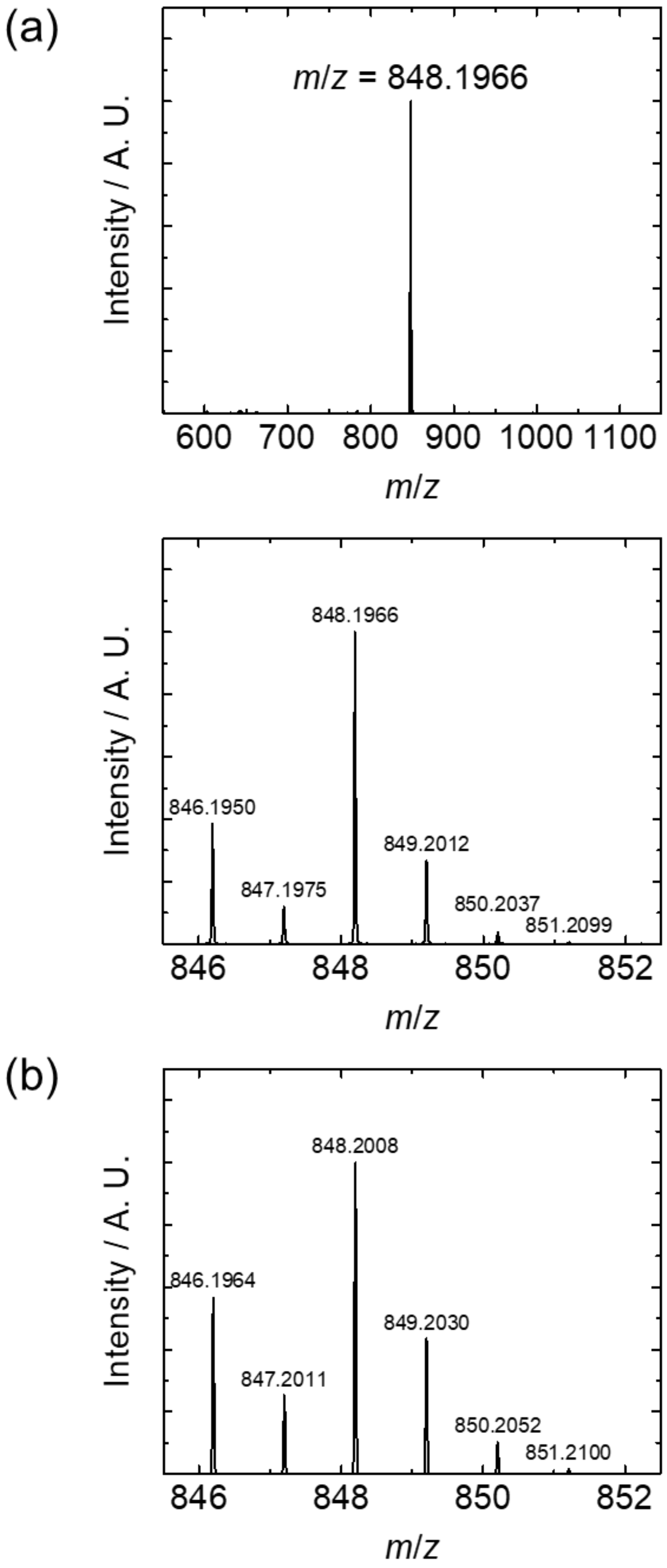

Figure S44. (a) Experimental and (b) simulated HR-ESI mass spectra of complex **3b** in CH_3CN.

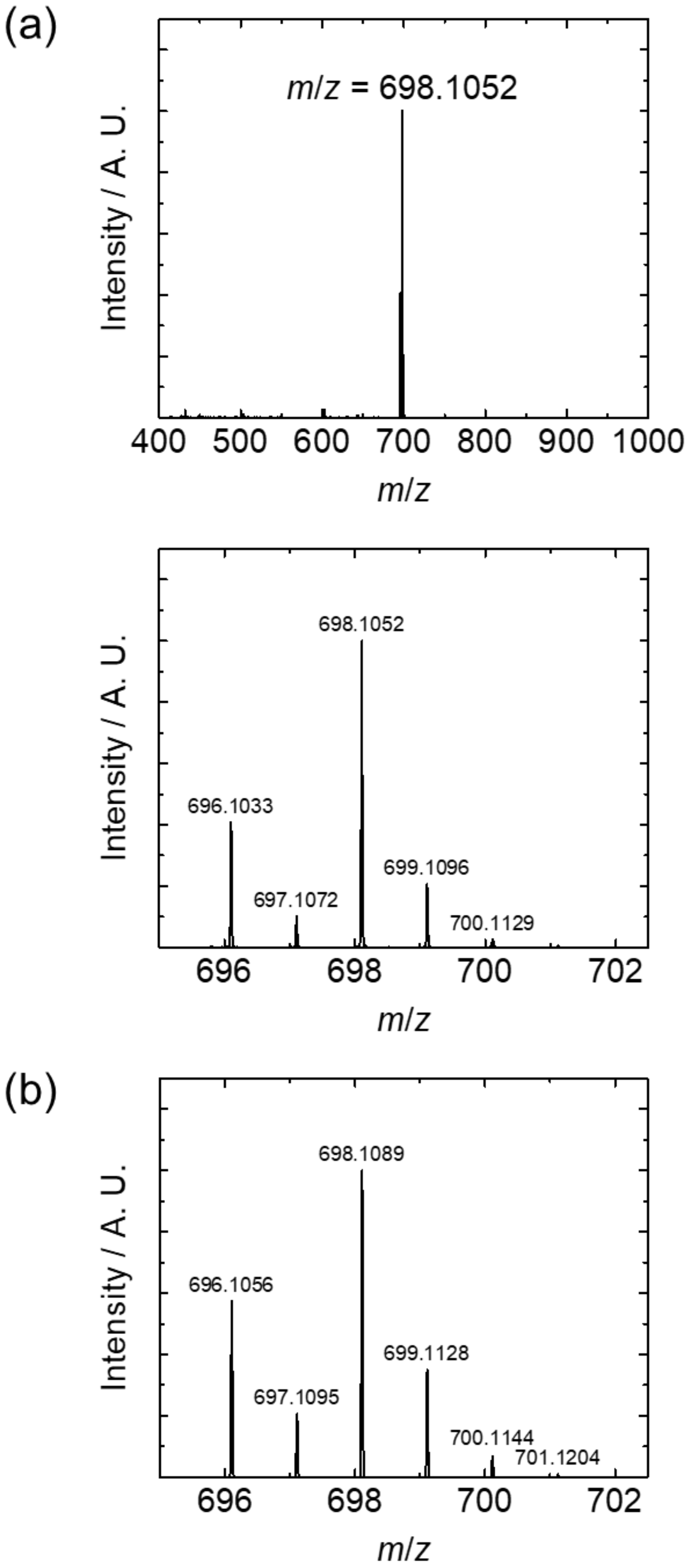

References

1. W. L. F. Armarego, C. Chai, *Purification of Laboratory Chemicals (Seventh Edition)*, Butterworth-Heinemann: Oxford, **2013**.
2. J. V. Caspar, T. J. Meyer, *J. Phys. Chem.* **1983**, *87*, 952.
3. J. Tu, M. Xu, R. M. Franzini, *Synlett* **2020**, *31*, 1701.
4. J. Tu, M. Xu, S. Parvez, R. T. Peterson, R. M. Franzini, *J. Am. Chem. Soc.* **2018**, *140*, 8410.
5. J. N. Demas, G. A. Crosby, *J. Phys. Chem.* **1971**, 75, 991.
6. L. Wallace, D. P. Rillema, *Inorg. Chem.* **1993**, *32*, 3836.
7. R. W. Redmond, J. N. Gamblin, *Photochem. Photobiol.* **1999**, *70*, 391.
8. L. He, Y. Li, C.-P. Tan, R.-R. Ye, M.-H. Chen, J.-J. Cao, L.-N. Ji, Z.-W. Mao, *Chem. Sci.* **2015**, *6*, 5409.
9. J. Baier, T. Maisch, M. Maier, E. Engel, M. Landthaler, W. Bäumler, *Biophys. J.* **2006**, *91*, 1452.
